# Successful Treatment of *Balamuthia mandrillaris* Granulomatous Amebic Encephalitis with Nitroxoline

**DOI:** 10.3201/eid2901.221531

**Published:** 2023-01

**Authors:** Natasha Spottiswoode, Douglas Pet, Annie Kim, Katherine Gruenberg, Maulik Shah, Amrutha Ramachandran, Matthew T. Laurie, Maham Zia, Camille Fouassier, Christine L. Boutros, Rufei Lu, Yueyuan Zhang, Venice Servellita, Andrew Bollen, Charles Y. Chiu, Michael R. Wilson, Liza Valdivia, Joseph L. DeRisi

**Affiliations:** University of California, San Francisco, California, USA (N. Spottiswoode, D. Pet, A. Kim, K. Gruenberg, M. Shah, Amrutha Ramachandran, Matthew T. Laurie, Maham Zia, Camille Fouassier, Christine L. Boutros, Rufei Lu, Yueyuan Zhang, Venice Servellita, Andrew Bollen, Charles Y. Chiu, Michael R. Wilson, Liza Valdivia, Joseph L. DeRisi);; Chan Zuckerberg Biohub, San Francisco (Joseph L. DeRisi)

**Keywords:** Infectious encephalitis, ameba, ameba drug effects, nitroxoline, Balamuthia mandrillaris, granulomatous amebic encephalitis, meningitis/encephalitis, parasites

## Abstract

A patient in California, USA, with rare and usually fatal *Balamuthia mandrillaris* granulomatous amebic encephalitis survived after receiving treatment with a regimen that included the repurposed drug nitroxoline. Nitroxoline, which is a quinolone typically used to treat urinary tract infections, was identified in a screen for drugs with amebicidal activity against *Balamuthia.*

*Balamuthia mandrillaris* is an ameba that can cause skin lesions, endophthalmitis, or granulomatous amebic encephalitis (GAE) in previously healthy patients. Routes of infection include possible soil exposure (*1*) and transplanted organs from infected donors (*2*,*3*). The mortality rate is high. During 1974–2016, of 109 *Balamuthia* GAE cases in the United States, 10 patients survived; mortality rate was >90% (*4*).

The recommended medication regimen for *Balamuthia* GAE includes pentamidine, sulfadiazine, azithromycin/clarithromycin, fluconazole, flucytosine, and miltefosine (*5*). This regimen is based on survivor case series, and results have been inconsistent. Of those medications, only miltefosine, pentamidine, and azithromycin exhibit in vitro evidence of amebicidal or amebistatic activity (*6*,*7*). Furthermore, in vitro activity requires high concentrations of drug that are implausible in vivo (50% inhibitory concentration for azithromycin 244 μM and miltefosine 62 μM) (*6*). We report a patient with *B. mandrillaris* granulomatous amebic encephalitis who survived after receiving treatment with nitroxoline, a drug typically used to treat urinary tract infections, which was identified in a screen for drugs with amebicidal activity against *Balamuthia.*

## The Case

**Figure 1:**
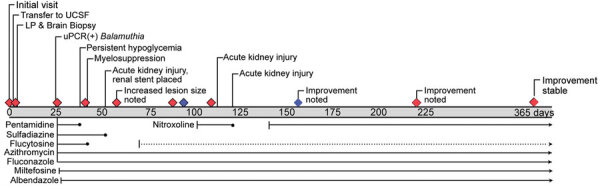
Timeline of events and medications for patient with granulomatous amebic encephalitis, California, USA. Grey bar shows days since initial evaluation; diamonds indicate interval magnetic resonance images; blue diamonds indicate magnetic resonance images taken before and after administration of nitroxoline. Medications at the bottom are other treatments administered. Solid lines refer to the dosages indicated in article text, and dotted lines indicate dose reduction. LP, lumbar puncture; UCSF, University of California, San Francisco Medical Center, San Francisco, California, USA; uPCR, universal broad-range PCR amplicon sequencing.

The patient was a man in his 50s, with no remarkable medical history, who received care at a hospital in northern California, USA, after experiencing a generalized seizure. Magnetic resonance imaging (MRI) demonstrated a solitary left temporal lobe T2 hyperintensity with gadolinium rim enhancement and surrounding edema. After receiving treatment with dexamethasone and levetiracetam, he was transferred to the University of California San Francisco Medical Center (UCSF) (Figure 1).

**Figure 2:**
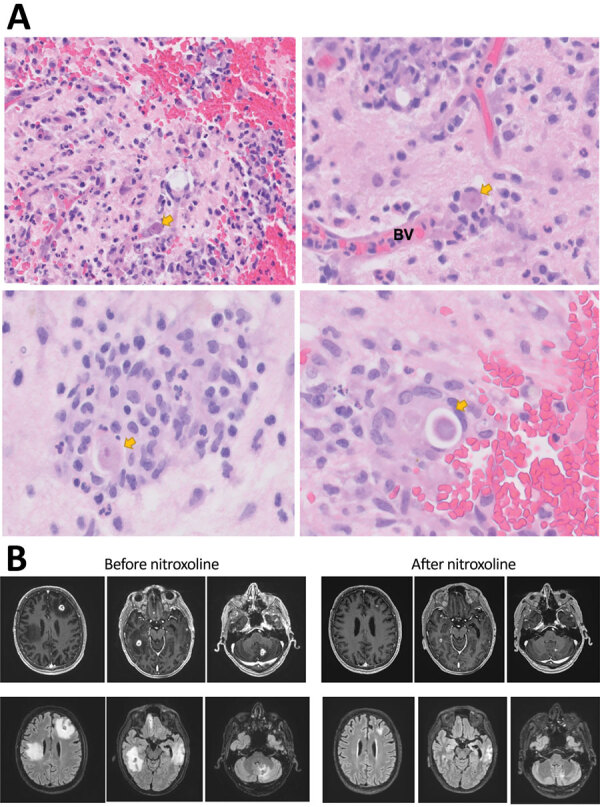
Diagnostic findings for patient with granulomatous amebic encephalitis, California, USA. A) Brain biopsy sample. Granulomas are noted in a perivascular pattern. Scattered structures (arrows) with large nuclei and abundant cytoplasma are concerning for amebic trophozoite forms. Occasional structures with a large nucleus present within a relatively rigid outline (lower right image) are suspicious for amebic cysts, the dormant, thick-walled life stage. B) Magnetic resonance images obtained before and after nitroxoline treatment. Upper row shows axial gadolinium-enhanced T1-weighted images; lower row shows axial fluid-attenuated inversion recovery images. Images in the left series were obtained on day 96 after initial visit, 1 week before nitroxoline initiation; images in the right series were obtained on day 156 after initial visit, 7 weeks after nitroxoline initiation. BV, blood vessel.

Examination by neurology consultants indicated disorientation, inattention, moderate aphasia, and mild right hemiparesis. The aphasia and hemiparesis improved during hospitalization, suggesting that those signs were postictal. Cerebrospinal fluid (CSF) testing revealed increased nucleated cells up to 80/UL (60% lymphocytes, 17% neutrophils, 23% monocytes), protein concentration 38 mg/dL, and glucose concentration 100 mg/dL. A biopsy sample from the left temporal lobe lesion was preliminarily reported as well-formed granulomata with acute inflammation (Figure 2, panel A**)**.

Cultures from the brain biopsy sample did not grow bacteria, fungi, or mycobacteria. We performed metagenomic next-generation sequencing (mNGS) on a CSF sample (*8*,*9*) and sent brain biopsy samples for universal broad-range PCR amplicon sequencing (uPCR) for bacteria, fungi, *Mycobacterium tuberculosis*, and nontuberculous mycobacteria. Rereview of neuropathology raised concern for amebic forms, prompting us to add uPCR of brain biopsy sample for ameba species (*10*). The patient was discharged. 

On day 26 after the initial visit, Sanger sequencing of the uPCR amplicon was positive for *B. mandrillaris*. This diagnosis was confirmed by research-based mNGS of DNA extracted from the formalin-fixed, paraffin-embedded brain tissue under a remnant clinical sample biobanking protocol (UCSF IRB #10-01116; Appendix) (*11*). Results of all other studies, including mNGS of CSF, were negative.

On day 27, the patient was rehospitalized, again with disorientation and inattention. Repeat MRI showed disease progression with multiple new supratentorial and infratentorial lesions consistent with amebic abscesses. After consulting with the Centers for Disease Control and Prevention, we started administering sulfadiazine (1,500 mg orally 4×/d), fluconazole (1,000 mg [12 mg/kg] orally 1×/d), flucytosine (3,000 mg [37.5 mg/kg] 4×/d), pentamidine (330 mg [4 mg/kg] intravenously 1×/d), azithromycin (500 mg orally 1×/d), miltefosine (50 mg orally 3×/d), and albendazole (400 mg orally 1×/d) (Figure 1).

MRI on day 42 (15 days of the 7-drug regimen) showed moderately reduced lesion size, reduced edema, and no new lesions. However, severe medication toxicities developed. Hypoglycemia required discontinuation of pentamidine. Subsequent renal failure caused by sloughing of renal calyces required discontinuation of sulfadiazine and placement of bilateral nephrostomy tubes. The patient’s kidney function improved but remained impaired (baseline estimated glomerular filtration rate 20). Decreased absolute neutrophil count necessitated stopping and then reducing flucytosine dose. The ongoing limited regimen included dose-reduced flucytosine, fluconazole, miltefosine, albendazole, and azithromycin. Brain MRI on day 59 (14 days on this limited regimen) showed interval increase in lesion size, with increased edema**.**

Given his worsening prognosis, the patient and his family agreed to trial treatment with nitroxoline, a quinolone antibiotic with in vitro amebicidal activity against *B. mandrillaris* (*6*). Nitroxoline was selected instead of compounds identified in other high-throughput screens (*12*) because it is available internationally to treat urinary tract infections and is well tolerated (*13*). Nitroxoline was authorized through an emergency use authorization (Food and Drug Administration Investigational New Drug no. 154939), and on day 103, we initiated treatment (250 mg orally 3×/d). All other medications were continued. The patient experienced a transient acute kidney injury; although the nephrology staff considered it unrelated to nitroxoline, we submitted a possible adverse event report.

On day 109, after 1 week of nitroxoline treatment, MRI showed decreased size of the cerebral abscesses and no new lesions compared with MRI before nitroxoline on day 96 **(**Figure 2, panel B). The patient was discharged, although neurologic examination continued to demonstrate mild disorientation and inattention. Recovery was complicated by a subsequent acute kidney injury because of malfunctioning ureteral stents, and nitroxoline administration was stopped while creatinine clearance was <20 (days 122–143). Interval MRIs on days 156 and 220 (7 and 17 weeks after nitroxoline initiation) showed continued marked improvement in lesion size (Figure 2, panel B). 

As of 15 months after initial evaluation, the patient continues to take nitroxoline, miltefosine, azithromycin, albendazole, fluconazole, and dose-reduced flucytosine. His infectious disease outpatient clinicians plan to sequentially discontinue medications after 1 year. He lives in the community, and his family assists with medication management and appointments.

## Conclusions

Repurposed use of nitroxoline associated with survival from *B. mandrillaris* GAE demonstrates the potential of basic research to identify antiamebic agents that improve outcome of this rare and deadly disease. Given the limited options for treating *Balamuthia* GAE, several studies have used high-throughput screening tools to identify other amebicidal agents. Compounds from the Medicines for Malaria Ventures Pandemic Response Box underwent in vitro trials against *B. mandrillaris*, but the leading candidates have not been used in humans or are not commercially available (*12*). In the screening for the patient reported here, which identified nitroxoline as having amebicidal activity against *B. mandrillaris*, in vitro efficacy of nitroxoline against *B. mandrillaris* cysts and trophozoites was determined to be higher than that of currently recommended drugs and prevented tissue destruction in fibroblast and explant models of *B. mandrillaris* infection (*6*).

In vitro efficacy of nitroxoline is better than that of other antiamebic medications; it also is safe and well tolerated. In a review of its use in urinary tract infections, 9.8% of patients reported adverse events, primarily nausea (*13*). In contrast, current antiamebic treatments can cause severe toxicity, especially pentamidine and sulfadiazine. It is not known if nitroxoline penetrates the CNS, but our limited experience suggests that it may, especially in a patient with a compromised blood–brain barrier.

A barrier to treating *Balamuthia* GAE is diagnostic delay. Imaging characteristics are nonspecific, and more common pathologies are often misdiagnosed (e.g., neoplasm or pyogenic abscesses) (*4*). CSF findings are typically nonspecific, direct detection tests of CSF can be insensitive, and visualization of organisms in CSF is rare. Thus, diagnosis often requires brain biopsy, specialized pathology, or dedicated PCR testing.

Unbiased diagnostics, such as mNGS, offer the opportunity to streamline and accelerate diagnosis of rare pathogens such as *Balamuthia* (*8*). Multiple *Balamuthia* GAE cases have been diagnosed by CSF mNGS (*14*,*15*). In the case we report, results of CSF mNGS testing were negative, concordant with negative CSF uPCR and indicating that no abscesses had ruptured or were abutting the ventricles (*8*). However, results of uPCR and research-based mNGS of brain tissue were positive. Currently, clinical mNGS is not available for brain biopsy samples; clinical validation of this test may speed diagnoses of future cases. Despite the limitations of any case study, we suggest that accelerated diagnosis with unbiased techniques and early initiation of nitroxoline may offer promise to improve survival rates for patients with *Balamuthia* GAE.

Appendix Additional information for case report of successful treatment of *Balamuthia mandrillaris* granulomatous amebic encephalitis with nitroxoline.
