# Supplementary material for: Successful Treatment of Balamuthia mandrillaris Granulomatous Amebic Encephalitis with Nitroxoline
Source: Emerg Infect Dis. 2023 Jan;29(1):197–201. doi: 10.3201/eid2901.221531 (PMC9796214; doi:10.3201/eid2901.221531)
Supplement: Appendix — Additional information for case report of successful treatment of Balamuthia mandrillaris granulomatous amebic encephalitis with nitroxoline. [file 22-1531-Techapp-s1.pdf]

Article DOI: https://doi.org/10.3201/eid2901.221531

Successful Treatment of *Balamuthia mandrillaris* Granulomatous Amebic Encephalitis with Nitroxoline

Appendix

Metagenomic Next-Generation Sequencing of DNA Extracted from Formalin-Fixed, Paraffin-Embedded Brain Tissue from Patient with *Balamuthia Mandrillaris* Granulomatous Amebic Encephalitis

>family_nr:-300:family_nt:555408:genus_nr:-200:genus_nt:66526:species_
nr:-100:species_nt:66527:NR::NT:KF725772.1:VH00444:1:AAAMMTVHV:1:1102:
65021:48347/1
ATTTTCGGTTCTACAAAACTTGTAGCCCAAATTGAGGTGGTCAATTTGGGCTAGCTATGTGCCAGGCGTT
GCATGGCTGTCGTAAGCTCGTGTCTTAGGATGTGT
>family_nr:-300:family_nt:555408:genus_nr:-200:genus_nt:66526:species_
nr:-100:species_nt:66527:NR::NT:KP888565.1:VH00444:1:AAAMMTVHV:1:1101:
60760:14214/1
AAGCACTATTATTTCGCGTTTGATTTTATTCACAAGCTTTAAGTATTTTCTACGCTTGTTTATACAGATA
CCGTGGAATGTA
>family_nr:-300:family_nt:555408:genus_nr:-200:genus_nt:66526:species_
nr:-100:species_nt:66527:NR::NT:KP888565.1:VH00444:1:AAAMMTVHV:1:1307:
58924:51679/2
TTTTTCTACGAGTGGTTTAAAGGCGCTCTTTTATGGGAATAGAGAATGGAACCACGTTTACAGCTGCTTG
TGCAATTATTATTAAAAAG
>family_nr:-300:family_nt:555408:genus_nr:-200:genus_nt:66526:species_
nr:-100:species_nt:66527:NR::NT:KP888565.1:VH00444:1:AAAMMTVHV:1:1606:
19992:30514/2
GTCTTTTTTCCGTGTACTTTCCTTCTGCATTGGAACGTGTTCAATGAAAAAAAATTACAGAATCGGCCAT
TATACTTTATAATTTATAAGTTTTTTTCATATTTATGAAATTTTTTT
>family_nr:-300:family_nt:555408:genus_nr:-200:genus_nt:66526:species_
nr:-100:species_nt:66527:NR::NT:KP888565.1:VH00444:1:AAAMMTVHV:1:2113:
72595:17868/1
ATTAAGGCCATTGTGGTAAATCGTATTGGTGATTTTGGTCTGTATTTTGGCATTCTCCT

>family_nr:-300:family_nt:555408:genus_nr:-200:genus_nt:66526:species_
nr:-100:species_nt:66527:NR::NT:KP990616.1:VH00444:1:AAAMMTVHV:1:1102:
65021:48347/2
ACACATCCTAAGACACGAGCTTACGACAGCCATGCAACGCCTGGCACATAGCTAGCCCAAATTGACCACC
TCAATTTGGGCTACAAGTTTTGTAGAACCGAAAAT
>family_nr:-300:family_nt:555408:genus_nr:-200:genus_nt:66526:species_
nr:-100:species_nt:66527:NR::NT:KP990616.1:VH00444:1:AAAMMTVHV:1:1305:
44930:6377/1
GTCCATGCCCTGACCTATGAATATTAATCGTCCATTTAGATAGTGGATGACTAAGCTAACGCGAAAATAT
TCCACCTGAGGACTACGGCCGCAAGGTTAAAACTCAAAGAAAT
>family_nr:-300:family_nt:555408:genus_nr:-200:genus_nt:66526:species_
nr:-100:species_nt:66527:NR::NT:KR908788.1:VH00444:1:AAAMMTVHV:1:2505:
24423:21693/1
CTTGCGGCCGTAGTCCTCAGGTGGAATATTTTCGCGTTAGCTTAGTCATCCACTATCTAAATGGACGATT
>family_nr:-300:family_nt:555408:genus_nr:-200:genus_nt:66526:species_
nr:-100:species_nt:66527:NR::NT:KT030670.1:VH00444:1:AAAMMTVHV:1:1206:
25484:9046/2
CTTACTTGCTTGTTTATAATACGCAGAAAACAAAATAAAATTTTTTGTCCAGCAGCAGGTTCCCCTACCA
CTACCTTGTT
>family_nr:-300:family_nt:555408:genus_nr:-200:genus_nt:66526:species_
nr:-100:species_nt:66527:NR::NT:KT030671.1:VH00444:1:AAAMMTVHV:1:1307:
58924:51679/1
CTTTTTAATAATAATTGCACAAGCAGCTGTAAACGTGGTTCCATTCTCTATTCCCATAAAAGAGCGCCTT
TAAACCACTCGTAGAAAAA
>family_nr:-300:family_nt:555408:genus_nr:-200:genus_nt:66526:species_

nr:-100:species_nt:66527:NR::NT:KT030671.1:VH00444:1:AAAMMTVHV:1:1309:
38814:37102/2
ATTCTGTAATTAACGGTGAGTAAACGCAGATAAGACCTTTGAGCTTTTAATTATGAAC
>family_nr:-300:family_nt:555408:genus_nr:-200:genus_nt:66526:species_
nr:-100:species_nt:66527:NR::NT:KT030671.1:VH00444:1:AAAMMTVHV:1:1408:
36750:19591/1
ATAAAAGCATGATTTACGCCACAAAGTTCGCTACACTGACCATAATAAC
>family_nr:-300:family_nt:555408:genus_nr:-200:genus_nt:66526:species_
nr:-100:species_nt:66527:NR::NT:KT030671.1:VH00444:1:AAAMMTVHV:1:1510:
70929:50921/1
GTAATCTATGTGTGCGTAACAGAATAGGGGGTAGCGACTGTTTAATAAAAACACAGGACTCTGCTAAATT
GAAAAATGATGTATAGAG
>family_nr:-300:family_nt:555408:genus_nr:-200:genus_nt:66526:species_
nr:-100:species_nt:66527:NR::NT:KT030671.1:VH00444:1:AAAMMTVHV:1:1606:
19992:30514/1
AAAAAAATTTCATAAATATGAAAAAAACTTATAAATTATAAAGTATAATGGCCGATTCTGTAATTTTTTT
TCATTGAACACGTTCCAATGCAGAAGGAAAGTACACGGAAAAAAGAC
>family_nr:-300:family_nt:555408:genus_nr:-200:genus_nt:66526:species_
nr:-100:species_nt:66527:NR::NT:KT030671.1:VH00444:1:AAAMMTVHV:1:2209:
8707:16770/2
ATTAAAGTGGTACGTGAGTTGGGTTTAGAGTTATTCTACATAAACACAACAAACGTACAATCCCGTGTTA
TAGTAAAGGTGTACAGGGTCTTTCCGTCTACTTAC
>family_nr:-300:family_nt:555408:genus_nr:-200:genus_nt:66526:species_
nr:-100:species_nt:66527:NR::NT:KT030671.1:VH00444:1:AAAMMTVHV:1:2312:
23647:24778/2
TTTGTTTACCTGTAAATCACGTCGCATTTTAAATTTTTAAAGATACAG
>family_nr:-300:family_nt:555408:genus_nr:-200:genus_nt:66526:species_
nr:-100:species_nt:66527:NR::NT:KT030672.1:VH00444:1:AAAMMTVHV:1:1204:
21659:40245/1
GTTTTTAATCAATGAGCTTCTATTGGATCGCTCATATCTTTGTCAAGTATTTTCTTTTTTTTCTTTTTAA
TTTTCGTGTCGTT
>family_nr:-300:family_nt:555408:genus_nr:-200:genus_nt:66526:species_
nr:-100:species_nt:66527:NR::NT:KT030672.1:VH00444:1:AAAMMTVHV:1:1305:
44930:6377/2
ATTTCTTTGAGTTTTAACCTTGCGGCCGTAGTCCTCAGGTGGAATATTTTCGCGTTAGCTTAGTCATCCA
CTATCTAAATGGACGATTAATATTCATAGGTCAGGGCATGGAC
>family_nr:-300:family_nt:555408:genus_nr:-200:genus_nt:66526:species_
nr:-100:species_nt:66527:NR::NT:KT030672.1:VH00444:1:AAAMMTVHV:1:2202:
61480:53931/1
ATTCTCAGCGCCCAAAGGAACAAGAACCAAAATTTTTTTAACAAACGTTCTTTTTACT
>family_nr:-300:family_nt:555408:genus_nr:-200:genus_nt:66526:species_
nr:-100:species_nt:66527:NR::NT:KT030672.1:VH00444:1:AAAMMTVHV:1:2208:
28911:50675/1
CATGGGAATTTTATGCCCATTAATGTTACTTCGATCCTCATATGTTACTCACCAAGACACCAC
>family_nr:-300:family_nt:555408:genus_nr:-200:genus_nt:66526:species_
nr:-100:species_nt:66527:NR::NT:KT030673.1:VH00444:1:AAAMMTVHV:1:1101:
60760:14214/2
TACATTCCACGGTATCTGTATAAACAAGCGTAGAAAATACTTAAAGCTTGTGAATAAAATCAAACGCGAA
ATAATAGTGCTT
>family_nr:-300:family_nt:555408:genus_nr:-200:genus_nt:66526:species_

nr:-100:species_nt:66527:NR::NT:KT030673.1:VH00444:1:AAAMMTVHV:1:1214:
39212:44996/1
CACAAACGGAATGCGGAAATATCCCCCAGTGAACTTGAAATACGATGGACGAAAA
>family_nr:-300:family_nt:555408:genus_nr:-200:genus_nt:66526:species_
nr:-100:species_nt:66527:NR::NT:KT030673.1:VH00444:1:AAAMMTVHV:1:1214:
39212:44996/2
TTTTCGTCCATCGTATTTCAAGTTCACTGGGGGATATTTCCGCATTCCGTTTGTG
>family_nr:-300:family_nt:555408:genus_nr:-200:genus_nt:66526:species_
nr:-100:species_nt:66527:NR::NT:KT030673.1:VH00444:1:AAAMMTVHV:1:2113:
72595:17868/2
AGGAGAATGCCAAAATACAGACCAAAATCACCAATACGATTTACCACAATGGCCTTAAT
>family_nr:-300:family_nt:555408:genus_nr:-200:genus_nt:66526:species_
nr:-100:species_nt:66527:NR::NT:KT030673.1:VH00444:1:AAAMMTVHV:1:2213:
38871:37235/1
ATGAACAAATCCTGCATCATTTTACTCATTAAGCTTATAGTTTATTACAAAAAAACGGGACTTGAACCAA
CCTACGGTCTGAAATATACTCAGTTTGTTCATAATTAAAAGCTCAAAGGTCTTATCTGCGTTTACTCACC
GTTAATT
>family_nr:-300:family_nt:555408:genus_nr:-200:genus_nt:66526:species_
nr:-100:species_nt:66527:NR::NT:KT030673.1:VH00444:1:AAAMMTVHV:1:2312:
23647:24778/1
CTGTATCTTTAAAAATTTAAAATGCGACGTGATTTACAGGTAAACAAA
>family_nr:-300:family_nt:555408:genus_nr:-200:genus_nt:66526:species_
nr:-100:species_nt:66527:NR::NT:KT030673.1:VH00444:1:AAAMMTVHV:1:2411:
40253:42213/2
ATCAAAATTCTACGGCTACAATATAAACAAAAAAAAAAC
>family_nr:-300:family_nt:555408:genus_nr:-200:genus_nt:66526:species_
nr:-100:species_nt:66527:NR::NT:KT030673.1:VH00444:1:AAAMMTVHV:1:2505:
24423:21693/2
AATCGTCCATTTAGATAGTGGATGACTAAGCTAACGCGAAAATATTCCACCTGAGGACTACGGCCGCAAG
>family_nr:-300:family_nt:555408:genus_nr:-200:genus_nt:66526:species_
nr:-100:species_nt:66527:NR::NT:KT175738.1:VH00444:1:AAAMMTVHV:1:1113:
20693:47154/2
GGTGTATGAATACAAAATAAGATACGTGCGCCGCTATTGTCTGCTACTTTTAAAAC
>family_nr:-300:family_nt:555408:genus_nr:-200:genus_nt:66526:species_
nr:-100:species_nt:66527:NR::NT:KT175738.1:VH00444:1:AAAMMTVHV:1:1504:
62786:33430/1
ATAAGGAGCTAAAAATTGTAACGGCGCCGCATCAGAAGCAGTAGCTGCCACAATAACAG
>family_nr:-300:family_nt:555408:genus_nr:-200:genus_nt:66526:species_
nr:-100:species_nt:66527:NR::NT:KT175738.1:VH00444:1:AAAMMTVHV:1:2411:
40253:42213/1
GTTTTTTTTTTGTTTATATTGTAGCCGTAGAATTTTGAT
>family_nr:-300:family_nt:555408:genus_nr:-200:genus_nt:66526:species_
nr:-100:species_nt:66527:NR::NT:KT175739.1:VH00444:1:AAAMMTVHV:1:1201:
54133:1492/1
TGATTATTTACGTTATGTTTATAACCTTGACGGAATCGTTCTTCGACTTGA
>family_nr:-300:family_nt:555408:genus_nr:-200:genus_nt:66526:species_
nr:-100:species_nt:66527:NR::NT:KT175739.1:VH00444:1:AAAMMTVHV:1:1309:
38814:37102/1
GTTCATAATTAAAAGCTCAAAGGTCTTATCTGCGTTTACTCACCGTTAATTACAGAAT
>family_nr:-300:family_nt:555408:genus_nr:-200:genus_nt:66526:species_

nr:-100:species_nt:66527:NR::NT:KT175739.1:VH00444:1:AAAMMTVHV:1:1504:
62786:33430/2
CTGTTATTGTGGCAGCTACTGCTTCTGATGCGGCGCCGTTACAATTTTTAGCTCCTTAT
>family_nr:-300:family_nt:555408:genus_nr:-200:genus_nt:66526:species_
nr:-100:species_nt:66527:NR::NT:KT175739.1:VH00444:1:AAAMMTVHV:1:1507:
25332:54897/1
TGTTAACTAAAACCACCGAATTCGATGAAGCACTAAAAAAAAAGTTACCTCAC
>family_nr:-300:family_nt:555408:genus_nr:-200:genus_nt:66526:species_
nr:-100:species_nt:66527:NR::NT:KT175739.1:VH00444:1:AAAMMTVHV:1:2209:
8707:16770/1
GTAAGTAGACGGAAAGACCCTGTACACCTTTACTATAACACGGGATTGTACGTTTGTTGTGTTTATGTAG
AATAACTCTAAACCCAACTCACGTACCACTTTAAT
>family_nr:-300:family_nt:555408:genus_nr:-200:genus_nt:66526:species_
nr:-100:species_nt:66527:NR::NT:KT175739.1:VH00444:1:AAAMMTVHV:1:2509:
36504:43501/2
ATTGCGTATTTTATTTTTTTTTTTTTTAGCGCTTTCTGTGATTCTGTTTTTCGCTTCATTT
>family_nr:-300:family_nt:555408:genus_nr:-200:genus_nt:66526:species_
nr:-100:species_nt:66527:NR::NT:KT175739.1:VH00444:1:AAAMMTVHV:1:2603:
56519:37954/1
CTAGTGTGTTATTTTTCAAATAAGAATTGCGAAATAAAATATTTAAAACAGGAG
>family_nr:-300:family_nt:555408:genus_nr:-200:genus_nt:66526:species_
nr:-100:species_nt:66527:NR::NT:KT175739.1:VH00444:1:AAAMMTVHV:1:2613:
49248:48063/2
CATAAATTTAGAATCGCTGAAAGCATATAAGCGAGAAAATGTTTTT
>family_nr:-300:family_nt:555408:genus_nr:-200:genus_nt:66526:species_
nr:-100:species_nt:66527:NR::NT:KT175740.1:VH00444:1:AAAMMTVHV:1:1108:
69338:33316/2
AATACAAAACCGGTGAACCAAACGGCGGCGTAAGTACGGCACAAACGGTTTTCGGACTAAC
>family_nr:-300:family_nt:555408:genus_nr:-200:genus_nt:66526:species_
nr:-100:species_nt:66527:NR::NT:KT175740.1:VH00444:1:AAAMMTVHV:1:1408:
36750:19591/2
GTTATTATGGTCAGTGTAGCGAACTTTGTGGCGTAAATCATGCTTTTAT
>family_nr:-300:family_nt:555408:genus_nr:-200:genus_nt:66526:species_
nr:-100:species_nt:66527:NR::NT:KT175740.1:VH00444:1:AAAMMTVHV:1:1507:
25332:54897/2
GTGAGGTAACTTTTTTTTTAGTGCTTCATCGAATTCGGTGGTTTTAGTTAACA
>family_nr:-300:family_nt:555408:genus_nr:-200:genus_nt:66526:species_
nr:-100:species_nt:66527:NR::NT:KT175740.1:VH00444:1:AAAMMTVHV:1:2208:
28911:50675/2
GTGGTGTCTTGGTGAGTAACATATGAGGATCGAAGTAACATTAATGGGCATAAAATTCCCATG
>family_nr:-300:family_nt:555408:genus_nr:-200:genus_nt:66526:species_
nr:-100:species_nt:66527:NR::NT:KT175740.1:VH00444:1:AAAMMTVHV:1:2613:
49248:48063/1
AAAAACATTTTCTCGCTTATATGCTTTCAGCGATTCTAAATTTATG
>family_nr:-300:family_nt:555408:genus_nr:-200:genus_nt:66526:species_
nr:-100:species_nt:66527:NR::NT:KT175741.1:VH00444:1:AAAMMTVHV:1:1108:
39666:36061/1
CAAGCATTTTATTATTAACACGGGCGCTTAATGTTATTAACACTAATGCAAACCCGTAGTTGCCTGACCA
ATAACCCATCTAGTCTTATTTTATAAATAGAG
>family_nr:-300:family_nt:555408:genus_nr:-200:genus_nt:66526:species_

nr:-100:species_nt:66527:NR::NT:KT175741.1:VH00444:1:AAAMMTVHV:1:1108:
39666:36061/2
CTCTATTTATAAAATAAGACTAGATGGGTTATTGGTCAGGCAACTACGGGTTTGCATTAGTGTTAATAAC
ATTAAGCGCCCGTGTTAATAATAAAATGCTTG
>family_nr:-300:family_nt:555408:genus_nr:-200:genus_nt:66526:species_
nr:-100:species_nt:66527:NR::NT:KT175741.1:VH00444:1:AAAMMTVHV:1:1113:
20693:47154/1
GTTTTAAAAGTAGCAGACAATAGCGGCGCACGTATCTTATTTGTATTCATACACC
>family_nr:-300:family_nt:555408:genus_nr:-200:genus_nt:66526:species_
nr:-100:species_nt:66527:NR::NT:KT175741.1:VH00444:1:AAAMMTVHV:1:1201:
22094:20992/1
AAACCCTTTGCGTTGCGCTTCGTAGGTTCGAATCCTACCTCCATCAAAATAAAGGTTGATTGGGGATAGT
TTAATCGGTAAAACGCCGTCCTCCAAAGTCGGTAATATAGGTTCAAGTCCTGTTCCCTTTGTCGTTGTTT
GTTATTC
>family_nr:-300:family_nt:555408:genus_nr:-200:genus_nt:66526:species_
nr:-100:species_nt:66527:NR::NT:KT175741.1:VH00444:1:AAAMMTVHV:1:1201:
22094:20992/2
GTCAAAATCCAACGCCTTACCAATTTGGCTATACTCATCAATGCGACAGGATTTGAACCTATACCGCCTG
TACCCAAAACAGGTACGCTACCAAGTTACGCCACGCACTGAAGAGAATAACAAACAACGACAAAGGGAAC
AGGACTT
>family_nr:-300:family_nt:555408:genus_nr:-200:genus_nt:66526:species_
nr:-100:species_nt:66527:NR::NT:KT175741.1:VH00444:1:AAAMMTVHV:1:1201:
54133:1492/2
TCAAGTCGAAGAACGATTCCGTCAAGGTTATAAACATAACGTAAATAATCA
>family_nr:-300:family_nt:555408:genus_nr:-200:genus_nt:66526:species_
nr:-100:species_nt:66527:NR::NT:KT175741.1:VH00444:1:AAAMMTVHV:1:1202:
54114:21276/1
CAAAAGAAAACTTCTACAAAAAAAAAGAGAAAAGACAAAAAATACAAAAATCAAATTTTTCCCAATTCTA
AAGACCATCCAT
>family_nr:-300:family_nt:555408:genus_nr:-200:genus_nt:66526:species_
nr:-100:species_nt:66527:NR::NT:KT175741.1:VH00444:1:AAAMMTVHV:1:1202:
54114:21276/2
ATGGATGGTCTTTAGAATTGGGAAAAATTTGATTTTTGTATTTTTTGTCTTTTCTCTTTTTTTTTGTAGA
AGTTTTCTTTTG
>family_nr:-300:family_nt:555408:genus_nr:-200:genus_nt:66526:species_
nr:-100:species_nt:66527:NR::NT:KT175741.1:VH00444:1:AAAMMTVHV:1:1204:
21659:40245/2
AACGACACGAAAATTAAAAAGAAAAAAAAGAAAATACTTGACAAAGATATGAGCGATCCAATAGAAGCTC
ATTGATTAAAAAC
>family_nr:-300:family_nt:555408:genus_nr:-200:genus_nt:66526:species_
nr:-100:species_nt:66527:NR::NT:KT175741.1:VH00444:1:AAAMMTVHV:1:1206:
25484:9046/1
AACAAGGTAGTGGTAGGGGAACCTGCTGCTGGACAAAAAATTTTATTTTGTTTTCTGCGTATTATAAACA
ACCAAGTAAG
>family_nr:-300:family_nt:555408:genus_nr:-200:genus_nt:66526:species_
nr:-100:species_nt:66527:NR::NT:KT175741.1:VH00444:1:AAAMMTVHV:1:1206:
26108:9254/1
AACAAGGTAGTGGTAGGGGAACCTGCTGCTGGACAAAAAATTTTATTTTGTTTTCTGCGTATTATAAACA
ACCAAGTAAG
>family_nr:-300:family_nt:555408:genus_nr:-200:genus_nt:66526:species_

nr:-100:species_nt:66527:NR::NT:KT175741.1:VH00444:1:AAAMMTVHV:1:1206:
26108:9254/2
CTTACTTGGTTGTTTATAATACGCAGAAAACAAAATAAAATTTTTTGTCCAGCAGCAGGTTCCCCTACCA
CTACCTTGTT
>family_nr:-300:family_nt:555408:genus_nr:-200:genus_nt:66526:species_
nr:-100:species_nt:66527:NR::NT:KT175741.1:VH00444:1:AAAMMTVHV:1:1211:
77329:27182/1
AGAAGTTATGTCAGCGAACGATTGAGCGACTTTTATAAAGTATAAGTTGAAA
>family_nr:-300:family_nt:555408:genus_nr:-200:genus_nt:66526:species_
nr:-100:species_nt:66527:NR::NT:KT175741.1:VH00444:1:AAAMMTVHV:1:1211:
77329:27182/2
TTTCAACTTATACTTTATAAAAGTCGCTCAATCGTTCGCTGACATAACTTCT
>family_nr:-300:family_nt:555408:genus_nr:-200:genus_nt:66526:species_
nr:-100:species_nt:66527:NR::NT:KT175741.1:VH00444:1:AAAMMTVHV:1:1403:
7116:18133/1
TTGACTGAATTGTGAACTATAGAACGACACGAAAATTAAAAAGTTAATTAGAATTCAATCTCGTCGAGTT
CGCCAATTTATTTATAGGG
>family_nr:-300:family_nt:555408:genus_nr:-200:genus_nt:66526:species_
nr:-100:species_nt:66527:NR::NT:KT175741.1:VH00444:1:AAAMMTVHV:1:1403:
7116:18133/2
CCCTATAAATAAATTGGCGAACTCGACGAGATTGAATTCTAATTAACTTTTTAATTTTCGTGTCGTTCTA
TAGTTCACAATTCAGTCAA
>family_nr:-300:family_nt:555408:genus_nr:-200:genus_nt:66526:species_
nr:-100:species_nt:66527:NR::NT:KT175741.1:VH00444:1:AAAMMTVHV:1:1406:
56538:14309/1
CGAGTAACGTTTAGGTTGGTGAAGAACAACCTCATCAAATATGTAAGGATTCCGATGTAAAGTAACTTTC
ATC
>family_nr:-300:family_nt:555408:genus_nr:-200:genus_nt:66526:species_
nr:-100:species_nt:66527:NR::NT:KT175741.1:VH00444:1:AAAMMTVHV:1:1406:
56538:14309/2
GATGAAAGTTACTTTACATCGGAATCCTTACATATTTGATGAGGTTGTTCTTCACCAACCTAAACGTTAC
TCG
>family_nr:-300:family_nt:555408:genus_nr:-200:genus_nt:66526:species_
nr:-100:species_nt:66527:NR::NT:KT175741.1:VH00444:1:AAAMMTVHV:1:1503:
36599:6263/1
CAACGCCATAAGATGACATACTTTTCAAATTAATTTTCTTGGTTACACGGTTATAAACTAGGTAAAAACT
TCCATGTTAGTGTAACAACAATTAGCTTACAACCCGATTATTGTT
>family_nr:-300:family_nt:555408:genus_nr:-200:genus_nt:66526:species_
nr:-100:species_nt:66527:NR::NT:KT175741.1:VH00444:1:AAAMMTVHV:1:1510:
70929:50921/2
CTCTATACATCATTTTTCAATTTAGCAGAGTCCTGTGTTTTTATTAAACAGTCGCTACCCCCTATTCTGT
TACGCACACATAGATTAC
>family_nr:-300:family_nt:555408:genus_nr:-200:genus_nt:66526:species_
nr:-100:species_nt:66527:NR::NT:KT175741.1:VH00444:1:AAAMMTVHV:1:1612:
73144:45716/1
TATTATGCGGTTTCTTAAACGATGATATCATTGATGTATTCAAGCTCATATCTGAACGTTATTGTCTCGA
TTTAATTCTTCCAAAACGTTTGTCTATATACTGGATGAAAACG
>family_nr:-300:family_nt:555408:genus_nr:-200:genus_nt:66526:species_
nr:-100:species_nt:66527:NR::NT:KT175741.1:VH00444:1:AAAMMTVHV:1:1612:
73144:45716/2

CGTTTTCATCCAGTATATAGACAAACGTTTTGGAAGAATTAAATCGAGACAATAACGTTCAGATATGAGC
TTGAATACATCAATGATATCATCGTTTAAGAAACCGCATAATA
>family_nr:-300:family_nt:555408:genus_nr:-200:genus_nt:66526:species_nr:-100:species_nt:66527:NR::NT:KT175741.1:VH00444:1:AAAMMTVHV:1:2102:46540:33619/1
CTAGAATTATACGAACGTTGGCGAAACTCCTAATTTCTTTTTTTTTTAAAGAATGGTGCCATCGTTTTTC
TTAAGTGACAACTCGTCGATAGGGTGGCTCCTATCG
>family_nr:-300:family_nt:555408:genus_nr:-200:genus_nt:66526:species_nr:-100:species_nt:66527:NR::NT:KT175741.1:VH00444:1:AAAMMTVHV:1:2102:46540:33619/2
CGATAGGAGCCACCCTATCGACGAGTTGTCACTTAAGAAAAACGATGGCACCATTCTTTAAAAAAAAAAG
AAATTAGGAGTTTCGCCAACGTTCGTATAATTCTAG
>family_nr:-300:family_nt:555408:genus_nr:-200:genus_nt:66526:species_nr:-100:species_nt:66527:NR::NT:KT175741.1:VH00444:1:AAAMMTVHV:1:2103:15524:35966/1
CAACTGACATATTTTCTGAAAAAGTTCTAAAATTTGGTTATTTTTTT
>family_nr:-300:family_nt:555408:genus_nr:-200:genus_nt:66526:species_nr:-100:species_nt:66527:NR::NT:KT175741.1:VH00444:1:AAAMMTVHV:1:2103:15524:35966/2
AAAAAAATAACCAAATTTTAGAACTTTTTCAGAAAATATGTCAGTTG
>family_nr:-300:family_nt:555408:genus_nr:-200:genus_nt:66526:species_nr:-100:species_nt:66527:NR::NT:KT175741.1:VH00444:1:AAAMMTVHV:1:2113:32717:43728/1
CCATCAATATAGGTTCGAGTCCTATTACTCTAAGATCTCTTCGTTCAACCTGGTAGGACCTTGGATTTTC
GTTCCAAAGATGTGGGTTCAAATCCCACAGGGAGTAAAAAATTCCAAGACAAAGAAGCCTTTCGCTAGCA
TTCACTC
>family_nr:-300:family_nt:555408:genus_nr:-200:genus_nt:66526:species_nr:-100:species_nt:66527:NR::NT:KT175741.1:VH00444:1:AAAMMTVHV:1:2113:32717:43728/2
GTTTGTATTTTCTAATTTAATAACGAGTTTGATTCTGGCTCCGAGTGAATGCTAGCGAAAGGCTTCTTTG
TCTTGGAATTTTTTACTCCCTGTGGGATTTGAACCCACATCTTTGGAACGAAAATCCAAGGTCCTACCAG
GTTGAAC
>family_nr:-300:family_nt:555408:genus_nr:-200:genus_nt:66526:species_nr:-100:species_nt:66527:NR::NT:KT175741.1:VH00444:1:AAAMMTVHV:1:2202:61480:53931/2
AGTAAAAAGAACGTTTGTTAAAAAAATTTTGGTTCTTGTTCCTTTGGGCGCTGAGAAT
>family_nr:-300:family_nt:555408:genus_nr:-200:genus_nt:66526:species_nr:-100:species_nt:66527:NR::NT:KT175741.1:VH00444:1:AAAMMTVHV:1:2203:54550:6339/1
GAACTATTAGGGACCGACTAACCCGATGAAAGTTACTTTACATCGGAATCCTTACATATTTGATGAGATT
GTTCTTCACCAACCTAAACGTTACTCGTGTCAACATTATCACACCATATACTTTTTCTATCGAAAACATA
TATATAT
>family_nr:-300:family_nt:555408:genus_nr:-200:genus_nt:66526:species_nr:-100:species_nt:66527:NR::NT:KT175741.1:VH00444:1:AAAMMTVHV:1:2203:54550:6339/2
CTTTGATTATAAAATTAAGTAAAGGCAAGGTAGTAGAACGACCATTAAAGATATATATATGTTTTCGATA
GAAAAAGTATATGGTGTGATAATGTTGACACGAGTAACGTTTAGGTTGGTGAAGAACAATCTCATCAAAT
ATGTAAG
>family_nr:-300:family_nt:555408:genus_nr:-200:genus_nt:66526:species_nr:-100:species_nt:66527:NR::NT:KT175741.1:VH00444:1:AAAMMTVHV:1:2209:

20996:5316/1
GTCACATGTTTCGAAGTGATTATTCCTTAATAAGTTCATGTTATATGTGTTACTGGTATCGACAATTATA
TAGGTAACTGGGGTGAAGTCGTAACAAGG
>family_nr:-300:family_nt:555408:genus_nr:-200:genus_nt:66526:species_
nr:-100:species_nt:66527:NR::NT:KT175741.1:VH00444:1:AAAMMTVHV:1:2209:
20996:5316/2
CCTTGTTACGACTTCACCCCAGTTACCTATATAATTGTCGATACCAGTAACACATATAACATGAACTTAT
TAAGGAATAATCACTTCGAAACATGTGAC
>family_nr:-300:family_nt:555408:genus_nr:-200:genus_nt:66526:species_
nr:-100:species_nt:66527:NR::NT:KT175741.1:VH00444:1:AAAMMTVHV:1:2213:
38871:37235/2
CCTGATGCACCCATGTTGTTTTAGGTTTAGGTTAAAGTAATGGTCAATTATTGTTATAATTTGTAATGAA
ATACAAAAGTGTAGTGTAACCAATGGATTTGAGCGGTTATGTATTTAAAGACGTATAGGTATGACGAAAC
TATTTAC
>family_nr:-300:family_nt:555408:genus_nr:-200:genus_nt:66526:species_
nr:-100:species_nt:66527:NR::NT:KT175741.1:VH00444:1:AAAMMTVHV:1:2302:
18686:48290/1
CCCTATAAATAAATTGGCGAACTCGACGAGATTGAATTCTAATTAACTTTTTAATTTTCGTGTCGTTCTA
TAGTTCACAATTCAGTCAA
>family_nr:-300:family_nt:555408:genus_nr:-200:genus_nt:66526:species_
nr:-100:species_nt:66527:NR::NT:KT175741.1:VH00444:1:AAAMMTVHV:1:2302:
18686:48290/2
TTGACTGAATTGTGAACTATAGAACGACACGAAAATTAAAAAGTTAATTAGAATTCAATCTCGTCGAGTT
CGCCAATTTATTTATAGGG
>family_nr:-300:family_nt:555408:genus_nr:-200:genus_nt:66526:species_
nr:-100:species_nt:66527:NR::NT:KT175741.1:VH00444:1:AAAMMTVHV:1:2304:
55364:40510/1
TTGCTTTTAAAAAGGCGTAGGTTGCAATGGTTTTGTTTATTGATTATTATTTTTTAAGTCTTTTGTTGTT
GTATTT
>family_nr:-300:family_nt:555408:genus_nr:-200:genus_nt:66526:species_
nr:-100:species_nt:66527:NR::NT:KT175741.1:VH00444:1:AAAMMTVHV:1:2304:
55364:40510/2
AAATACAACAACAAAAGACTTAAAAAATAATAATCAATAAACAAAACCATTGCAACCTACGCCTTTTTAA
AAGCAA
>family_nr:-300:family_nt:555408:genus_nr:-200:genus_nt:66526:species_
nr:-100:species_nt:66527:NR::NT:KT175741.1:VH00444:1:AAAMMTVHV:1:2401:
22795:9652/1
TAACCGGATTTACGGTTTCCCATCAACCACGGACGTGAGTTTTTCTCCCCATAATCAGATTTTCGGTCAC
CCATTAGCCAAGGGCGTGAA
>family_nr:-300:family_nt:555408:genus_nr:-200:genus_nt:66526:species_
nr:-100:species_nt:66527:NR::NT:KT175741.1:VH00444:1:AAAMMTVHV:1:2401:
22795:9652/2
TTCACGCCCTTGGCTAATGGGTGACCGAAAATCTGATTATGGGGAGAAAAACTCACGTCCGTGGTTGATG
GGAAACCGTAAATCCGGTTA
>family_nr:-300:family_nt:555408:genus_nr:-200:genus_nt:66526:species_
nr:-100:species_nt:66527:NR::NT:KT175741.1:VH00444:1:AAAMMTVHV:1:2403:
13062:20670/1
CAATAATTGACCATTACTTTAACCTAAACCTAAAACAACATGGGTGCATCAGGACTCGAACCTGAAACCG
ATGATTTAAGAGACCATCGCTCTACCAATTGAGCTATACACTATTATAGAACCCATACAATACGGNTAGA
GAGACAA

>family_nr:-300:family_nt:555408:genus_nr:-200:genus_nt:66526:species_
nr:-100:species_nt:66527:NR::NT:KT175741.1:VH00444:1:AAAMMTVHV:1:2403:
13062:20670/2
GTATTTAATGTGTTATAAATTTGTTTTTTTGTCTCAAATAGTCTGTATATAGGGGAGGGGGAATAGTAGG
TATGCTATTCCCCCTCCTTGGGAATGTTGGTCTGTTTTTTATGGGTGTTGTCTCTCTAGCCGTATTGTAT
GGGTTCT
>family_nr:-300:family_nt:555408:genus_nr:-200:genus_nt:66526:species_
nr:-100:species_nt:66527:NR::NT:KT175741.1:VH00444:1:AAAMMTVHV:1:2410:
38795:47798/1
GTTGCTTTGTATGAAGAAAATTGCTGGTTCGTTAAAATTGGAGTTAGCACAGTATCGTGAGATTTTAG
>family_nr:-300:family_nt:555408:genus_nr:-200:genus_nt:66526:species_
nr:-100:species_nt:66527:NR::NT:KT175741.1:VH00444:1:AAAMMTVHV:1:2410:
38795:47798/2
CTAAAATCTCACGATACTGTGCTAACTCCAATTTTAACGAACCAGCAATTTTCTTCATACAAAGCAAC
>family_nr:-300:family_nt:555408:genus_nr:-200:genus_nt:66526:species_
nr:-100:species_nt:66527:NR::NT:KT175741.1:VH00444:1:AAAMMTVHV:1:2503:
56898:14404/1
TTTATCGGCGACCTCGTTGTGTTGTTGTGTTATTTTGTAACGAAGTTTTTTTAAAATCTGCAAAGATGGT
GGGTTCGGGACTTGCCACGAGCG
>family_nr:-300:family_nt:555408:genus_nr:-200:genus_nt:66526:species_
nr:-100:species_nt:66527:NR::NT:KT175741.1:VH00444:1:AAAMMTVHV:1:2503:
56898:14404/2
CGCTCGTGGCAAGTCCCGAACCCACCATCTTTGCAGATTTTAAAAAAACTTCGTTACAAAATAACACAAC
AACACAACGAGGTCGCCGATAAA
>family_nr:-300:family_nt:555408:genus_nr:-200:genus_nt:66526:species_
nr:-100:species_nt:66527:NR::NT:KT175741.1:VH00444:1:AAAMMTVHV:1:2504:
39685:28470/1
CCGCCTGTACCCAAAACAGGTACGCTACCAAGTTACGCCACGCACTGAAGAGAATAACAAACAACGACAA
AGGGAACAGGACTTGAACCTATATTACCGACTTTGGAGGACGGCGTTTTACCGATTAAACTATCCCCAAT
CAACCTT
>family_nr:-300:family_nt:555408:genus_nr:-200:genus_nt:66526:species_
nr:-100:species_nt:66527:NR::NT:KT175741.1:VH00444:1:AAAMMTVHV:1:2504:
39685:28470/2
ATAAAGGTTGATTGGGGATAGTTTAATCGGTAAAACGCCGTCCTCCAAAGTCGGTAATATAGGTTCAAGT
CCTGTTCCCTTTGTCGTTGTTTGTTATTCTCTTCAGTGCGTGGCGTAACTTGGTAGCGTACCTGTTTTGG
GTACAGG
>family_nr:-300:family_nt:555408:genus_nr:-200:genus_nt:66526:species_
nr:-100:species_nt:66527:NR::NT:KT175741.1:VH00444:1:AAAMMTVHV:1:2509:
36504:43501/1
AAATGAAGCGAAAAACAGAATCACAGAAAGCGCTAAAAAAAAAAAAAATAAAATACGCAAT
>family_nr:-300:family_nt:555408:genus_nr:-200:genus_nt:66526:species_
nr:-100:species_nt:66527:NR::NT:KT175741.1:VH00444:1:AAAMMTVHV:1:2511:
42677:33240/1
ATGGTTGTTTGTTCTGACTTTGATATAGCTGTTTCGTTGGGTTTTTAGTCATCGAGGTTATTGTTCCTGA
TTTAA
>family_nr:-300:family_nt:555408:genus_nr:-200:genus_nt:66526:species_
nr:-100:species_nt:66527:NR::NT:KT175741.1:VH00444:1:AAAMMTVHV:1:2511:
42677:33240/2
TTAAATCAGGAACAATAACCTCGATGACTAAAAACCCAACGAAACAGCTATATCAAAGTCAGAACAAACA
ACCAT

>family_nr:-300:family_nt:555408:genus_nr:-200:genus_nt:66526:species_
nr:-100:species_nt:66527:NR::NT:KT175741.1:VH00444:1:AAAMMTVHV:1:2514:
29403:40945/1
AAGATCTCTTCGTTCAACCTGGTAGGACCTTGGATTTTCGTTCCAAAGATGTGGGTTCAAATCCCACAGG
GAATAAAAAATTCCAAG
>family_nr:-300:family_nt:555408:genus_nr:-200:genus_nt:66526:species_
nr:-100:species_nt:66527:NR::NT:KT175741.1:VH00444:1:AAAMMTVHV:1:2514:
29403:40945/2
CTTGGAATTTTTATTCCCTGTGGGATTTGAACCCACATCTTTGGAACGAAAATCCAAGGTCCTACCAGG
TTGAACGAAGAGATCTT
>family_nr:-300:family_nt:555408:genus_nr:-200:genus_nt:66526:species_
nr:-100:species_nt:66527:NR::NT:KT175741.1:VH00444:1:AAAMMTVHV:1:2601:
41295:17698/1
TAACAGGACTAAGATTTTTACTTAAGCTAAGGTTGCTTTGATTATAAAATTAAGTAAAGGCAAGGTAGTA
GAACGACCATTAAAGATATATATATGTTTTCGATAGAAAAAGTATAT
>family_nr:-300:family_nt:555408:genus_nr:-200:genus_nt:66526:species_
nr:-100:species_nt:66527:NR::NT:KT175741.1:VH00444:1:AAAMMTVHV:1:2601:
41295:17698/2
ATATACTTTTTCTATCGAAAACATATATATATCTTTAATGGTCGTTCTACTACCTTGCCTTTACTTAATT
TTATAATCAAAGCAACCTTAGCTTAAGTAAAAATCTTAGTCCTGTTA
>family_nr:-300:family_nt:555408:genus_nr:-200:genus_nt:66526:species_
nr:-100:species_nt:66527:NR::NT:KT175741.1:VH00444:1:AAAMMTVHV:1:2603:
56519:37954/2
CTCCTGTTTTAAATATTTTATTTCGCAATTCTTATTTGAAAAATAACACACTAG
>family_nr:-300:family_nt:555408:genus_nr:-200:genus_nt:66526:species_
nr:-100:species_nt:66527:NR::NT:KT175741.1:VH00444:1:AAAMMTVHV:1:2607:
25256:50429/1
CAGGTATTTTGATAGAGTATATTAAGATGATTAAAAGAACAGCACTGAAGGAACTCGGCAAATTGACTCC
GTAACTTCGGGAAAAGGAGTGCTTGCATAGAGTAATCTATGTGTGCGTA
>family_nr:-300:family_nt:555408:genus_nr:-200:genus_nt:66526:species_
nr:-100:species_nt:66527:NR::NT:KT175741.1:VH00444:1:AAAMMTVHV:1:2607:
25256:50429/2
TACGCACACATAGAGTACTCTATGCAAGCACTCCTTTTCCCGAAGTTACGGAGTCAATTTGCCGAGTTCC
TTCAGTGCTGTTCTTTTAATCATCTTAATATACTCTATCAAAATACCTG
>family_nr:-300:family_nt:555408:genus_nr:-200:genus_nt:66526:species_
nr:-100:species_nt:66527:NR::NT:KT175741.1:VH00444:1:AAAMMTVHV:1:2609:
59549:43974/1
AATTGGTAAGGCGTTGGATTTTGACTCCATCAATATAGGTTCGAGTCCTATTACTCTAAGATCTCTTCGT
TCAACCTGGTAGGACCTTGGATTTTCGTTCCAAAGATGTGGGTTCAAATCCCACAGGGAGTAAAAAATTC
CAAGACA
>family_nr:-300:family_nt:555408:genus_nr:-200:genus_nt:66526:species_
nr:-100:species_nt:66527:NR::NT:KT175741.1:VH00444:1:AAAMMTVHV:1:2609:
59549:43974/2
TTCTTTGTCTTGGAATTTTTTACTCCCTGTGGGATTTGAACCCACATCTTTGGAACGAAAATCCAAGGTC
CTACCAGGTTGAACGAAGAGATCTTAGAGTAATAGGACTCGAACCTATATTGATGGAGTCAAAATCCAAC
GCCTTAC
>family_nr:-300:family_nt:555408:genus_nr:-200:genus_nt:66526:species_
nr:-100:species_nt:66527:NR::NT:MT270694.1:VH00444:1:AAAMMTVHV:1:1503:
36599:6263/2
AACAATAATCGGGTTGTAAGCTAATTGTTGTTACACTAACATGGAAGTTTTTACCTAGTTTATAACCGTG

TAACCAAGAAAATTAATTTGAAAAGTATGTCATCTTATGGCGTTG
>family_nr:-300:family_nt:555408:genus_nr:-200:genus_nt:66526:species_nr:77133:species_nt:66527:NR:AOE10192.1:NT:KF896589.1:VH00444:1:AAAMMTVHV:1:2112:12191:19307/2
TCAGGGCATGGACTACCAGGGTCTCTAATCCTGTTCGCTCCCCATGCTTTCGTACCTCAGCGTCAGTATAAAAATGGAAAG
>family_nr:-300:family_nt:555408:genus_nr:-200:genus_nt:66526:species_nr:77133:species_nt:66527:NR:AOE10192.1:NT:KP888565.1:VH00444:1:AAAMMTVHV:1:2606:16205:6585/1
TCAGGGCATGGACTACCAGGGTCTCTAATCCTGTTCGCTCCCCATGCTTTCGTACCTCAGCGTCAGTATAAAAATGGAAAG
>family_nr:-300:family_nt:555408:genus_nr:-200:genus_nt:66526:species_nr:77133:species_nt:66527:NR:AOE10192.1:NT:KR908790.1:VH00444:1:AAAMMTVHV:1:2606:16205:6585/2
CTTTCCATTTTTATACTGACGCTGAGGTACGAAAGCATGGGGAGCGAACAGGATTAGAGACCCTGGTAGTCCATGCCCTGA
>family_nr:-300:family_nt:555408:genus_nr:-200:genus_nt:66526:species_nr:77133:species_nt:66527:NR:AOE10192.1:NT:KT175740.1:VH00444:1:AAAMMTVHV:1:2112:12191:19307/1
CTTTCCATTTTTATACTGACGCTGAGGTACGAAAGCATGGGGAGCGAACAGGATTAGAGACCCTGGTAGTCCATGCCCTGA
>family_nr:119060:family_nt:555408:genus_nr:32008:genus_nt:66526:species_nr:87883:species_nt:66527:NR:BAG46932.1:NT:KT175741.1:VH00444:1:AAAMMTVHV:1:1303:40140:53534/1
TGTGACTATACAGAGATTTGTTCCGCAAAACTCTTATTTCATCGACGCCTCATCAAAGTTACTTGCGTAACGTGAGTTATAAGCTTAAGAGTTATTCTACATAAACACAACAAACGTACAATCCCGTGTTATAGTAAAGGTGTACAG
>family_nr:119060:family_nt:555408:genus_nr:32008:genus_nt:66526:species_nr:87883:species_nt:66527:NR:BAG46932.1:NT:KT175741.1:VH00444:1:AAAMMTVHV:1:1303:40140:53534/2
TCCGACCTGCATGAATGATGTAACGACTTCCCCACTGTCTCCAGTGTTGCTTTAGTGAAACTGAATTTGTTGTGAAGATGCAATATTAATAGTAAGTAGACGGAAAGACCCTGTACACCTTTACTATAACACGGGATTGTACGTTTG
>family_nr:119060:family_nt:555408:genus_nr:32008:genus_nt:66526:species_nr:87883:species_nt:66527:NR:BAG46932.1:NT:KT175741.1:VH00444:1:AAAMMTVHV:1:1609:20409:47173/1
CGTTGAGCAATAGCCTTTCCACACAGCACTATTGGATCATTATGATCGACTTTCGTCTCTGTTCGAAGTGTAATTCTTACAGTTAAGCATAATTTTACCATTAAATTCAAAATATACCTTTATACATCTTCGTTACATTTTAGAAGA
>family_nr:119060:family_nt:555408:genus_nr:32008:genus_nt:66526:species_nr:87883:species_nt:66527:NR:BAG46932.1:NT:KT175741.1:VH00444:1:AAAMMTVHV:1:1609:20409:47173/2
ACTTTGATGAGGCGTCGATGAAATAAGAGTTTTGCGGAACAAATCTCTGTATAGTCACATTAAAAATCCAGTGTGGTTAGTTTAACTGGGGTGGTTGTCTTCTAAAATGTAACGAAGATGTATAAAGGTATATTTTGAATTTAATGG
>family_nr:119060:family_nt:555408:genus_nr:32008:genus_nt:66526:species_nr:87883:species_nt:66527:NR:BAG46932.1:NT:KT175741.1:VH00444:1:AAAMMTVHV:1:2413:34042:42668/1
TGTAAGAATTACACTTCGAACAGAGACGAAAGTCGATCATAATGATCCAATAGTGCTGTGTGGAAAGGCTATTGCTCAACGGATAAAAGGTACGTCAGGGATAACAGGCTAATAAATCTTTAGAGTCCATATTTCCAGGT

TTGTTTG
>family_nr:119060:family_nt:555408:genus_nr:32008:genus_nt:66526:speci
es_nr:87883:species_nt:66527:NR:BAG46932.1:NT:KT175741.1:VH00444:1:AAA
MMTVHV:1:2413:34042:42668/2
TCCTGCAGCTCCAGGATGTGATGAGTCGACATCGAAGTGCCAAACAAACCTGGAAATATGGACTCTAAAG
ATTTATTAGCCTGTTATCCCTGACGTACCTTTTATCCGTTGAGCAATAGCCTTTCCACACAGCACTATTG
GATCATT
>family_nr:119060:family_nt:555408:genus_nr:32008:genus_nt:66526:speci
es_nr:87883:species_nt:66527:NR:BAG46932.1:NT:KT175741.1:VH00444:1:AAA
MMTVHV:1:2509:10714:30855/1
TAACTCTTAAGCTTATAACTCACGTTACGCAAGTAACTTTGATGAGGCGTCGATGAAATAAGAGTTTTGC
GGAACAAATCTCTGTATAGTCACATTAAAAATCCAGTGTGGTTAGTTTAACTG
>family_nr:119060:family_nt:555408:genus_nr:32008:genus_nt:66526:speci
es_nr:87883:species_nt:66527:NR:BAG46932.1:NT:KT175741.1:VH00444:1:AAA
MMTVHV:1:2509:10714:30855/2
CAGTTAAACTAACCACACTGGATTTTTAATGTGACTATACAGAGATTTGTTCCGCAAAACTCTTATTTCA
TCGACGCCTCATCAAAGTTACTTGCGTAACGTGAGTTATAAGCTTAAGAGTTN
>family_nr:31979:family_nt:555408:genus_nr:1485:genus_nt:66526:species
_nr:1354301:species_nt:66527:NR:EQB88155.1:NT:KT175741.1:VH00444:1:AAA
MMTVHV:1:1306:27226:9841/1
CTTCTAAACTGGAGGTACCGCCATCCCCTTTTTAACTCTAGTCATATAGTATTATAAAAAGATATAGTTT
TTTTTTTATAACTTAAATAACCGCCTACGTACCCTTTACACCCAGTCAATACGAATAACACTTGCCCTCT
CCGTTTT
>family_nr:31979:family_nt:555408:genus_nr:1485:genus_nt:66526:species
_nr:1354301:species_nt:66527:NR:EQB88155.1:NT:KT175741.1:VH00444:1:AAA
MMTVHV:1:1306:27226:9841/2
CCCTGACTAACTTCGTGCCAGCAGTCGCGGTAAAACGGAGAGGGCAAGTGTTATTCGTATTGACTGGGTG
TAAAGGGTACGTAGGCGGTTATTTAAGTTATAAAAAAAAACTATATCTTTTTATAATACTATATGACTA
GAGTTAA
>family_nr:31979:family_nt:555408:genus_nr:1485:genus_nt:66526:species
_nr:1354301:species_nt:66527:NR:EQB88155.1:NT:KT175741.1:VH00444:1:AAA
MMTVHV:1:2311:51728:24419/1
GGTCCCCCCAATCTCAAACGAATGTCACTTCTAAACTGGAGGTACCGCCATCCCCTTTTTAACTCTAGTC
ATATAGTATTATAAAAAGATATAGTTTTTTTTTTATAACTTAAATAACCGCCTACGTACCCTTTACACCC
AGTCAAT
>family_nr:31979:family_nt:555408:genus_nr:1485:genus_nt:66526:species
_nr:1354301:species_nt:66527:NR:EQB88155.1:NT:KT175741.1:VH00444:1:AAA
MMTVHV:1:2311:51728:24419/2
CGCATGTATGAAGAAGACCACTAAACGTTGTAAAGTACTAAGCTCGGGGATAATAATGACGGTACCCGAG
TAAAAAGCCCTGACTAACTTCGTGCCAGCAGTCGCGGTAAAACGGAGAGGACAAGTGTTATTCGTATTGA
CTGGGTG
>family_nr:31979:family_nt:555408:genus_nr:1485:genus_nt:66526:species
_nr:1354301:species_nt:66527:NR:EQB88155.1:NT:KT175741.1:VH00444:1:AAA
MMTVHV:1:2604:31713:45413/1
CCTTTTTAACTCTAGTCATATAGTATTATAAAAAGATATAGTTTTTTTTTTATAACTTAAATAACCGCCT
ACGTACCCTTTACACCCAGT
>family_nr:31979:family_nt:555408:genus_nr:1485:genus_nt:66526:species
_nr:1354301:species_nt:66527:NR:EQB88155.1:NT:KT175741.1:VH00444:1:AAA
MMTVHV:1:2604:31713:45413/2
ACTGGGTGTAAAGGGTACGTAGGCGGTTATTTAAGTTATAAAAAAAAACTATATCTTTTTATAATACTA

TATGACTAGAGTTAAAAAGG
>family_nr:31979:family_nt:555408:genus_nr:1485:genus_nt:66526:species_nr:1354301:species_nt:66527:NR:EQB88155.1:NT:KT175741.1:VH00444:1:AAAMMTVHV:1:2610:65703:13779/1
TATGACTAGAGTTAAAAAGGGGATGGCGGTACCTCCAGTTTAGAAGTGACATTCGTTTGAG
>family_nr:31979:family_nt:555408:genus_nr:1485:genus_nt:66526:species_nr:1354301:species_nt:66527:NR:EQB88155.1:NT:KT175741.1:VH00444:1:AAAMMTVHV:1:2610:65703:13779/2
CTCAAACGAATGTCACTTCTAAACTGGAGGTACCGCCATCCCCTTTTTAACTCTAGTCATA
>family_nr:506:family_nt:555408:genus_nr:152267:genus_nt:66526:species_nr:516702:species_nt:66527:NR:GGX37646.1:NT:KT175741.1:VH00444:1:AAAMMTVHV:1:2508:17171:12132/1
CCAATGTTGGTTAAACTAAAAATAATTTCAAATTGTTAGTTACCAAGCAGAATATAGGCGCAAAGGTCTATTTTTTCAAAAGGGAAACAGCCCAGACCTCCGGCTAAAGTCCATAAAAAAATTTGAGTGTAAAAAAAGAATTTTGGA
>family_nr:506:family_nt:555408:genus_nr:152267:genus_nt:66526:species_nr:516702:species_nt:66527:NR:GGX37646.1:NT:KT175741.1:VH00444:1:AAAMMTVHV:1:2508:17171:12132/2
CCAACTTGCCAATTCTAATCATCCAAAATTCTTTTTTTACACTCAAATTTTTTTATGGACTTTAGCCGGAGGTCTGGGCTGTTTCCCTTTTGAAAAAATAGACATTTGCGCCTATATTCTGCTTGGTAACTAACAATTTCAAATTAT
>family_nr:506:family_nt:555408:genus_nr:152267:genus_nt:66526:species_nr:516702:species_nt:66527:NR:GGX37646.1:NT:KT175741.1:VH00444:1:AAAMMTVHV:2:1102:62294:9463/1
GTCTATTTTTTCAAAAGGGAAACAGCCCAGACCTCCGGCTAAAGTCCATAAAAAAATTTGAGTGTAAAAAAAGAATTTTGGATGATTAGAATTGGCAAGTTGGCTTAGAAGCAGCCATCCTTTAAAGAAAGCGTAACAGCTCGTCAA
>family_nr:506:family_nt:555408:genus_nr:152267:genus_nt:66526:species_nr:516702:species_nt:66527:NR:GGX37646.1:NT:KT175741.1:VH00444:1:AAAMMTVHV:2:1102:62294:9463/2
TTCTAGGATTGACGAGCTGTTACGCTTTCTTTAAAGGATGGCTGCTTCTAAGCCAACTTGCCAATTCTAATCATCCAAAATTCTTTTTTTACACTCAAATTTTTTTATGGACTTTAGCCGGAGGTCTGGGCTGTTTCCCTTTTGAAA
>family_nr:543314:family_nt:555408:genus_nr:2060094:genus_nt:66526:species_nr:433296:species_nt:66527:NR:QIB67788.1:NT:KT175741.1:VH00444:1:AAAMMTVHV:1:1210:20276:21030/1
AAACATGTGACTTAATATCTTCAAACCAAACAAGCTTCCATGGCGTGACGGGCGGTGTGTACTAAGATCGGTCTTAATATTCACCGCAACATTCTTTTTTGCGATTACTAGCGATTCCGGCTTCATATTTTTATTTCAAAAAATAAT
>family_nr:543314:family_nt:555408:genus_nr:2060094:genus_nt:66526:species_nr:433296:species_nt:66527:NR:QIB67788.1:NT:KT175741.1:VH00444:1:AAAMMTVHV:1:1210:20276:21030/2
GAACGGGCGCAACCCTTGTTCCTAGTTAGATTAGGAATGAAAACAGTGAAAACTGCTTTTGAGGATGAGGAGTATGTCAAGTCAATATGACCTTTATAGATTGGGCTACACACGTACTACAACGGTAGCTACAATGAGAAACAATGC
>family_nr:543314:family_nt:555408:genus_nr:2060094:genus_nt:66526:species_nr:433296:species_nt:66527:NR:QIB67788.1:NT:KT175741.1:VH00444:1:AAAMMTVHV:1:1312:13194:29738/1
TTTGAGGATGAGGAGTATGTCAAGTCAATATGACCTTTATAGATTGGGCTACACACGTACTACAACGGTAGCTACAATGAGAAACAATGCCGTGAGGCGGAGTAAATCTCTAAAACCTACCCTAGTTCGGATTATTTTTT

GAAATAA
>family_nr:543314:family_nt:555408:genus_nr:2060094:genus_nt:66526:spe
cies_nr:433296:species_nt:66527:NR:QIB67788.1:NT:KT175741.1:VH00444:1:
AAAMMTVHV:1:1312:13194:29738/2
CATATTTTTATTTCAAAAAATAATCCGAACTAGGGTAGGTTTTAGAGATTTACTCCGCCTCACGGCATTG
TTTCTCATTGTAGCTACCGTTGTAGTACGTGTGTAGCCCAATCTATAAAGGTCATATTGACTTGACATAC
TCCTCAT
>family_nr:543314:family_nt:555408:genus_nr:2060094:genus_nt:66526:spe
cies_nr:433296:species_nt:66527:NR:QIB67788.1:NT:KT175741.1:VH00444:1:
AAAMMTVHV:1:2401:38625:6698/1
TTTGAAGATGAGGAGTATGTCAAGTCAATATGACCTTTATAGATTGGGCTACACACGTACTACAACGGTA
GCTACAATGAGAAACAATGCCGTGAGGCGGAGTAAATCTCTAAAACCTACCCTAGTTCGGATTATTTTTT
GAAATAA
>family_nr:543314:family_nt:555408:genus_nr:2060094:genus_nt:66526:spe
cies_nr:433296:species_nt:66527:NR:QIB67788.1:NT:KT175741.1:VH00444:1:
AAAMMTVHV:1:2401:38625:6698/2
CATATTTTTATTTCAAAAAATAATCCGAACTAGGGTAGGTTTTAGAGATTTACTCCGCCTCACGGCATTG
TTTCTCATTGTAGCTACCGTTGTAGTACGTGTGTAGCCCAATCTATAAAGGTCATATTGACTTGACATAC
TCCTCAT
>family_nr:543314:family_nt:555408:genus_nr:2060094:genus_nt:66526:spe
cies_nr:433296:species_nt:66527:NR:QIB67788.1:NT:KT175741.1:VH00444:1:
AAAMMTVHV:1:2407:40916:5733/1
GAACGGGCGCAACCCTTGTTCCTAGTTAGATTAGGAATGAAAACAGTGAAAACTGCTTTTGAGGATGAGG
AGTATGTCAAGTCAATATGACCTTTATAGATTGGGCTACACACGTACTACAACGGTAGCTACAATGAGAA
ACAATGC
>family_nr:543314:family_nt:555408:genus_nr:2060094:genus_nt:66526:spe
cies_nr:433296:species_nt:66527:NR:QIB67788.1:NT:KT175741.1:VH00444:1:
AAAMMTVHV:1:2407:40916:5733/2
AAAAATGTGACTTAATATCTTCAAACCAAACAAGCTTCCATGGCGTGACGGGCGGTGTGTACTAAGATCG
GTCTTAATATTCACCGCAACATTCTTTTTTGCGATTACTAGCGATTCCGGCTTCATATTTTTATTTCAAA
AAATAAT
>family_nr:555408:family_nt:555408:genus_nr:66526:genus_nt:66526:speci
es_nr:66527:species_nt:66527:NR:AKT25951.1:NT:KT030673.1:VH00444:1:AAA
MMTVHV:1:2414:39193:45318/1
TTCCTAAAATTCCGGAAAACGCTCCAAACAATAGATACAAAGTACCGATTGTTTTATGATTTGTTGACCA
AAACCATTTTGTAT
>family_nr:555408:family_nt:555408:genus_nr:66526:genus_nt:66526:speci
es_nr:66527:species_nt:66527:NR:AKT25951.1:NT:KT030673.1:VH00444:1:AAA
MMTVHV:1:2414:39193:45318/2
ATACAAAATGGTTTTGGTCAACAAATCATAAAACAATCGGTACTTTGTATCTATTGTTTGGAGCGTTTTC
CGGAATTTTAGGAA
>family_nr:555408:family_nt:555408:genus_nr:66526:genus_nt:66526:speci
es_nr:66527:species_nt:66527:NR:AKT25951.1:NT:KT175741.1:VH00444:1:AAA
MMTVHV:1:2403:18705:38352/1
ATGTAACATTTTTCCCAATGCATTTTTTAGGATTGGCTGGTATGCCCCGAAGAATTCCAGATTATCCAGA
TGTTTATGCAGTTTTTA
>family_nr:555408:family_nt:555408:genus_nr:66526:genus_nt:66526:speci
es_nr:66527:species_nt:66527:NR:AKT25951.1:NT:KT175741.1:VH00444:1:AAA
MMTVHV:1:2403:18705:38352/2
TAAAAACTGCATAAACATCTGGATAATCTGGAATTCTTCGGGGCATACCAGCCAATCCTAAAAAATGCAT

TGGGAAAAATGTTACAT
>family_nr:555408:family_nt:555408:genus_nr:66526:genus_nt:66526:speci
es_nr:66527:species_nt:66527:NR:AKT25952.1:NT:KT175741.1:VH00444:1:AAA
MMTVHV:1:2307:27585:30041/1
TTTAAAAAAACGGTTGTATACGCAACATAAACAATAATAAATAAAAAACACAGGACTTAAAA
>family_nr:555408:family_nt:555408:genus_nr:66526:genus_nt:66526:speci
es_nr:66527:species_nt:66527:NR:AKT25952.1:NT:KT175741.1:VH00444:1:AAA
MMTVHV:1:2307:27585:30041/2
TTTTAAGTCCTGTGTTTTTTATTTATTATTGTTTATGTTGCGTATACAACCGTTTTTTTAAA
>family_nr:555408:family_nt:555408:genus_nr:66526:genus_nt:66526:speci
es_nr:66527:species_nt:66527:NR:AKT25955.1:NT:KT175741.1:VH00444:1:AAA
MMTVHV:1:2112:38511:22980/1
ATTAAAACAAAAAATGTACAGAACATATTTTGAAAAATAAAACCGCCCAACACCAACACTTCACCAACAA
AATTACTCGTCCCGGGAAAACTAAAATTCGAAAGAATTAG
>family_nr:555408:family_nt:555408:genus_nr:66526:genus_nt:66526:speci
es_nr:66527:species_nt:66527:NR:AKT25955.1:NT:KT175741.1:VH00444:1:AAA
MMTVHV:1:2112:38511:22980/2
CTAATTCTTTCGAATTTTAGTTTTCCCGGGACGAGTAATTTTGTTGGTGAAGTGTTGGTGTTGGGCGGTT
TTATTTTTCAAAATATGTTCTGTACATTTTTTGTTTTAAT
>family_nr:555408:family_nt:555408:genus_nr:66526:genus_nt:66526:speci
es_nr:66527:species_nt:66527:NR:AKT25955.1:NT:KT175741.1:VH00444:1:AAA
MMTVHV:1:2505:71781:43936/1
GCTCATGTAGAAGCACCTACTATAGGTTCGGTCATTTTGGCCGGTGTGTTGTTGAAAATGGGGGGTTATG
GATTTTTGCGTTTTGTTGTACCGCTATTACCCATAGGAAATTATTTT
>family_nr:555408:family_nt:555408:genus_nr:66526:genus_nt:66526:speci
es_nr:66527:species_nt:66527:NR:AKT25955.1:NT:KT175741.1:VH00444:1:AAA
MMTVHV:1:2505:71781:43936/2
AAAATAATTTCCTATGGGTAATAGCGGTACAACAAAACGCAAAAATCCATAACCCCCCATTTTCAACAAC
ACACCGGCCAAAATGACCGAACCTATAGTAGGTGCTTCTACATGAGC
>family_nr:555408:family_nt:555408:genus_nr:66526:genus_nt:66526:speci
es_nr:66527:species_nt:66527:NR:AKT25960.1:NT:KP888565.1:VH00444:1:AAA
MMTVHV:1:2209:9824:12321/1
CGTTTCCGGTTTTACGGTTACTTTGAGTCATGCATTTATTCGTCAAAATCGTATAGAGGAGTCTTTATT
>family_nr:555408:family_nt:555408:genus_nr:66526:genus_nt:66526:speci
es_nr:66527:species_nt:66527:NR:AKT25960.1:NT:KP888565.1:VH00444:1:AAA
MMTVHV:1:2511:73580:12757/2
GGAAACGACTAAAATTATTGTATTTGCTAATGGAACACCTAGCGGATTAATCGATGGAATTCCTACAGGC
GGTCATTGACCACCAACTCAAAT
>family_nr:555408:family_nt:555408:genus_nr:66526:genus_nt:66526:speci
es_nr:66527:species_nt:66527:NR:AKT25960.1:NT:KT030671.1:VH00444:1:AAA
MMTVHV:1:2209:9824:12321/2
AATAAAGACTCCTCTATACGATTTTGACGAATAAATGCATGACTCAAAGTAACCGTAAAACCGGAAACG
>family_nr:555408:family_nt:555408:genus_nr:66526:genus_nt:66526:speci
es_nr:66527:species_nt:66527:NR:AKT25960.1:NT:KT175740.1:VH00444:1:AAA
MMTVHV:1:2511:73580:12757/1
ATTTGAGTTGGTGGTCAATGACCGCCTGTAGGAATTCCATCGATTAATCCGCTAGGTGTTCCATTAGCAA
ATACAATAATTTTAGTCGTTTCC
>family_nr:555408:family_nt:555408:genus_nr:66526:genus_nt:66526:speci
es_nr:66527:species_nt:66527:NR:AKT25960.1:NT:KT175741.1:VH00444:1:AAA
MMTVHV:1:1101:12267:26728/1

TTTTTGGTTTTTTTATTTTTTTTTTTTGGTTTTGTGTTTCTTGTTGCGGTTTTCGTATTATGGTGCCGTGA
CATTGTTCGTGAAGCAGTTATTTTTCGTGTACATTCAAATAAA
>family_nr:555408:family_nt:555408:genus_nr:66526:genus_nt:66526:speci
es_nr:66527:species_nt:66527:NR:AKT25960.1:NT:KT175741.1:VH00444:1:AAA
MMTVHV:1:1101:12267:26728/2
TTTATTTGAATGTACACGAAAAATAACTGCTTCACGAACAATGTCACGGCACCATAATACGAAAACCGCA
ACAAGAAACACAAAACCAAAAAAAAAAATAAAAAAACCAAAAA
>family_nr:555408:family_nt:555408:genus_nr:66526:genus_nt:66526:speci
es_nr:66527:species_nt:66527:NR:AKT25960.1:NT:KT175741.1:VH00444:1:AAA
MMTVHV:1:2403:47695:44390/1
CTTCAAAACCAAAATGACGATTTGCGGAAAAATGGCCGGCCAGCAACCTATAAAAACAAAC
>family_nr:555408:family_nt:555408:genus_nr:66526:genus_nt:66526:speci
es_nr:66527:species_nt:66527:NR:AKT25960.1:NT:KT175741.1:VH00444:1:AAA
MMTVHV:1:2403:47695:44390/2
GTTTGTTTTTATAGGTTGCTGGCCGGCCATTTTTCCGCAAATCGTCATTTTGGTTTTGAAG
>family_nr:555408:family_nt:555408:genus_nr:66526:genus_nt:66526:speci
es_nr:66527:species_nt:66527:NR:AKT25961.1:NT:KT175741.1:VH00444:1:AAA
MMTVHV:1:1614:38776:3291/1
ATATACGATTTGCCCTTTAACCGGCGTAGTTTAAAAATATACCGTTTCCATACACGAAGCCTAAAT
>family_nr:555408:family_nt:555408:genus_nr:66526:genus_nt:66526:speci
es_nr:66527:species_nt:66527:NR:AKT25961.1:NT:KT175741.1:VH00444:1:AAA
MMTVHV:1:1614:38776:3291/2
ATTTAGGCTTCGTGTATGGAAACGGTATATTTTTAAACTACGCCGGTTAAAGGGCAAATCGTATAT
>family_nr:555408:family_nt:555408:genus_nr:66526:genus_nt:66526:speci
es_nr:66527:species_nt:66527:NR:AKT25961.1:NT:KT175741.1:VH00444:1:AAA
MMTVHV:1:2103:48736:28508/1
CCAAAAACTTTTTTAAACGACGAACACGAGGGTACGCTAATCTATTAAAAAACCTATTTAAAGTCCGAGC
AAAAACATCTTTACGTGCAATATACGATTTGCCCTTTAACCGGCGTAGTTTAAAAAAGTTTTTGG
>family_nr:555408:family_nt:555408:genus_nr:66526:genus_nt:66526:speci
es_nr:66527:species_nt:66527:NR:AKT25961.1:NT:KT175741.1:VH00444:1:AAA
MMTVHV:1:2103:48736:28508/2
CCAAAAACTTTTTTAAACTACGCCGGTTAAAGGGCAAATCGTATATTGCACGTAAAGATGTTTTTGCTCG
GACTTTAAATAGGTTTTTTAATAGATTAGCGTACCCTCGTGTTCGTCGTTTAAAAAAGTTTTTGG
>family_nr:555408:family_nt:555408:genus_nr:66526:genus_nt:66526:speci
es_nr:66527:species_nt:66527:NR:AKT25961.1:NT:KT175741.1:VH00444:1:AAA
MMTVHV:1:2103:53508:47097/1
AATATACGATTTGCCCTTTAACCGGCGTAGTTTAAAAATATACCGTTTCCATACACGAAGCCTAAATACG
CGCGACCGTACAAGGTAAGCCTTCAACGCAAAAAGATAGGAAATAGTATATTGCACGTAAAGATGTTTTT
GCTCGGA
>family_nr:555408:family_nt:555408:genus_nr:66526:genus_nt:66526:speci
es_nr:66527:species_nt:66527:NR:AKT25961.1:NT:KT175741.1:VH00444:1:AAA
MMTVHV:1:2414:67539:38446/1
CTCAAAGAATTACGTAAAAAAGAATAGGTATAATTCAAATTATGGATATAAACCCGATTCTGCACGCCTA
AAAGAAAATTTGAAACACGAAAATCTCAGTGAGTAATATCATAACCTACGAAAGAACCGGGCATTAACAA
TTGTAAC
>family_nr:555408:family_nt:555408:genus_nr:66526:genus_nt:66526:speci
es_nr:66527:species_nt:66527:NR:AKT25961.1:NT:KT175741.1:VH00444:1:AAA
MMTVHV:1:2414:67539:38446/2
CTCAAAGAATTACGTAAAAAAATAATTGGGTTAATGATTTTTTTCAATAAAGGAACTGTTTTAAATTTGAC
TTTGTTACAATTGTTAATGCCCGGTTCTTTCGTAGGTTATGATATTTCTCACTGAGATTTTCGTGTTTCA

AATTTTC
>family_nr:555408:family_nt:555408:genus_nr:66526:genus_nt:66526:species_nr:66527:species_nt:66527:NR:AKT25961.1:NT:KT175741.1:VH00444:1:AAAMMTVHV:1:2514:67994:32502/1
ACTGTTTTAAATTTGACTTTGTTACAATTGTTAATGCCCGGTTCTTTCGTAGGTTATGATATTTCTCACTGAGATTTTCGTGTT
>family_nr:555408:family_nt:555408:genus_nr:66526:genus_nt:66526:species_nr:66527:species_nt:66527:NR:AKT25961.1:NT:KT175741.1:VH00444:1:AAAMMTVHV:1:2514:67994:32502/2
AACACGAAAATCTCAGTGAGAAATATCATAACCTACGAAAGAACCGGGCATTAACAATTGTAACAAAGTCAAATTTAAAACAGT
>family_nr:555408:family_nt:555408:genus_nr:66526:genus_nt:66526:species_nr:66527:species_nt:66527:NR:AKT25962.1:NT:KP888565.1:VH00444:1:AAAMMTVHV:1:1314:15372:37291/1
CCTTCTTTCGGTATAAAAATGGATGCGGTCCCTGGTCGTTTAAACCAAACGAGTGCTTTTGTAATGGAATGTGGTCGTTATTATGGTCAGTGTAGCGAACTTTGTGGCGTAAATCATGCTTTTATGCCTATAGAAATTTGTGTAGAG
>family_nr:555408:family_nt:555408:genus_nr:66526:genus_nt:66526:species_nr:66527:species_nt:66527:NR:AKT25962.1:NT:KP888565.1:VH00444:1:AAAMMTVHV:1:1314:15372:37291/2
CTACACAAATTTCTATAGGCATAAAAGCATGATTTACGCCACAAAGTTCGCTACACTGACCATAATAACGACCACATTCCATTACAAAAGCACTCGTTTGGTTTAAACGACCAGGGACCGCATCCATTTTTATACCGAAAGAAGGAG
>family_nr:555408:family_nt:555408:genus_nr:66526:genus_nt:66526:species_nr:66527:species_nt:66527:NR:AKT25962.1:NT:KT030673.1:VH00444:1:AAAMMTVHV:1:1206:27131:26558/1
ATACTCAAATCACTATCTGGAACCATATAACTATCAAAAGCTATAGACTCTCCCCCGACAAGTGGGCGCAAATCAGAA
>family_nr:555408:family_nt:555408:genus_nr:66526:genus_nt:66526:species_nr:66527:species_nt:66527:NR:AKT25962.1:NT:KT030673.1:VH00444:1:AAAMMTVHV:1:2109:10884:13609/2
CTGGTCGTTTAAACCAAACGAGTGCTTTTGTAATGGAATGTGGTCGTTATTATGGTCAGTGTAGCGAACTTTGTGGCGTAAATCATGCTTTTATGCCTATAGAAATTTGTGTAGTTGAACCAGCTGATTACTATAATTGGTTATTAC
>family_nr:555408:family_nt:555408:genus_nr:66526:genus_nt:66526:species_nr:66527:species_nt:66527:NR:AKT25962.1:NT:KT175738.1:VH00444:1:AAAMMTVHV:1:1403:66195:5222/1
CTATATACTGGATGAAAACGGATCAGATAGGTTTGTTCAATTTTGGGGGGTGTCGACAGCAAATTGTTTTAAATTTTTGCAAACTCCCTTGGCTTTTACTATTGACTGATTACAGTTTCGCCAAGAACGGGTTAATAAATTAAAGTG
>family_nr:555408:family_nt:555408:genus_nr:66526:genus_nt:66526:species_nr:66527:species_nt:66527:NR:AKT25962.1:NT:KT175740.1:VH00444:1:AAAMMTVHV:1:1206:27131:26558/2
TTCTGATTTGCGCCCACTTGTCGGGGGAGAGTCTATAGCTTTTGATAGTTATATGGTTCCAGATAGTGATTTGAGTAT
>family_nr:555408:family_nt:555408:genus_nr:66526:genus_nt:66526:species_nr:66527:species_nt:66527:NR:AKT25962.1:NT:KT175741.1:VH00444:1:AAAMMTVHV:1:1403:66195:5222/2
TATGTGTTACACGATATTGACGATTAGAGATTAAATAGGTTAACCACTTTAATTTATTAACCCGTTCTTGGCGAAACTGTAATCAGTCAATAGTAAAAGCCAAGGGAGTTTGCAAAAATTTAAAACAATTTGCTGTCGAC

ACCCCCC
>family_nr:555408:family_nt:555408:genus_nr:66526:genus_nt:66526:species_nr:66527:species_nt:66527:NR:AKT25962.1:NT:KT175741.1:VH00444:1:AAAMMTVHV:1:2109:10884:13609/1
CTTTTTAGTAATAACCAATTATAGTAATCAGCTGGTTCAACTACACAAATTTCTATAGGCATAAAAGCATGATTTACGCCACAAAGTTCGCTACACTGACCATAATAACGACCACATTCCATTACAAAAGCACTCGTTTGGTTTAAA
>family_nr:555408:family_nt:555408:genus_nr:66526:genus_nt:66526:species_nr:66527:species_nt:66527:NR:AKT25965.1:NT:KP888565.1:VH00444:1:AAAMMTVHV:1:1606:35860:12302/1
CGAAACCTCAACCGGTACTGAAAGAAGAACATTTAGATCACGACTAATCATCATTTTTAAATAATAAAGTGAATGAAATAAAAAAT
>family_nr:555408:family_nt:555408:genus_nr:66526:genus_nt:66526:species_nr:66527:species_nt:66527:NR:AKT25965.1:NT:KT030672.1:VH00444:1:AAAMMTVHV:1:1606:35860:12302/2
ATTTTTTATTTCATTCACTTTATTATTTAAAAATGATGATTAGTCGTGATCTAAATGTTCTTCTTTCAGTACCGGTTGAGGTTTCG
>family_nr:555408:family_nt:555408:genus_nr:66526:genus_nt:66526:species_nr:66527:species_nt:66527:NR:AKT25966.1:NT:KP888565.1:VH00444:1:AAAMMTVHV:1:1306:39231:47287/2
ATTACTGGTATTGACCAGCTAATTGATCTTTGTTCGGTTGATTTTATAGGTCATCGAAAACGTTTCGGTCTTTTTTATATTTTTTCCAGTACGAAATTTAAATACCGTATTTATGTCCGTAGTTTTCTCTGTGAAAGTTCGTCGTTT
>family_nr:555408:family_nt:555408:genus_nr:66526:genus_nt:66526:species_nr:66527:species_nt:66527:NR:AKT25966.1:NT:KT030671.1:VH00444:1:AAAMMTVHV:1:1306:39231:47287/1
ATGAAGAAAACGACGAACTTTCACAGAGAAAACTACGGACATAAATACGGTATTTAAATTTCGTACTGGAAAAAATATAAAAAAGACCGAAACGTTTTCGATGACCTATAAAATCAACCGAACAAAGATCAATTAGCTGGTCAATAC
>family_nr:555408:family_nt:555408:genus_nr:66526:genus_nt:66526:species_nr:66527:species_nt:66527:NR:AKT25966.1:NT:KT030671.1:VH00444:1:AAAMMTVHV:1:1513:34895:31366/2
TATTAAAAAGTGTTTCAAAATTTGTTAATTTGGTTACAACTAAAAGAACAGATCCTTCTGGTGTTTGGATTGATACTTCGCCAGAGTCTTTTGTCCCCTTGGTTTTGTTTTTACGTAAAC
>family_nr:555408:family_nt:555408:genus_nr:66526:genus_nt:66526:species_nr:66527:species_nt:66527:NR:AKT25966.1:NT:KT175738.1:VH00444:1:AAAMMTVHV:1:1513:34895:31366/1
GTTTACGTAAAAACAAAACCAAGGGGACAAAAGACTCTGGCGAAGTATCAATCCAAACACCAGAAGGATCTGTTCTTTTAGTTGTAACCAAATTAACAAATTTTGAAACACTTTTTAATA
>family_nr:555408:family_nt:555408:genus_nr:66526:genus_nt:66526:species_nr:66527:species_nt:66527:NR:AKT25966.1:NT:KT175739.1:VH00444:1:AAAMMTVHV:1:2611:30331:48574/2
GGTCATCGAAAACGTTTCGGTCTTTTTTATATTTTTTCCAGTACGAAATTTAAATACCGTATTTATGTCCGTAGTTTTCTCTGTGAAAGTTCGTCGTTTTCTTCATTGTCGTTTCTTTTCAAAAACTCTTTGTGGTTGGAACGGGAG
>family_nr:555408:family_nt:555408:genus_nr:66526:genus_nt:66526:species_nr:66527:species_nt:66527:NR:AKT25970.1:NT:KT175741.1:VH00444:1:AAAMMTVHV:1:1404:55099:29303/1
TGAAAAAAACTTATAAATTATAAAGTATAATGGCCGATTCTGTAATTTTTTATTATAGCTTTTGTTATTCCATTCGTCCAATTTTTGATTTGTCGTTTCCGCCTGTAGAATATTTTACTTATTTGTCTTATGGGCTTCCG

GATGCGT
>family_nr:555408:family_nt:555408:genus_nr:66526:genus_nt:66526:speci
es_nr:66527:species_nt:66527:NR:AKT25970.1:NT:KT175741.1:VH00444:1:AAA
MMTVHV:1:1404:55099:29303/2
TACGACCAACCCAGCAACCCGGATTCGTAAAGAAAAAACATGTCAGTAAAAAGAAAATGACTTGTATAGT
CACAATAATCCTCCAACGCATCCGGAAGCCCATAAGACAAATAAGTAAAATATTCTACAGGCGGAAACGA
CAAATCA
>family_nr:555408:family_nt:555408:genus_nr:66526:genus_nt:66526:speci
es_nr:66527:species_nt:66527:NR:AKT25970.1:NT:KT175741.1:VH00444:1:AAA
MMTVHV:1:2102:48907:19137/1
CTACAGGCGGAAACGACAAATCAAAAATTGGACGAATGGAATAACAAAAGCTATAATAAAAAATTACAGA
ATCGGCCATTATACTTTATAATTTATAAGTTTTTTTCATATTTATGAAAT
>family_nr:555408:family_nt:555408:genus_nr:66526:genus_nt:66526:speci
es_nr:66527:species_nt:66527:NR:AKT25970.1:NT:KT175741.1:VH00444:1:AAA
MMTVHV:1:2102:48907:19137/2
ATTTCATAAATATGAAAAAAACTTATAAATTATAAAGTATAATGGCCGATTCTGTAATTTTTTATTATAG
CTTTTGTTATTCCATTCGTCCAATTTTTGATTTGTCGTTTCCGCCTGTAG
>family_nr:555408:family_nt:555408:genus_nr:66526:genus_nt:66526:speci
es_nr:66527:species_nt:66527:NR:AKT25970.1:NT:KT175741.1:VH00444:1:AAA
MMTVHV:1:2302:60514:10485/1
ATTTCATAAATATGAAAAAAACTTATAAATTATAAAGTATAATGGCCGATTCTGTAATTTTTTATTATAG
CTTTTGTTATTCCATTCGTCCAATTTTTGATTTGTCGTTTCCGCCTGTAG
>family_nr:555408:family_nt:555408:genus_nr:66526:genus_nt:66526:speci
es_nr:66527:species_nt:66527:NR:AKT25970.1:NT:KT175741.1:VH00444:1:AAA
MMTVHV:1:2302:60514:10485/2
CTACAGGCGGAAACGACAAATCAAAAATTGGACGAATGGAATAACAAAAGCTATAATAAAAAATTACAGA
ATCGGCCATTATACTTTATAATTTATAAGTTTTTTTCATATTTATGAAAT
>family_nr:555408:family_nt:555408:genus_nr:66526:genus_nt:66526:speci
es_nr:66527:species_nt:66527:NR:AKT25972.1:NT:KT030671.1:VH00444:1:AAA
MMTVHV:1:2206:38644:45488/2
CTCATTTTTGATCTTGAAATCATGTTCCTGTTTCCATGGTCCGTTGCATTTCTGGAAATGGGTTTATTTA
GTTATTTCGTGATTTTTGTATTTATTGGTCTGCTGTTCGTC
>family_nr:555408:family_nt:555408:genus_nr:66526:genus_nt:66526:speci
es_nr:66527:species_nt:66527:NR:AKT25972.1:NT:KT175741.1:VH00444:1:AAA
MMTVHV:1:2206:38644:45488/1
GACGAACAGCAGACCAATAAATACAAAAATCACGAAATAACTAAATAAACCCATTTCCAGAAATGCAACG
GACCATGGAAACAGGAACATGATTTCAAGATCAAAAATGAG
>family_nr:555408:family_nt:555408:genus_nr:66526:genus_nt:66526:speci
es_nr:66527:species_nt:66527:NR:AKT25978.1:NT:KT175741.1:VH00444:1:AAA
MMTVHV:1:2507:51331:42535/1
TTAGAATAAGAAAAGGATTTCTCTCGTTTAAAAAGAAAAACAAGAAAGATGAAAAATACAACAACAAAAG
ACTTAAAAAATAATAATCAATAAACAAAACCATTGCAACCTAC
>family_nr:555408:family_nt:555408:genus_nr:66526:genus_nt:66526:speci
es_nr:66527:species_nt:66527:NR:AKT25978.1:NT:KT175741.1:VH00444:1:AAA
MMTVHV:1:2507:51331:42535/2
GTAGGTTGCAATGGTTTTGTTTATTGATTATTATTTTTTAAGTCTTTTGTTGTTGTATTTTTCATCTTTC
TTGTTTTTCTTTTTAAACGAGAGAAATCCTTTTCTTATTCTAA
>family_nr:555408:family_nt:555408:genus_nr:66526:genus_nt:66526:speci
es_nr:66527:species_nt:66527:NR:AKT25979.1:NT:KT175741.1:VH00444:1:AAA
MMTVHV:1:1605:56443:1492/1

```
CTTCGACAGGGCGTGGATAGTAACCGTAATTTTATTGAAGAGTCTGTGTTAAGTTCGATGTGTTTTTGCA
CTGCCGCTCGTTTAGCACGTGTTACTAG
>family_nr:555408:family_nt:555408:genus_nr:66526:genus_nt:66526:speci
es_nr:66527:species_nt:66527:NR:AKT25979.1:NT:KT175741.1:VH00444:1:AAA
MMTVHV:1:1605:56443:1492/2
CTAGTAACACGTGCTAAACGAGCGGCAGTGCAAAAACACATCGAACTTAACACAGACTCTTCAATAAAAT
TACGGTTACTATCCACGCCCTGTCGAAG
>family_nr:555408:family_nt:555408:genus_nr:66526:genus_nt:66526:speci
es_nr:66527:species_nt:66527:NR:AKT25980.1:NT:KT175739.1:VH00444:1:AAA
MMTVHV:1:2505:75398:18626/1
ATTAATAACAAAGAAACGTAACAATACATAAATGTTTTGCGAAAATAAAAAAAGCTTATTAGTAAGAAAC
AAACCATTTAAAAATCCAGCAACCTATAAAAACAAACACATAATC
>family_nr:555408:family_nt:555408:genus_nr:66526:genus_nt:66526:speci
es_nr:66527:species_nt:66527:NR:AKT25980.1:NT:KT175741.1:VH00444:1:AAA
MMTVHV:1:1409:13990:38181/1
TTTCAAATAGTTTTTGCAAATTTTTTAGACGGATCCCAAAAAAATTAATAACAAAGAAACGTAACAATAC
ATAAAT
>family_nr:555408:family_nt:555408:genus_nr:66526:genus_nt:66526:speci
es_nr:66527:species_nt:66527:NR:AKT25980.1:NT:KT175741.1:VH00444:1:AAA
MMTVHV:1:1409:13990:38181/2
ATTTATGTATTGTTACGTTTCTTTGTTATTAATTTTTTTGGGATCCGTCTAAAAAATTTGCAAAAACTAT
TTGAAA
>family_nr:555408:family_nt:555408:genus_nr:66526:genus_nt:66526:speci
es_nr:66527:species_nt:66527:NR:AKT25980.1:NT:KT175741.1:VH00444:1:AAA
MMTVHV:1:2505:75398:18626/2
GATTATGTGTTTGTTTTTATAGGTTGCTGGATTTTTAAATGGTTTGTTTCTTACTAATAAGCTTTTTTTA
TTTTCGCAAAACATTTATGTATTGTTACGTTTCTTTGTTATTAAT
>family_nr:555408:family_nt:555408:genus_nr:66526:genus_nt:66526:speci
es_nr:66527:species_nt:66527:NR:AKT93753.1:NT:KT175741.1:VH00444:1:AAA
MMTVHV:1:1102:71478:20121/1
GTGATGTAACACTAAAGAAAACAAGGGCTTTTTCAAAAGATTTAATTTTGTCTAAAATAATATCAAACAA
ATAGCCAGACATGCCGGAAAAAAGAACTAACGACTGCATTTCAATACTAAAAGGATTATATCGATTTTGG
GTTAACA
>family_nr:555408:family_nt:555408:genus_nr:66526:genus_nt:66526:speci
es_nr:66527:species_nt:66527:NR:AKT93753.1:NT:KT175741.1:VH00444:1:AAA
MMTVHV:1:1102:71478:20121/2
AGTTGTTAACCCAAAATCGATATAATCCTTTTAGTATTGAAATGCAGTCGTTAGTTCTTTTTTCCGGCAT
GTCTGGCTATTTGTTTGATATTATTTTAGACAAAATTAAATCTTTTGAAAAAGCCCTTGTTTTCTTTAGT
GTTACAT
>family_nr:555408:family_nt:555408:genus_nr:66526:genus_nt:66526:speci
es_nr:66527:species_nt:66527:NR:AKT93753.1:NT:KT175741.1:VH00444:1:AAA
MMTVHV:1:1113:10979:46075/1
CAATAGGCCGTGGTCAGCGTGAGTTGGTGATTGGTGACCGACAAACGGGTAAAACTGCTATTTGTATTGA
TGCTATACTAAACCAAAAATATGAAAATGATACAAAACGCTTTGAAAATTTTTTAT
>family_nr:555408:family_nt:555408:genus_nr:66526:genus_nt:66526:speci
es_nr:66527:species_nt:66527:NR:AKT93753.1:NT:KT175741.1:VH00444:1:AAA
MMTVHV:1:1113:10979:46075/2
ATAAAAAAATTTTCAAAGCGTTTTGTATCATTTTCATATTTTTGGTTTAGTATAGCATCAATACAAATAGC
AGTTTTACCCGTTTGTCGGTCACCAATCACCAACTCACGCTGACCACGGCCTATTG
>family_nr:555408:family_nt:555408:genus_nr:66526:genus_nt:66526:speci
```

es_nr:66527:species_nt:66527:NR:AKT93753.1:NT:KT175741.1:VH00444:1:AAA
MMTVHV:1:2505:48680:17168/1
GAAAACAAGGGCTTTTTCAAAAGATTTAATTTTGTCTAAAATAATATCAAACAAATAGCCAGACATGCCG
GAAAAAAGAACTAACGACTGCATTTCAATACTAAAAGGATTATATCGATTTTGGGTTAACAACTAAG
>family_nr:555408:family_nt:555408:genus_nr:66526:genus_nt:66526:speci
es_nr:66527:species_nt:66527:NR:AKT93753.1:NT:KT175741.1:VH00444:1:AAA
MMTVHV:1:2505:48680:17168/2
CTTAGTTGTTAACCCAAAATCGATATAATCCTTTTAGTATTGAAATGCAGTCGTTAGTTCTTTTTTCCGG
CATGTCTGGCTATTTGTTTGATATTATTTTAGACAAAATTAAATCTTTTGAAAAAGCCCTTGTTTTC
>family_nr:555408:family_nt:555408:genus_nr:66526:genus_nt:66526:speci
es_nr:66527:species_nt:66527:NR:AKT93753.1:NT:KT175741.1:VH00444:1:AAA
MMTVHV:1:2611:25181:44220/1
AGTATCTTTATTATAAAAGAATAAAAGCTCGCCGACTTTGGCGCGCTTTAAACCGTTTACGGTAATAACA
CCATCAATCACAGTACTAACGACACCGGTAAATAGTTCACTGAACTTCGATTGTTTTTGAGGAGATAAAA
CTTTATT
>family_nr:555408:family_nt:555408:genus_nr:66526:genus_nt:66526:speci
es_nr:66527:species_nt:66527:NR:AKT93753.1:NT:KT175741.1:VH00444:1:AAA
MMTVHV:1:2611:25181:44220/2
CTTGAACTTTTTATTTATTCAATGTTGAAATCGGGTAAGTTGAATAAAGTTTTATCTCCTCAAAAACAAT
CGAAGTTCAGTGAACTATTTACCGGTGTCGTTAGTACTGTGATTGATGGTGTTATTACCGTAAACGGTTT
AAAGCGC
>family_nr:555408:family_nt:555408:genus_nr:66526:genus_nt:66526:speci
es_nr:66527:species_nt:66527:NR:AKT93755.1:NT:KT175741.1:VH00444:1:AAA
MMTVHV:1:1506:54133:11261/1
TTTAGCATAGTCTTTATCCAAGCATACGTCTTTTTGACTTTGTTGTTAATTTATTTGAACGATTCCTTTA
ACCTGCACTAAGTGCGGTTCCTCCTCAATAATTCCCTGTTGCTTTTTGTTTATAACACATTGTTACTTTA
TCCGGTT
>family_nr:555408:family_nt:555408:genus_nr:66526:genus_nt:66526:speci
es_nr:66527:species_nt:66527:NR:AKT93755.1:NT:KT175741.1:VH00444:1:AAA
MMTVHV:1:1506:54133:11261/2
GGAACATAATACATAGCTAAAAAAATGCCGGTCACGATCTGGATAACCAAGCACAGTCCCGCCAAAACTC
CATAATTCCACCAATAACTAATGTTCAAATAAACCGGATAAAGTAACAATGTGTTATAAACAAAAAGCAA
CAGGGAA
>family_nr:555408:family_nt:555408:genus_nr:66526:genus_nt:66526:speci
es_nr:66527:species_nt:66527:NR:AKT93755.1:NT:KT175741.1:VH00444:1:AAA
MMTVHV:1:1611:68335:28337/1
GTTCAAATAAACCGGATAAAGTAACAATGTGTTATAAACAAAAAGCAACAGGGAATTATTGAGGAGGAAC
CGCACTTAGTGCAGGTTAAAGGAATCGTTCAAATAAATTAACAACAAAGTCAAAAAGACGTATGCTTGGA
TAAAGAC
>family_nr:555408:family_nt:555408:genus_nr:66526:genus_nt:66526:speci
es_nr:66527:species_nt:66527:NR:AKT93755.1:NT:KT175741.1:VH00444:1:AAA
MMTVHV:1:1611:68335:28337/2
ATAGTCTTTATCCAAGCATACGTCTTTTTGACTTTGTTGTTAATTTATTTGAACGATTCCTTTAACCTGC
ACTAAGTGCGGTTCCTCCTCAATAATTCCCTGTTGCTTTTTGTTTATAACACATTGTTACTTTATCCGGT
TTATTTG
>family_nr:555408:family_nt:555408:genus_nr:66526:genus_nt:66526:speci
es_nr:66527:species_nt:66527:NR:AKT93757.1:NT:KT030673.1:VH00444:1:AAA
MMTVHV:1:1602:59776:7248/1
ATAAAAAATAAATATTTGTTTAAAATATAAATTTTATCGTTATAAAAAGAAAAATGATAAAGAATACTCA
ATAATAAG

>family_nr:555408:family_nt:555408:genus_nr:66526:genus_nt:66526:species_nr:66527:species_nt:66527:NR:AKT93757.1:NT:KT175738.1:VH00444:1:AAAMMTVHV:1:2509:73864:13268/2
TATTACGTTTGAAAAATCTTCGTAGGTCCTTTTACAGTTATAATCGCTGTAATCGTCTCTGGGTTTTTCTCTCTA
>family_nr:555408:family_nt:555408:genus_nr:66526:genus_nt:66526:species_nr:66527:species_nt:66527:NR:AKT93757.1:NT:KT175741.1:VH00444:1:AAAMMTVHV:1:1602:59776:7248/2
CTTATTATTGAGTATTCTTTATCATTTTTCTTTTTATAACGATAAAATTTATATTTTAAACAAATATTTATTTTTTAT
>family_nr:555408:family_nt:555408:genus_nr:66526:genus_nt:66526:species_nr:66527:species_nt:66527:NR:AKT93757.1:NT:KT175741.1:VH00444:1:AAAMMTVHV:1:2509:73864:13268/1
TAGAGAGAAAAACCCAGAGACGATTACAGCGATTATAACTGTAAAAGGACCTACGAAGATTTTTCAAACGTAATA
>family_nr:555408:family_nt:555408:genus_nr:66526:genus_nt:66526:species_nr:66527:species_nt:66527:NR:AKT93766.1:NT:KT175739.1:VH00444:1:AAAMMTVHV:1:1210:24859:32729/2
AATCAATATTGGCAACCACAACACCATTAATTGGAACTACTAATCAGCCACACAAACTCAAAAAAAACATAAAAATGGGCGAAAAAACAAATAAAAATTTACTTGCACGGTTAGGAATT
>family_nr:555408:family_nt:555408:genus_nr:66526:genus_nt:66526:species_nr:66527:species_nt:66527:NR:AKT93766.1:NT:KT175741.1:VH00444:1:AAAMMTVHV:1:1210:24859:32729/1
AATTCCTAACCGTGCAAGTAAATTTTTATTTGTTTTTTCGCCCATTTTTATGTTTTTTTTGAGTTTGTGTGGCTGATTAGTAGTTCCAATTAATGGTGTTGTGGTTGCCAATATTGATT
>family_nr:555408:family_nt:555408:genus_nr:66526:genus_nt:66526:species_nr:66527:species_nt:66527:NR:AKT93766.1:NT:KT175741.1:VH00444:1:AAAMMTVHV:1:2614:67426:30192/1
TATTAACGATGTTCCCTTTTTTTGTATTATTTTTTATTGCTATTTTGGCTGAAACGAATCGTGTTCCTTTTGATTTACCAGAAGCCAAATCTGAACTTGTATCTGGTTATAATGTAG
>family_nr:555408:family_nt:555408:genus_nr:66526:genus_nt:66526:species_nr:66527:species_nt:66527:NR:AKT93766.1:NT:KT175741.1:VH00444:1:AAAMMTVHV:1:2614:67426:30192/2
CTACATTATAACCAGATACAAGTTCAGATTTGGCTTCTGGTAAATCAAAAGGAACACGATTCGTTTCAGCCAAAATAGCAATAAAAAATAATACAAAAAAAGGGAACATCGTTAATA
>family_nr:555408:family_nt:555408:genus_nr:66526:genus_nt:66526:species_nr:66527:species_nt:66527:NR:AKT93768.1:NT:KT175738.1:VH00444:1:AAAMMTVHV:1:1213:51331:46056/2
TATTATTTTCCAGCAAGAAGATATTGAAAAAATTTCGGCATATGAATGCGGTTTTCAGCCATTTGAAGATACACGAGTTAAGTTTGATGTTCGCTATTATTTGGTCGGCATTTTATT
>family_nr:555408:family_nt:555408:genus_nr:66526:genus_nt:66526:species_nr:66527:species_nt:66527:NR:AKT93768.1:NT:KT175739.1:VH00444:1:AAAMMTVHV:1:1213:51331:46056/1
AATAAAATGCCGACCAAATAATAGCGAACATCAAACTTAACTCGTGTATCTTCAAATGGCTGAAAACCGCATTCATATGCCGAAATTTTTTCAATATCTTCTTGCTGGAAAATAATA
>family_nr:555408:family_nt:555408:genus_nr:66526:genus_nt:66526:species_nr:66527:species_nt:66527:NR:AKT93771.1:NT:KT175741.1:VH00444:1:AAAMMTVHV:1:1109:66630:41816/1
AGCAAAAATAGTACCAAAGTTTGTACTTTTAAATACGAAAAGCATAAGGAGAATGCCAAAATACAGACCAAAATCACCAATACGATTTACCACAATGGCCTTAATAGCAGACTTAT

>family_nr:555408:family_nt:555408:genus_nr:66526:genus_nt:66526:speci
es_nr:66527:species_nt:66527:NR:AKT93771.1:NT:KT175741.1:VH00444:1:AAA
MMTVHV:1:1109:66630:41816/2
ATAAGTCTGCTATTAAGGCCATTGTGGTAAATCGTATTGGTGATTTTGGTCTGTATTTTGGCATTCTCCT
TATGCTTTTCGTATTTAAAAGTACAAACTTTGGTACTATTTTTGCT
>family_nr:555408:family_nt:555408:genus_nr:66526:genus_nt:66526:speci
es_nr:66527:species_nt:66527:NR:AKT93771.1:NT:KT175741.1:VH00444:1:AAA
MMTVHV:1:2502:15183:49558/1
AGAATAAAAAACGCTTAACACAAGTATGATGTTGAAAACAAAAAACATGTATTTATAATAAAAAAAGACG
AAAGCACCGT
>family_nr:555408:family_nt:555408:genus_nr:66526:genus_nt:66526:speci
es_nr:66527:species_nt:66527:NR:AKT93771.1:NT:KT175741.1:VH00444:1:AAA
MMTVHV:1:2502:15183:49558/2
ACGGTGCTTTCGTCTTTTTTTATTATAAATACATGTTTTTTGTTTTCAACATCATACTTGTGTTAAGCGT
TTTTTATTCT
>family_nr:555408:family_nt:555408:genus_nr:66526:genus_nt:66526:speci
es_nr:66527:species_nt:66527:NR:AKT93776.1:NT:KP888565.1:VH00444:1:AAA
MMTVHV:1:2210:71232:32710/2
ATAAATTTTTTCAAAAAAAAATTCATTACAAAGCTGGTTTTTTTAGATTAAAAGGGTGGCGTCCGGTGGT
ACGTGGCGTCGCTATGAATCC
>family_nr:555408:family_nt:555408:genus_nr:66526:genus_nt:66526:speci
es_nr:66527:species_nt:66527:NR:AKT93776.1:NT:KT030671.1:VH00444:1:AAA
MMTVHV:1:2504:35008:10087/1
CAAACGAGAAGAGAGCGCTTTAAATGATTTTTTTTTCCAAAGAGATAACATTAAGTCGTTTAAACATAAA
CCACAAACGAAGTGTAATAAAT
>family_nr:555408:family_nt:555408:genus_nr:66526:genus_nt:66526:speci
es_nr:66527:species_nt:66527:NR:AKT93776.1:NT:KT030673.1:VH00444:1:AAA
MMTVHV:1:2210:71232:32710/1
GGATTCATAGCGACGCCACGTACCACCGGACGCCACCCTTTTAATCTAAAAAAACCAGCTTTGTAATGAA
TTTTTTTTTGAAAAAATTTAT
>family_nr:555408:family_nt:555408:genus_nr:66526:genus_nt:66526:speci
es_nr:66527:species_nt:66527:NR:AKT93776.1:NT:KT175741.1:VH00444:1:AAA
MMTVHV:1:1112:22889:48744/1
ACCTCATCGACTGACAGAAGGGCGTCCCGCACCAGATTTTCCTTAACCGCCCCCATGGGGATGATCTACA
GGATTCATAGCG
>family_nr:555408:family_nt:555408:genus_nr:66526:genus_nt:66526:speci
es_nr:66527:species_nt:66527:NR:AKT93776.1:NT:KT175741.1:VH00444:1:AAA
MMTVHV:1:1112:22889:48744/2
CGCTATGAATCCTGTAGATCATCCCCATGGGGGCGGTTAAGGAAAATCTGGTGCGGGACGCCCTTCTGTC
AGTCGATGAGGT
>family_nr:555408:family_nt:555408:genus_nr:66526:genus_nt:66526:speci
es_nr:66527:species_nt:66527:NR:AKT93776.1:NT:KT175741.1:VH00444:1:AAA
MMTVHV:1:2504:35008:10087/2
ATTTATTACACTTCGTTTGTGGTTTATGTTTAAACGACTTAATGTTATCTCTTTGGAAAAAAAAATCATT
TAAAGCGCTCTCTTCTCGTTTG
>family_nr:555408:family_nt:555408:genus_nr:66526:genus_nt:66526:speci
es_nr:66527:species_nt:66527:NR:AKT93779.1:NT:KT175741.1:VH00444:1:AAA
MMTVHV:1:1304:40045:50183/1
ATACACCTAAAGAGGCGAAACGGCTCGGAGCATTTCCTGGAATGTTAATTCGTGCGTCTGTTCGTCGGGT
GATTCTTCGGAAAAAATTAAAAAAGAGTCGTGTGCTCAAACAGGGCCAGTTGTGCACAGCGTTGGTTGTG

CGGACTG
>family_nr:555408:family_nt:555408:genus_nr:66526:genus_nt:66526:species_nr:66527:species_nt:66527:NR:AKT93779.1:NT:KT175741.1:VH00444:1:AAAMMTVHV:1:1304:40045:50183/2
CTCACCGTTTAAAGCCATATACAGTCCGCACAACCAACGCTGTGCACAACTGGCCCTCTTTGAGCACACG
ACTCTTTTTTAATTTTTTCCGAAGAATCACCCGACGAACAGACGCACGAATTAACATTCCAGGAAATGCT
CCGAGCC
>family_nr:555408:family_nt:555408:genus_nr:66526:genus_nt:66526:species_nr:66527:species_nt:66527:NR:AKT93785.1:NT:KT175741.1:VH00444:1:AAAMMTVHV:1:1205:67104:37632/2
CAAAATCCTTGGATATTGTTACCAAAAATTCTTTTCATATTTTTCGGTCTTCGGAAAATACGCTTGCCAG
GTTTAGAAATACGTAAAATGCGACGTATACTTGGCCTATTATCAACAAACCGCAAAGACACAAGAATTTC
CGATTGA
>family_nr:555408:family_nt:555408:genus_nr:66526:genus_nt:66526:species_nr:66527:species_nt:66527:NR:AKT93786.1:NT:KT030672.1:VH00444:1:AAAMMTVHV:1:1108:69338:33316/1
GTTAGTCCGAAAACCGTTTGTGCCGTACTTACGCCGCCGTTTGGTTCACCGGTTTTGTATT
>family_nr:555408:family_nt:555408:genus_nr:66526:genus_nt:66526:species_nr:66527:species_nt:66527:NR:AKT93813.1:NT:KT175741.1:VH00444:1:AAAMMTVHV:1:1214:16205:36761/2
AACCCGAACTTACGACCGCTTTACAATCGCCGGCCCAGCCTTTTTTATAAAAAGAAGAATTCGCAAAATT
GCGAACAAACACACGGCGATTTGGTCGAATATAGGCGACGGATTTCCTAAAAAAACGACGCCGAAAAGAC
CAACGGA
>family_nr:555408:family_nt:555408:genus_nr:66526:genus_nt:66526:species_nr:66527:species_nt:66527:NR:AKT93813.1:NT:KT175741.1:VH00444:1:AAAMMTVHV:1:1404:76458:35853/1
ATAACGCTTTAATAAGCTAAAAACATGTAACAAAAGATTAAAAGCACGCAAACCAAAACCCTTTTGCAAA
TTATGGTGGATAAAGCCGTTAAC
>family_nr:555408:family_nt:555408:genus_nr:66526:genus_nt:66526:species_nr:66527:species_nt:66527:NR:AKT93813.1:NT:KT175741.1:VH00444:1:AAAMMTVHV:1:1404:76458:35853/2
GTTAACGGCTTTATCCACCATAATTTGCAAAAGGGTTTTGGTTTGCGTGCTTTTAATCTTTTGTTACATG
TTTTTAGCTTATTAAAGCGTTAT
>family_nr:555408:family_nt:555408:genus_nr:66526:genus_nt:66526:species_nr:66527:species_nt:66527:NR:AKT93813.1:NT:KT175741.1:VH00444:1:AAAMMTVHV:1:2210:73883:30022/1
CCCTTTTGCAAATTATGGTGGATAAAGCCGTTAACTAAAAAAACAGACCGCATTAAGAATCCGAACGCTT
AGATAAAGGCGCTCCATATTTTGAACGCTTTTTTGTTCGTGTAATATTTTCTCGTCA
>family_nr:555408:family_nt:555408:genus_nr:66526:genus_nt:66526:species_nr:66527:species_nt:66527:NR:AKT93813.1:NT:KT175741.1:VH00444:1:AAAMMTVHV:1:2210:73883:30022/2
TGACGAGAAAATATTACACGAACAAAAAAGCGTTCAAAATATGGAGCGCCTTTATCTAAGCGTTCGGATT
CTTAATGCGGTCTGTTTTTTTAGTTAACGGCTTTATCCACCATAATTTGCAAAAGGG
>family_nr:555408:family_nt:555408:genus_nr:66526:genus_nt:66526:species_nr:66527:species_nt:66527:NR:AKT93813.1:NT:KT175741.1:VH00444:1:AAAMMTVHV:1:2306:43075:49502/1
ATTCTGAAATAACGCTTTAATAAGCTAAAAACATGTAACAAAAGATTAAAAGCACGCAAACCAAAACCCT
TTTGCAAATTATGGTGGATAAAGCCGTTAACTAAAAAAACAGACCGCATTAAGAATCCGAACGCTTAGAT
AAAGGCG
>family_nr:555408:family_nt:555408:genus_nr:66526:genus_nt:66526:speci

es_nr:66527:species_nt:66527:NR:AKT93813.1:NT:KT175741.1:VH00444:1:AAA
MMTVHV:1:2306:43075:49502/2
GTGCGGTTTAGCCTTATGCGTGGAAAATATGACTTTAGTTGACGAGAAAATATTACACGAACAAAAAAGC
GTTCAAAATATGGAGCGCCTTTATCTAAGCGTTCGGATTCTTAATGCGGTCTGTTTTTTTAGTTAACGGC
TTTATCC
>family_nr:555408:family_nt:555408:genus_nr:66526:genus_nt:66526:speci
es_nr:66527:species_nt:66527:NR:AKT93813.1:NT:KT175741.1:VH00444:1:AAA
MMTVHV:1:2307:69887:5316/1
ATTTACGTATTGCGGCAGCGTTAACTCGCGGGTTTTTGCGTTCGCATTCTTTTTCTTCGAAGGGATTGTC
GCCGTTTTTGGTTAAAAATG
>family_nr:555408:family_nt:555408:genus_nr:66526:genus_nt:66526:speci
es_nr:66527:species_nt:66527:NR:AKT93813.1:NT:KT175741.1:VH00444:1:AAA
MMTVHV:1:2307:69887:5316/2
CATTTTTAACCAAAAACGGCGACAATCCCTTCGAAGAAAAAGAATGCGAACGCAAAAACCCGCGAGTTAA
CGCTGCCGCAATACGTAAAT
>family_nr:555408:family_nt:555408:genus_nr:66526:genus_nt:66526:speci
es_nr:66527:species_nt:66527:NR:AKT93813.1:NT:KT175741.1:VH00444:1:AAA
MMTVHV:1:2409:55932:1738/1
ACCATAATTTGCAAAAGGGTTTTGGTTTGCGTGCTTTTAATCTTTTGTTACATGTTTTTAGC
>family_nr:555408:family_nt:555408:genus_nr:66526:genus_nt:66526:speci
es_nr:66527:species_nt:66527:NR:AKT93813.1:NT:KT175741.1:VH00444:1:AAA
MMTVHV:1:2409:55932:1738/2
GCTAAAAACATGTAACAAAAGATTAAAAGCACGCAAACCAAAACCCTTTTGCAAATTATGGT
>family_nr:555408:family_nt:555408:genus_nr:66526:genus_nt:66526:speci
es_nr:66527:species_nt:66527:NR:AKT93823.1:NT:KP888565.1:VH00444:1:AAA
MMTVHV:1:2509:27377:55843/2
GTTGCCGCTCGAAAACTCATTTGACCTCACGGAAGAACATACCCTACAAACGCAGTAATAATCATTAACA
ATAAAATAATAACAC
>family_nr:555408:family_nt:555408:genus_nr:66526:genus_nt:66526:speci
es_nr:66527:species_nt:66527:NR:AKT93823.1:NT:KT030671.1:VH00444:1:AAA
MMTVHV:1:2509:27377:55843/1
GTGTTATTATTTTATTGTTAATGATTATTACTGCGTTTGTAGGGTATGTTCTTCCGTGAGGTCAAATGAG
TTTTCGAGCGGCAAC
>family_nr:555408:family_nt:555408:genus_nr:66526:genus_nt:66526:speci
es_nr:66527:species_nt:66527:NR:AKT93823.1:NT:KT175741.1:VH00444:1:AAA
MMTVHV:1:1502:32868:31158/1
TAAGCATCACATAACTCACCGAATAAGCGCCCCACAAAAGAATTAATATATCATTCCCTATAACAGGAAT
AG
>family_nr:555408:family_nt:555408:genus_nr:66526:genus_nt:66526:speci
es_nr:66527:species_nt:66527:NR:AKT93823.1:NT:KT175741.1:VH00444:1:AAA
MMTVHV:1:1502:32868:31158/2
CTATTCCTGTTATAGGGAATGATATATTAATTCTTTTGTGGGGCGCTTATTCGGTGAGTTATGTGATGCT
TA
>family_nr:555408:family_nt:555408:genus_nr:66526:genus_nt:66526:speci
es_nr:66527:species_nt:66527:NR:AKT93824.1:NT:KT030673.1:VH00444:1:AAA
MMTVHV:1:1209:68051:27902/1
GCAATTGTTCGTTCCTCAAAATGTTCCTAAGGCCCTTGTTCCTTTTCTTGTTGTTTTAGAAATTATTTCG
TACTTAATTAGGCCGTTTAGTTTATCTCTTCGGCTTTTTGCTAATATGATGGCAGGAC
>family_nr:555408:family_nt:555408:genus_nr:66526:genus_nt:66526:speci
es_nr:66527:species_nt:66527:NR:AKT93824.1:NT:KT175739.1:VH00444:1:AAA

MMTVHV:1:1209:68051:27902/2
GTCCTGCCATCATATTAGCAAAAAGCCGAAGAGATAAACTAAACGGCCTAATTAAGTACGAAATAATTTC
TAAAACAACAAGAAAAGGAACAAGGGCCTTAGGAACATTTTGAGGAACGAACAATTGC
>family_nr:555408:family_nt:555408:genus_nr:66526:genus_nt:66526:speci
es_nr:66527:species_nt:66527:NR:AKT93870.1:NT:KT175741.1:VH00444:1:AAA
MMTVHV:1:1209:76003:18361/1
ATACAAATTGCCTTGGAAATTTAACTTTTTTTGATCCCTTAATTTAATATTTATTTTGTTAGGCGTTTTG
GTTCTATGCTTTG
>family_nr:555408:family_nt:555408:genus_nr:66526:genus_nt:66526:speci
es_nr:66527:species_nt:66527:NR:AKT93870.1:NT:KT175741.1:VH00444:1:AAA
MMTVHV:1:1209:76003:18361/2
CAAAGCATAGAACCAAAACGCCTAACAAAATAAATATTAAATTAAGGGATCAAAAAAAGTTAAATTTCCA
AGGCAATTTGTAT
>family_nr:555408:family_nt:555408:genus_nr:66526:genus_nt:66526:speci
es_nr:66527:species_nt:66527:NR:AKT93870.1:NT:KT175741.1:VH00444:1:AAA
MMTVHV:1:1503:35690:41059/1
AAAACTCACAACACGGAAAAGACAAAAACGAATCCAACGAGAAGCGGATAGGATTGTATTTTAACTTCAA
CACATCGATTGTCAGCAAAGCATAGAACCAAAACGCCTAACAAAATAAATATTAAATTAAGGGATCAAAA
AAAGTTA
>family_nr:555408:family_nt:555408:genus_nr:66526:genus_nt:66526:speci
es_nr:66527:species_nt:66527:NR:AKT93870.1:NT:KT175741.1:VH00444:1:AAA
MMTVHV:1:1503:35690:41059/2
TTATAGTTTAATTTTGAAACAACAAACTAAATAATGAAACTATTTCTTAAATACAAATTGCCTTGGAAAT
TTAACTTTTTTTGATCCCTTAATTTAATATTTATTTTGTTAGGCGTTTTGGTTCTATGCTTTGCTGACAA
TCGATGT
>family_nr:555408:family_nt:555408:genus_nr:66526:genus_nt:66526:speci
es_nr:66527:species_nt:66527:NR:AKT93870.1:NT:KT175741.1:VH00444:1:AAA
MMTVHV:1:2103:48774:20860/1
AATGACTTAAACTCACGAATTCGGCATTACACAACAATTCTTCGAAGTCATCAATACCATATTCATCCTG
ATACAATTCCTCTAAAGCGTACATCACACA
>family_nr:555408:family_nt:555408:genus_nr:66526:genus_nt:66526:speci
es_nr:66527:species_nt:66527:NR:AKT93870.1:NT:KT175741.1:VH00444:1:AAA
MMTVHV:1:2103:48774:20860/2
TGTGTGATGTACGCTTTAGAGGAATTGTATCAGGATGAATATGGTATTGATGACTTCGAAGAATTGTTGT
GTAATGCCGAATTCGTGAGTTTAAGTCATT
>family_nr:555408:family_nt:555408:genus_nr:66526:genus_nt:66526:speci
es_nr:66527:species_nt:66527:NR:AKT93870.1:NT:KT175741.1:VH00444:1:AAA
MMTVHV:1:2201:73068:47116/1
CTCTTCGAAAATTTGCACAAGCCAATCAATAATATCAGGATTATGGGCAGCCGCCTGCTGCCCTAACTCA
CCACCACCTAGATCACTAACGGAAAAAGTTCCGGTACGAAAGGAGTTTAGCCACGAAAAATATCATGGGA
TCACAGA
>family_nr:555408:family_nt:555408:genus_nr:66526:genus_nt:66526:speci
es_nr:66527:species_nt:66527:NR:AKT93870.1:NT:KT175741.1:VH00444:1:AAA
MMTVHV:1:2201:73068:47116/2
TGCTACGTATCCGTTTTAACTGTGGCGTCTGTGATCCCATGATATTTTTCGTGGCTAAACTCCTTTCGTA
CCGGAACTTTTTCCGTTAGTGATCTAGGTGGTGGTGAGTTAGGGCAGCAGGCGGCTGCCCATAATCCTGA
TATTATT
>family_nr:555408:family_nt:555408:genus_nr:66526:genus_nt:66526:speci
es_nr:66527:species_nt:66527:NR:AKT93870.1:NT:KT175741.1:VH00444:1:AAA
MMTVHV:1:2502:48585:47893/1

CTCCTGAATGCCCATACGTGTTAATAACCGATAAACATAGCCACTCGCCACACTCATAAACACGCCCAAT
CCTACAAGCCCCAGACGAAAAT
>family_nr:555408:family_nt:555408:genus_nr:66526:genus_nt:66526:speci
es_nr:66527:species_nt:66527:NR:AKT93870.1:NT:KT175741.1:VH00444:1:AAA
MMTVHV:1:2502:48585:47893/2
ATTTTCGTCTGGGGCTTGTAGGATTGGGCGTGTTTATGAGTGTGGCGAGTGGCTATGTTTATCGGTTATT
AACACGTATGGGCATTCAGGAG
>family_nr:555408:family_nt:555408:genus_nr:66526:genus_nt:66526:speci
es_nr:66527:species_nt:66527:NR:AKT93870.1:NT:KT175741.1:VH00444:1:AAA
MMTVHV:1:2503:21128:38995/1
ACAACCTCTAATTTTTGGTGTTCTTTATTTGAAAACGTTAGAGATTTTGTTTGCGCGCACCCCTTTTTGT
GCTACGTATCCGTTTTAACT
>family_nr:555408:family_nt:555408:genus_nr:66526:genus_nt:66526:speci
es_nr:66527:species_nt:66527:NR:AKT93870.1:NT:KT175741.1:VH00444:1:AAA
MMTVHV:1:2503:21128:38995/2
AGTTAAAACGGATACGTAGCACAAAAAGGGGTGCGCGCAAACAAAATCTCTAACGTTTTCAAATAAAGAA
CACCAAAAATTAGAGGTTGT
>family_nr:555408:family_nt:555408:genus_nr:66526:genus_nt:66526:speci
es_nr:66527:species_nt:66527:NR:AKT93870.1:NT:KT175741.1:VH00444:1:AAA
MMTVHV:1:2512:30350:20803/1
AACACGGAAAAGACAAAAACGAATCCAACGAGAAGCGGATAGGATTGTATTTTAACTTCAACACATCGAT
TGTCAGCAAAGCATAGAACCAAAACGCCTAACAAAATAAATATTAAATTAAG
>family_nr:555408:family_nt:555408:genus_nr:66526:genus_nt:66526:speci
es_nr:66527:species_nt:66527:NR:AKT93870.1:NT:KT175741.1:VH00444:1:AAA
MMTVHV:1:2512:30350:20803/2
CTTAATTTAATATTTATTTTGTTAGGCGTTTTGGTTCTATGCTTTGCTGACAATCGATGTGTTGAAGTTA
AAATACAATCCTATCCGCTTCTCGTTGGATTCGTTTTTGTCTTTTCCGTGTT
>family_nr:555408:family_nt:555408:genus_nr:66526:genus_nt:66526:speci
es_nr:66527:species_nt:66527:NR:AKT93871.1:NT:KT175741.1:VH00444:1:AAA
MMTVHV:1:1203:40348:12132/1
ATAATCATCGTGGCTGCTGTAAAATAAGCACGTGTGTCTACATCTAAACCAACGGTATACATATGATGAG
CCCATACAATAAAACCAAGA
>family_nr:555408:family_nt:555408:genus_nr:66526:genus_nt:66526:speci
es_nr:66527:species_nt:66527:NR:AKT93871.1:NT:KT175741.1:VH00444:1:AAA
MMTVHV:1:1203:40348:12132/2
TCTTGGTTTTATTGTATGGGCTCATCATATGTATACCGTTGGTTTAGATGTAGACACACGTGCTTATTTT
ACAGCAGCCACGATGATTAT
>family_nr:555408:family_nt:555408:genus_nr:66526:genus_nt:66526:speci
es_nr:66527:species_nt:66527:NR:AKT93871.1:NT:KT175741.1:VH00444:1:AAA
MMTVHV:1:1409:32925:34395/1
TTTATTCTGGTTTTTTGGTCATCCTGAAGTTTACATTCTAATATTGCCTGGTTTTGGTATTGTAAGCCAA
ATAATTTGTTCATTTTCTGATAAACAAATTTTTGGGTACATCGGTATGGTCTATGCTATGGTTTCCATAG
GAATTCT
>family_nr:555408:family_nt:555408:genus_nr:66526:genus_nt:66526:speci
es_nr:66527:species_nt:66527:NR:AKT93871.1:NT:KT175741.1:VH00444:1:AAA
MMTVHV:1:1409:32925:34395/2
TAAACCCAAGAACAAATAACAACGGTGTTTTAAATCGTATATTACCAAAGGCAAGCGTTGCGATCCAACT
AAAAATTTTTATACCAGTTGGAACGGCAATAATCATCGTGGCTGCTGTAAAATAAGCACGTGTGTCTACA
TCTAAAC
>family_nr:555408:family_nt:555408:genus_nr:66526:genus_nt:66526:speci

es_nr:66527:species_nt:66527:NR:AKT93871.1:NT:KT175741.1:VH00444:1:AAA
MMTVHV:1:2301:36807:4881/1
CTCCGGTCAGACCACCAATTGTAAATAAAATAATAAACCCAAGAACAAATAACAACGGTGTTTTAAATCG
TATATTACCAAAGGCAAGCGTTGCGATCCAACTAAAAATTTT
>family_nr:555408:family_nt:555408:genus_nr:66526:genus_nt:66526:speci
es_nr:66527:species_nt:66527:NR:AKT93871.1:NT:KT175741.1:VH00444:1:AAA
MMTVHV:1:2301:36807:4881/2
AAAATTTTTAGTTGGATCGCAACGCTTGCCTTTGGTAATATACGATTTAAAACACCGTTGTTATTTGTTC
TTGGGTTTATTATTTTATTTACAATTGGTGGTCTGACCGGAG
>family_nr:555408:family_nt:555408:genus_nr:66526:genus_nt:66526:speci
es_nr:66527:species_nt:66527:NR:AKT93872.1:NT:KT030672.1:VH00444:1:AAA
MMTVHV:1:2514:9691:28773/1
AAATAACAATTTAAAAGAGTCACCACATCTTGTCAAAGACTAAAATTACGTCCATATCCGTGCGGCATGC
GTTTTCTCACTCCTACCTCTACACTGTGACGTAAAAAGCGATAACGCTCACTGCCTTTAGG
>family_nr:555408:family_nt:555408:genus_nr:66526:genus_nt:66526:speci
es_nr:66527:species_nt:66527:NR:AKT93872.1:NT:KT175739.1:VH00444:1:AAA
MMTVHV:1:1314:46597:15994/1
CTACTTTTCGTCCATCGTATTTCAAGTTCACTGGGGGATATTTCCGCATTCCGTTTGTGTT
>family_nr:555408:family_nt:555408:genus_nr:66526:genus_nt:66526:speci
es_nr:66527:species_nt:66527:NR:AKT93872.1:NT:KT175740.1:VH00444:1:AAA
MMTVHV:1:1314:46597:15994/2
AACACAAACGGAATGCGGAAATATCCCCCAGTGAACTTGAAATACGATGGACGAAAAGTAG
>family_nr:555408:family_nt:555408:genus_nr:66526:genus_nt:66526:speci
es_nr:66527:species_nt:66527:NR:AKT93872.1:NT:KT175741.1:VH00444:1:AAA
MMTVHV:1:1303:39950:35550/1
TTGCTTTTTAGAACGCTTGAAATGATCACCCGATACGTGCGGTATACGGAACCCTTCTTCATAATCACCA
AAAGCAACCCGCGGATTTCCATAACTTAATTGCAAAATAAAATTTTTTGGATTTTTCAAACGATTTCTAA
TAAAACC
>family_nr:555408:family_nt:555408:genus_nr:66526:genus_nt:66526:speci
es_nr:66527:species_nt:66527:NR:AKT93872.1:NT:KT175741.1:VH00444:1:AAA
MMTVHV:1:1303:39950:35550/2
GTTTTGGCCGGTTTTTCGATATCCAAAATTAGCCGCCCGGCTGTTGGTTTTTATTTACAGTTGACTAGAA
CAGGACGTGCGCGCACAGCGTGACGGTCTCTTCGTTCTTATTTAGCTACTGGTTTTATTAGAAATCGTTT
GAAAAAT
>family_nr:555408:family_nt:555408:genus_nr:66526:genus_nt:66526:speci
es_nr:66527:species_nt:66527:NR:AKT93872.1:NT:KT175741.1:VH00444:1:AAA
MMTVHV:1:1312:60514:43690/1
TAACTTAATTGCAAAATAAAATTTTTTGGATTTTTCAAACGATTTCTAATAAAACCAG
>family_nr:555408:family_nt:555408:genus_nr:66526:genus_nt:66526:speci
es_nr:66527:species_nt:66527:NR:AKT93872.1:NT:KT175741.1:VH00444:1:AAA
MMTVHV:1:1312:60514:43690/2
CTGGTTTTATTAGAAATCGTTTGAAAAATCCAAAAAATTTTATTTTGCAATTAAGTTA
>family_nr:555408:family_nt:555408:genus_nr:66526:genus_nt:66526:speci
es_nr:66527:species_nt:66527:NR:AKT93872.1:NT:KT175741.1:VH00444:1:AAA
MMTVHV:1:1411:54834:47590/1
GAAGAGACCGTCACGCTGTGCGCGCACGTCCTGTTCTAGTCAACTGTAAATAAAAACCAACAGCCGGGCG
GCTAATTTTGGATATCGAAAAACCGGCCAAAACCGGTGAAGTAACTTCGTTCCCAAATAACCATGATGAG
TCCGCAG
>family_nr:555408:family_nt:555408:genus_nr:66526:genus_nt:66526:speci
es_nr:66527:species_nt:66527:NR:AKT93872.1:NT:KT175741.1:VH00444:1:AAA

MMTVHV:1:1411:54834:47590/2
CTAATAAAAAGGTGCCTCGTTTGGTTTATTTTTCAATGCGTCATATTTTGTCTCGGTTTGCTTTGTCCCA
TAATGTACTATTGTCAAAAATCCGAACTAGCTTGGTTTATCCGTATTTTTCATTTTCTTCGTTGGACCCT
GCGGACT
>family_nr:555408:family_nt:555408:genus_nr:66526:genus_nt:66526:speci
es_nr:66527:species_nt:66527:NR:AKT93872.1:NT:KT175741.1:VH00444:1:AAA
MMTVHV:1:1513:65134:20670/1
CACACCTGCGCCAGTTTAAGAAAGCGGGGCCATCACTGAACCTCATTTTTATAAAATTCAATCAAGTTTT
AAATTTAATGTATTGCGTTTTTTACTAAACTATAACGATCAAAAGAAGACCTCGTCGAATGAAAAGGAAC
TGTAAGT
>family_nr:555408:family_nt:555408:genus_nr:66526:genus_nt:66526:speci
es_nr:66527:species_nt:66527:NR:AKT93872.1:NT:KT175741.1:VH00444:1:AAA
MMTVHV:1:1513:65134:20670/2
ATTCCATTAAAGCATCAACTTACAGTTCCTTTTCATTCGACGAGGTCTTCTTTTGATCGTTATAGTTTAG
TAAAAAACGCAATACATTAAATTTAAAACTTGATTGAATTTTATAAAAATGAGGTTCAGTGATGGCCCCG
CTTTCTT
>family_nr:555408:family_nt:555408:genus_nr:66526:genus_nt:66526:speci
es_nr:66527:species_nt:66527:NR:AKT93872.1:NT:KT175741.1:VH00444:1:AAA
MMTVHV:1:1602:28835:3385/1
ATCCAAAATTAGCCGCCCGGCTGTTGGTTTTTATTTACAGTTGACTAGAACAGGACGTGCGCGCACAGCG
TGACGGTCTCTTCGTTCTTATTTAGCTACTGGTTTTATTAGAAATCGTTTGAAAAATCCAAAAAATTTTA
TTTTGCA
>family_nr:555408:family_nt:555408:genus_nr:66526:genus_nt:66526:speci
es_nr:66527:species_nt:66527:NR:AKT93872.1:NT:KT175741.1:VH00444:1:AAA
MMTVHV:1:1602:28835:3385/2
CTTCATAATCACCAAAAGCAACCCGCGGATTTCCATAACTTAATTGCAAAATAAAATTTTTTGGATTTTT
CAAACGATTTCTAATAAAACCAGTAGCTAAATAAGAACGAAGAGACCGTCACGCTGTGCGCGCACGTCCT
GTTCTAG
>family_nr:555408:family_nt:555408:genus_nr:66526:genus_nt:66526:speci
es_nr:66527:species_nt:66527:NR:AKT93872.1:NT:KT175741.1:VH00444:1:AAA
MMTVHV:1:1607:42071:1700/1
GAAAAATAAACCAAACGAGGCACCTTTTTATTAGTTTTCGTTGTAACTTTTGCCAAACGCTTTATACTAC
GTAAAACAGGACGAGAAGACTTGATGTTCTGGCGACCCATAGGTTTTATAAAGCTCAACCAACGCCATAA
CGCAGGT
>family_nr:555408:family_nt:555408:genus_nr:66526:genus_nt:66526:speci
es_nr:66527:species_nt:66527:NR:AKT93872.1:NT:KT175741.1:VH00444:1:AAA
MMTVHV:1:1607:42071:1700/2
GCACTTATCTCTTATTAATATTTTGCTTGAAAAGGTACGTTGCCAATTATCCGAAAATCTGCGTTTAAAA
TTACCTGCGTTATGGCGTTGGTTGAGCTTTATAAAACCTATGGGTCGCCAGAACATCAAGTCTTCTCGTC
CTGTTTT
>family_nr:555408:family_nt:555408:genus_nr:66526:genus_nt:66526:speci
es_nr:66527:species_nt:66527:NR:AKT93872.1:NT:KT175741.1:VH00444:1:AAA
MMTVHV:1:2103:53508:47097/2
TGCCTTGTTAGCATGTTCCCTCAGATATCTTTACCCGTCAATCTAATTAACTTATATTTGTTTACCTGTA
AATCACGTCGCATTTTAAATTTTTAAAGATACAGTACCAAAAGCACGTCGGGCTAACAATTTTTTTTTTC
GACGTAA
>family_nr:555408:family_nt:555408:genus_nr:66526:genus_nt:66526:speci
es_nr:66527:species_nt:66527:NR:AKT93872.1:NT:KT175741.1:VH00444:1:AAA
MMTVHV:1:2205:33058:30817/1
CTGTAAGTTGATGCTTTAATGGAATACCCACCAACGCAGCCGCTAACAGAACATAATTGGGCTCACAAAA

CAAAGGCATGTTCAAAATCAGACGAAAATTAACCAACTTATGCATAAAAACATTCAAAGCTCGTCAATAA
AAGACAG
>family_nr:555408:family_nt:555408:genus_nr:66526:genus_nt:66526:speci
es_nr:66527:species_nt:66527:NR:AKT93872.1:NT:KT175741.1:VH00444:1:AAA
MMTVHV:1:2205:33058:30817/2
GTCTTTTATTGACGAGCTTTGAATGTTTTTATGCATAAGTTGGTTAATTTTCGTCTGATTTTGAACATGC
CTTTGTTTTGTGAGCCCAATTATGTTCTGTTAGCGGCTGCGTTGGTGGGTATTCCATTAAAGCATCAACT
TACAGAG
>family_nr:555408:family_nt:555408:genus_nr:66526:genus_nt:66526:speci
es_nr:66527:species_nt:66527:NR:AKT93872.1:NT:KT175741.1:VH00444:1:AAA
MMTVHV:1:2312:67880:9292/1
CAAATATTACAGTCATCCGAAACATCGTAAAATGCTTACTCTTTTATCCATCATTGATAAAAAATTTTTA
GGACCTACTGCATGGGGTGTGCACTTATCTCTTATTAATATTTTGCTTGAAA
>family_nr:555408:family_nt:555408:genus_nr:66526:genus_nt:66526:speci
es_nr:66527:species_nt:66527:NR:AKT93872.1:NT:KT175741.1:VH00444:1:AAA
MMTVHV:1:2312:67880:9292/2
TTTCAAGCAAAATATTAATAAGAGATAAGTGCACACCCCATGCAGTAGGTCCTAAAAATTTTTTATCAAT
GATGGATAAAAGAGTAAGCATTTTACGATGTTTCGGATGACTGTAATATTTG
>family_nr:555408:family_nt:555408:genus_nr:66526:genus_nt:66526:speci
es_nr:66527:species_nt:66527:NR:AKT93872.1:NT:KT175741.1:VH00444:1:AAA
MMTVHV:1:2514:9691:28773/2
CCTAAAGGCAGTGAGCGTTATCGCTTTTTACGTCACAGTGTAGAGGTAGGAGTGAGAAAACGCATGCCGC
ACGGATATGGACGTAATTTTAGTCTTTGACAAGATGTGGTGACTCTTTTAAATTGTTATTT
>family_nr:555408:family_nt:555408:genus_nr:66526:genus_nt:66526:speci
es_nr:66527:species_nt:66527:NR:AKT93874.1:NT:KT175741.1:VH00444:1:AAA
MMTVHV:1:1108:14520:42459/1
TATATTTTTGGGAAATTTTACGTCTGAAATCTGGTCTTTTTCGTTGTATTATTTGTTTATTTCTAATTTA
TTTTTGCTTGTTTTTATTATGTGTTTGTTTGTTTTGCGTTGTCCAATTACTCAACGGCTGTTG
>family_nr:555408:family_nt:555408:genus_nr:66526:genus_nt:66526:speci
es_nr:66527:species_nt:66527:NR:AKT93874.1:NT:KT175741.1:VH00444:1:AAA
MMTVHV:1:1108:14520:42459/2
CAACAGCCGTTGAGTAATTGGACAACGCAAAACAAACAAACACATAATAAAAACAAGCAAAAATAAATTA
GAAATAAACAAATAATACAACGAAAAAGACCAGATTTCAGACGTAAAATTTCCCAAAAATATA
>family_nr:555408:family_nt:555408:genus_nr:66526:genus_nt:66526:speci
es_nr:66527:species_nt:66527:NR:AKT93875.1:NT:KT175741.1:VH00444:1:AAA
MMTVHV:1:1311:45839:22469/1
TGAGACAAATGCTTTATTTGTAAATATTTGTGGTGTGGTCCAAAGAGCTAATTGTATTTTTCATTTAGAT
AAGTTCCGCCACGCTCGATCTTCGTGAAAAATTTTTCGGGCCATGTCAAGCGTTGCGGGGGGATGCTCTG
TGTAGAT
>family_nr:555408:family_nt:555408:genus_nr:66526:genus_nt:66526:speci
es_nr:66527:species_nt:66527:NR:AKT93875.1:NT:KT175741.1:VH00444:1:AAA
MMTVHV:1:1311:45839:22469/2
ACACAGAGCATCCCCCCGCAACGCTTGACATGGCCCGAAAAATTTTTCACGAAGATCGAGCGTGGCGGAA
CTTATCTAAATGAAAAATACAATTAGCTCTTTGGACCACACCACAAATATTTACAAATAAAGCATTTGTC
TCAAGAT
>family_nr:555408:family_nt:555408:genus_nr:66526:genus_nt:66526:speci
es_nr:66527:species_nt:66527:NR:AKT93875.1:NT:KT175741.1:VH00444:1:AAA
MMTVHV:1:1404:18686:10314/1
GTTATATTTTGATAACTTAAATATACGTTATCAATCTCCTGTTTTAAATATTTTATTTCGCAATTCTTAT
TTGAAAAATAACACACTAGTTGTATAT

>family_nr:555408:family_nt:555408:genus_nr:66526:genus_nt:66526:speci
es_nr:66527:species_nt:66527:NR:AKT93875.1:NT:KT175741.1:VH00444:1:AAA
MMTVHV:1:1404:18686:10314/2
ATATACAACTAGTGTGTTATTTTTCAAATAAGAATTGCGAAATAAAATATTTAAAACAGGAGATTGATAA
CGTATATTTAAGTTATCAAAATATAAC
>family_nr:555408:family_nt:555408:genus_nr:66526:genus_nt:66526:speci
es_nr:66527:species_nt:66527:NR:AKT93875.1:NT:KT175741.1:VH00444:1:AAA
MMTVHV:1:1601:47108:22942/1
CACACAGAGCATCCCCCCGCAACGCTTGACATGGCCCGAAAAATTTTTCACGAAGATCGAGCGTGGCGGA
ACTTATCTAAATGAAAAATACAATTAGCTCTTTGGACCACACCACAAATATTTACAAATAAAGCAT
>family_nr:555408:family_nt:555408:genus_nr:66526:genus_nt:66526:speci
es_nr:66527:species_nt:66527:NR:AKT93875.1:NT:KT175741.1:VH00444:1:AAA
MMTVHV:1:1601:47108:22942/2
ATGCTTTATTTGTAAATATTTGTGGTGTGGTCCAAAGAGCTAATTGTATTTTTCATTTAGATAAGTTCCG
CCACGCTCGATCTTCGTGAAAAATTTTTCGGGCCATGTCAAGCGTTGCGGGGGGATGCTCTGTGTG
>family_nr:555408:family_nt:555408:genus_nr:66526:genus_nt:66526:speci
es_nr:66527:species_nt:66527:NR:AKT93880.1:NT:KT175739.1:VH00444:1:AAA
MMTVHV:1:2604:76856:24097/1
ATTAAAAGACGTTTCTTGCTCAATACTAAACCGATCCAAAAGAGGGGTTGCACGCTAGTATATAGTTTTA
TCTACAGATACATAAGGTTTACTCGATAAAGGTAACCGTAAAAAACCTAAAACCTTATGCGAACGGTTAT
TATTAAC
>family_nr:555408:family_nt:555408:genus_nr:66526:genus_nt:66526:speci
es_nr:66527:species_nt:66527:NR:AKT93880.1:NT:KT175741.1:VH00444:1:AAA
MMTVHV:1:1301:8517:26425/1
TACGTTAATAATAACCGTTCGCATAAGGTTTTAGGTTTTTTACGGTTACCTTTATCGAGTAAACCTTATG
TATCTGTAGATAAAACTATATACAAGCGTGCAACCCCTCTTTTGGATCGGTTTAGTATTGAGCAAGAAAC
GTCTTTT
>family_nr:555408:family_nt:555408:genus_nr:66526:genus_nt:66526:speci
es_nr:66527:species_nt:66527:NR:AKT93880.1:NT:KT175741.1:VH00444:1:AAA
MMTVHV:1:1301:8517:26425/2
ATTAAAAGACGTTTCTTGCTCAATACTAAACCGATCCAAAAGAGGGGTTGCACGCTTGTATATAGTTTTA
TCTACAGATACATAAGGTTTACTCGATAAAGGTAACCGTAAAAAACCTAAAACCTTATGCGAACGGTTAT
TATTAAC
>family_nr:555408:family_nt:555408:genus_nr:66526:genus_nt:66526:speci
es_nr:66527:species_nt:66527:NR:AKT93881.1:NT:KT175741.1:VH00444:1:AAA
MMTVHV:1:1111:48850:29227/1
TCATTCTTCTCAAAGGATCGTTTCTTGGCTTCAATATTTTTTACATGATCAAGAATTTCCTAGTTTTTTG
GATTCGCTTAAAACTGGTAGTTTAGGTCTAAAAATGTTTATTCGTGAAAACGAACTTTTTATTGATTTAG
GTTATAG
>family_nr:555408:family_nt:555408:genus_nr:66526:genus_nt:66526:speci
es_nr:66527:species_nt:66527:NR:AKT93881.1:NT:KT175741.1:VH00444:1:AAA
MMTVHV:1:1111:48850:29227/2
CGGCAATCGAAACAAACAAAAATGACTATAACCTAAATCAATAAAAAGTTCGTTTTCACGAATAAACATT
TTTAGACCTAAACTACCAGTTTTAAGCGAATCCAAAAAACTAGGAAATTCTTGATCATGTAAAAAATATT
GAAGCCA
>family_nr:555408:family_nt:555408:genus_nr:66526:genus_nt:66526:speci
es_nr:66527:species_nt:66527:NR:AKT93881.1:NT:KT175741.1:VH00444:1:AAA
MMTVHV:1:1204:32849:36440/1
ATCAAGAATTTCCTAGTTTTTTGGATTCGCTTAAAACTGGTAGTTTAGGTCTAAAAATGTTTATTCGTGA
AAACGAACTTTTTATTGATTTAGGTTATAGTCATTTTTGTTTGTTTCGATT

>family_nr:555408:family_nt:555408:genus_nr:66526:genus_nt:66526:speci
es_nr:66527:species_nt:66527:NR:AKT93881.1:NT:KT175741.1:VH00444:1:AAA
MMTVHV:1:1204:32849:36440/2
AATCGAAACAAACAAAAATGACTATAACCTAAATCAATAAAAAGTTCGTTTTCACGAATAAACATTTTTA
GACCTAAACTACCAGTTTTAAGCGAATCCAAAAAACTAGGAAATTCTTGAT
>family_nr:555408:family_nt:555408:genus_nr:66526:genus_nt:66526:speci
es_nr:66527:species_nt:66527:NR:AKT93881.1:NT:KT175741.1:VH00444:1:AAA
MMTVHV:1:2213:49248:15729/1
AGTATTGCCATTTCTTCTCATTCTTCTCAAAGGATCGTTTCTTGGCTTCAATATTTTTTACATGATCAAG
AATTTCCTAGTTTTTTGGATTCGCTTAAAACTGGTAGTTTA
>family_nr:555408:family_nt:555408:genus_nr:66526:genus_nt:66526:speci
es_nr:66527:species_nt:66527:NR:AKT93881.1:NT:KT175741.1:VH00444:1:AAA
MMTVHV:1:2213:49248:15729/2
TAAACTACCAGTTTTAAGCGAATCCAAAAAACTAGGAAATTCTTGATCATGTAAAAAATATTGAAGCCAA
GAAACGATCCTTTGAGAAGAATGAGAAGAAATGGCAATACT
>family_nr:555408:family_nt:555408:genus_nr:66526:genus_nt:66526:speci
es_nr:66527:species_nt:66527:NR:AKT93881.1:NT:KT175741.1:VH00444:1:AAA
MMTVHV:1:2414:53792:5998/1
CAAAAAACTAGGAAATTCTTGATCATGTAAAAAATATTGAAGCCAAGAAACGATCCTTTGAGAAGAATGA
GAAGAAATGGCAATACTTGCACTCCAGGCATTCGGCCCAACCACTGATTTCGAAAAAGGATCATATTTAA
GATTCGA
>family_nr:555408:family_nt:555408:genus_nr:66526:genus_nt:66526:speci
es_nr:66527:species_nt:66527:NR:AKT93881.1:NT:KT175741.1:VH00444:1:AAA
MMTVHV:1:2414:53792:5998/2
TTATTTCGAATCTTAAATATGATCCTTTTTCGAAATCAGTGGTTGGGCCGAATGCCTGGAGTGCAAGTAT
TGCCATTTCTTCTCATTCTTCTCAAAGGATCGTTTCTTGGCTTCAATATTTTTTACATGATCAAGAATTT
CCTAGTT
>family_nr:555408:family_nt:555408:genus_nr:66526:genus_nt:66526:speci
es_nr:66527:species_nt:66527:NR:AKT93882.1:NT:KT175741.1:VH00444:1:AAA
MMTVHV:1:1205:67104:37632/1
GCCCGTAACTCGCGGTGTTTTTTTAACATTTTACATTTGGTAATGCATGACCTGCTTAGTATTGCCATTT
CACGTATTAGTGTTGCAGTGCTTTCTAAAAAGCCGTATACTATTGTGCCTTATTCCGGTTTAATTTTGTC
GTGTATG
>family_nr:555408:family_nt:555408:genus_nr:66526:genus_nt:66526:speci
es_nr:66527:species_nt:66527:NR:AKT93885.1:NT:KT030672.1:VH00444:1:AAA
MMTVHV:1:1103:18837:54234/2
ATTTATTTATGACATAAAGAGCTTTTTGATTATTTATTTCATTTATAAATAAAACAAAAAAACC
>family_nr:555408:family_nt:555408:genus_nr:66526:genus_nt:66526:speci
es_nr:66527:species_nt:66527:NR:AKT93885.1:NT:KT175740.1:VH00444:1:AAA
MMTVHV:1:1103:18837:54234/1
GGTTTTTTTGTTTTATTTATAAATGAAATAAATAATCAAAAAGCTCTTTATGTCATAAATAAAT
>family_nr:555408:family_nt:555408:genus_nr:66526:genus_nt:66526:speci
es_nr:66527:species_nt:66527:NR:AKT93885.1:NT:KT175741.1:VH00444:1:AAA
MMTVHV:1:1411:37432:49502/1
TTCCCCCTCGTTTATTTACGAGCGTCATATTTTAGCTTTATCTCGGGTGCTTAAATTTCATTTCAAGCGT
AAAAAAAAAGCGAGTTTTTTTCGCATTAATTTTTTTCTTAATAAATTTGTAACGAAAAAAGCGATTGGGG
TGCGTAT
>family_nr:555408:family_nt:555408:genus_nr:66526:genus_nt:66526:speci
es_nr:66527:species_nt:66527:NR:AKT93885.1:NT:KT175741.1:VH00444:1:AAA
MMTVHV:1:1411:37432:49502/2

CTTTTTCACTAGTGGCACCTTTCCCCTTACCCATACGCACCCCAATCGCTTTTTTCGTTACAAATTTATT
AAGAAAAAAATTAATGCGAAAAAAACTCGCTTTTTTTTTACGCTTGAAATGAAATTTAAGAACCCGAGAT
AAAGCTA
>family_nr:555408:family_nt:555408:genus_nr:66526:genus_nt:66526:speci
es_nr:66527:species_nt:66527:NR:AKT93885.1:NT:KT175741.1:VH00444:1:AAA
MMTVHV:1:1414:44798:37481/1
AAAAAACTTTTTCACTAGTGGCACCTTTCCCCTTACCCATACGCACCCCAATCGCTTTTTTCGTTACAAA
TTTATTAAGAAAAAAATTAATGCGAAAAAAACTCGCTTTTTTTTTACGCTTGAAATG
>family_nr:555408:family_nt:555408:genus_nr:66526:genus_nt:66526:speci
es_nr:66527:species_nt:66527:NR:AKT93885.1:NT:KT175741.1:VH00444:1:AAA
MMTVHV:1:1414:44798:37481/2
CATTTCAAGCGTAAAAAAAAAGCGAGTTTTTTTCGCATTAATTTTTTTCTTAATAAATTTGTAACGAAAA
AAGCGATTGGGGTGCGTATGGGTAAGGGGAAAGGTGCCACTAGTGAAAAAGTTTTTT
>family_nr:555408:family_nt:555408:genus_nr:66526:genus_nt:66526:speci
es_nr:66527:species_nt:66527:NR:AKT93885.1:NT:KT175741.1:VH00444:1:AAA
MMTVHV:1:1604:54606:35360/1
GTGGCTTCCTTTCTACTAAAGCTTATGTGGCCTCTTCTGAGTATTCCGGTTTTATTAAGATTGCGTTGCC
CATACGCACCCCAATCGCTTTTTTCGTTACAAATTTATTAAGAAAAAAATTAATGCGAAAAAAACTCG
>family_nr:555408:family_nt:555408:genus_nr:66526:genus_nt:66526:speci
es_nr:66527:species_nt:66527:NR:AKT93885.1:NT:KT175741.1:VH00444:1:AAA
MMTVHV:1:1604:54606:35360/2
CGAGTTTTTTTCGCATTAATTTTTTTCTTAATAAATTTGTAACGAAAAAAGCGATTGGGGTGCGTATGGG
CAACGCAATCTTAATAAAACCGGAATACTCAGAAGAGGCCACATAAGCTTTAGTAGAAAGGAAGCCAC
>family_nr:555408:family_nt:555408:genus_nr:66526:genus_nt:66526:speci
es_nr:66527:species_nt:66527:NR:AKT93886.1:NT:KT175741.1:VH00444:1:AAA
MMTVHV:1:1104:67710:38919/1
CTTGGTTAATGGGTAACCGAAAGCCTGGTTATGGAG
>family_nr:555408:family_nt:555408:genus_nr:66526:genus_nt:66526:speci
es_nr:66527:species_nt:66527:NR:AKT93886.1:NT:KT175741.1:VH00444:1:AAA
MMTVHV:1:1104:67710:38919/2
CTCCATAACCAGGCTTTCGGTTACCCATTAACCAAG
>family_nr:555408:family_nt:555408:genus_nr:66526:genus_nt:66526:speci
es_nr:66527:species_nt:66527:NR:AKT93886.1:NT:KT175741.1:VH00444:1:AAA
MMTVHV:1:1105:16395:21920/1
GCTTGGTCGAAAACTGCCAAAAAAAAAATAAATGGGTCATGTTGTTAATCCTATAAGTCATCGTATTTAT
GCAACGTTCTTTCATGATAAAATTTGACATGTTCGTTATCCGTTCTTCTACTCTTTTGTTTATTTTAAGT
TTGCATT
>family_nr:555408:family_nt:555408:genus_nr:66526:genus_nt:66526:speci
es_nr:66527:species_nt:66527:NR:AKT93886.1:NT:KT175741.1:VH00444:1:AAA
MMTVHV:1:1105:16395:21920/2
AACGAACATCGCTCTCCAAAGAAAACGTACGAAAACGTTTTAAGTTTAAAACCTGGGAAGCATACGTGTG
CGCCAATGCAAACTTAAAATAAACAAAAGAGTAGAAGAACGGATAACGAACATGTCAAATTTTATCATGA
AAGAACG
>family_nr:555408:family_nt:555408:genus_nr:66526:genus_nt:66526:speci
es_nr:66527:species_nt:66527:NR:AKT93886.1:NT:KT175741.1:VH00444:1:AAA
MMTVHV:1:1309:61101:52190/1
CTATACATCGTAAAAAAGAGCTTGGTCGAAAACTGCCAAAAAAAAAATAAATGGGTCATGTTGTTAATCC
TATAAGTCATCGTATTTATGCAACGTTCTTTCATGATAAAATTTGACATGTTCGTTATCCGTTCTTCTAC
TCTTTTG
>family_nr:555408:family_nt:555408:genus_nr:66526:genus_nt:66526:speci

es_nr:66527:species_nt:66527:NR:AKT93886.1:NT:KT175741.1:VH00444:1:AAA
MMTVHV:1:1309:61101:52190/2
TGCAAACTTAAAATAAACAAAAGAGTAGAAGAACGGATAACGAACATGTCAAATTTTATCATGAAAGAAC
GTTGCATAAATACGATGACTTATAGGATTAACAACATGACCCATTTATTTTTTTTTGGCAGTTTTCGAC
CAAGCTC
>family_nr:555408:family_nt:555408:genus_nr:66526:genus_nt:66526:speci
es_nr:66527:species_nt:66527:NR:AKT93886.1:NT:KT175741.1:VH00444:1:AAA
MMTVHV:1:1604:41825:50751/1
TGGACGTGGACTCTTCTCTCCATAACCAGGCTTTCGGTTACCCATTAACCAAGGGCGTGAATCCTTCTCT
CCGTAACCAGATACATTTTTGCCATGACTAGCCGATGAGAATGAAAAGGGCACACTCGACTTTGAACTAA
AAGAAGA
>family_nr:555408:family_nt:555408:genus_nr:66526:genus_nt:66526:speci
es_nr:66527:species_nt:66527:NR:AKT93886.1:NT:KT175741.1:VH00444:1:AAA
MMTVHV:1:1604:41825:50751/2
TTTCGAAAAATAAAGTAGTGTCGTCGAGTCGCGATGGTCCAAAATTTCCTTCTAAATTTAACAAGGGTCC
TAAGTCTGACTTTGTTTCGTTTTTAGCCCGAAATCCTCATCTTCTTTTAGTTCAAAGTCGAGTGTGCCC
TTTTCAT
>family_nr:555408:family_nt:555408:genus_nr:66526:genus_nt:66526:speci
es_nr:66527:species_nt:66527:NR:AKT93886.1:NT:KT175741.1:VH00444:1:AAA
MMTVHV:1:1607:50800:47949/1
CAATCATGGACGTGGACTCTTCTCTCCATAACCAGGCTTTCGGTTACCCATTAACCAAGGGCGTGAATCC
TTCTCTCCGTA
>family_nr:555408:family_nt:555408:genus_nr:66526:genus_nt:66526:speci
es_nr:66527:species_nt:66527:NR:AKT93886.1:NT:KT175741.1:VH00444:1:AAA
MMTVHV:1:1607:50800:47949/2
TACGGAGAGAAGGATTCACGCCCTTGGTTAATGGGTAACCGAAAGCCTGGTTATGGAGAGAAGAGTCCAC
GTCCATGATTG
>family_nr:555408:family_nt:555408:genus_nr:66526:genus_nt:66526:speci
es_nr:66527:species_nt:66527:NR:AKT93886.1:NT:KT175741.1:VH00444:1:AAA
MMTVHV:1:2414:68732:11545/1
CTCATCGGCTAGTCATGGCAAAAATGTATCTGGTTACGGAGAGAAGGATTCACGCCCTTGGTTAATGGGT
AACCGAAAGCCTGGTTATGGAGAGAAGAGTCCACGTCCATG
>family_nr:555408:family_nt:555408:genus_nr:66526:genus_nt:66526:speci
es_nr:66527:species_nt:66527:NR:AKT93886.1:NT:KT175741.1:VH00444:1:AAA
MMTVHV:1:2414:68732:11545/2
CATGGACGTGGACTCTTCTCTCCATAACCAGGCTTTCGGTTACCCATTAACCAAGGGCGTGAATCCTTCT
CTCCGTAACCAGATACATTTTTGCCATGACTAGCCGATGAG
>family_nr:555408:family_nt:555408:genus_nr:66526:genus_nt:66526:speci
es_nr:66527:species_nt:66527:NR:AKT93891.1:NT:KT175741.1:VH00444:1:AAA
MMTVHV:1:2404:62484:45621/1
TTAATGTACAAAAATTAGACCGAACTTGAACAAAAGGAACCAACATACGGTTGCCTTGCGTAAACGCGAT
AATAGAAAGACAGTTAGCGATGGCTGTTCAACTAGAAGAAGAAGCACAAAACAACGACGTTTTACTTATC
CAAAACA
>family_nr:555408:family_nt:555408:genus_nr:66526:genus_nt:66526:speci
es_nr:66527:species_nt:66527:NR:AKT93891.1:NT:KT175741.1:VH00444:1:AAA
MMTVHV:1:2404:62484:45621/2
TTTGTCTATTGTTAAACTTCATTCGTTTGAAGGTTTGTTTTGGATAAGTAAAACGTCGTTGTTTTGTGCT
TCTTCTTCTAGTTGAACAGCCATCGCTAACTGTCTTTCTATTATCGCGTTTACGCAAGGCAACCGTATGT
TGGTTCC
>family_nr:555408:family_nt:555408:genus_nr:66526:genus_nt:66526:speci

es_nr:66527:species_nt:66527:NR:AKT93891.1:NT:KT175741.1:VH00444:1:AAA
MMTVHV:1:2505:28665:48991/1
AGAAGCACAAAACAACGACGTTTTACTTATCCAAAACAAACCTTCAAACGAATGAAGTTTAACAATAGAC
AAATTAAACTTACGTAATAAATGTACCAAAAGTGAAGTATTTTTTAATTTATGCGCAG
>family_nr:555408:family_nt:555408:genus_nr:66526:genus_nt:66526:speci
es_nr:66527:species_nt:66527:NR:AKT93891.1:NT:KT175741.1:VH00444:1:AAA
MMTVHV:1:2505:28665:48991/2
CTGCGCATAAATTAAAAAATACTTCACTTTTGGTACATTTATTACGTAAGTTTAATTTGTCTATTGTTAA
ACTTCATTCGTTTGAAGGTTTGTTTTGGATAAGTAAAACGTCGTTGTTTTGTGCTTCT
>family_nr:555408:family_nt:555408:genus_nr:66526:genus_nt:66526:speci
es_nr:66527:species_nt:66527:NR:AKT93891.1:NT:KT175741.1:VH00444:1:AAA
MMTVHV:1:2510:45517:23926/1
AAACTTACGTAATAAATGTACCAAAAGTGAAGTATTTTTTAATTTATGCGCAGGCATTACAAAATAAAGG
GAAAAACAAGATGAAGATGCAGGC
>family_nr:555408:family_nt:555408:genus_nr:66526:genus_nt:66526:speci
es_nr:66527:species_nt:66527:NR:AKT93891.1:NT:KT175741.1:VH00444:1:AAA
MMTVHV:1:2510:45517:23926/2
GCCTGCATCTTCATCTTGTTTTTCCCTTTATTTTGTAATGCCTGCGCATAAATTAAAAAATACTTCACTT
TTGGTACATTTATTACGTAAGTTT
>family_nr:555408:family_nt:555408:genus_nr:66526:genus_nt:66526:speci
es_nr:66527:species_nt:66527:NR:AKT93892.1:NT:KT030672.1:VH00444:1:AAA
MMTVHV:1:1308:71289:6982/2
AAGAACGTTTGTTAAAAAAATTTTGGTTCTTGTTCCTTTGGGCGCTGAGAATATATCTTCGCTTTTATCT
CCTGTTTTGGGACAGTA
>family_nr:555408:family_nt:555408:genus_nr:66526:genus_nt:66526:speci
es_nr:66527:species_nt:66527:NR:AKT93892.1:NT:KT030672.1:VH00444:1:AAA
MMTVHV:1:1509:63923:10182/2
TGATAAGTTAACTTTTGAACAAACATGCTACGAGAAGCAAAACTGTATAATACTAAAATACGATACAACA
GAAGAGTAT
>family_nr:555408:family_nt:555408:genus_nr:66526:genus_nt:66526:speci
es_nr:66527:species_nt:66527:NR:AKT93892.1:NT:KT175739.1:VH00444:1:AAA
MMTVHV:1:1509:63923:10182/1
ATACTCTTCTGTTGTATCGTATTTTAGTATTATACAGTTTTGCTTCTCGTAGCATGTTTGTTCAAAAGTT
AACTTATCA
>family_nr:555408:family_nt:555408:genus_nr:66526:genus_nt:66526:speci
es_nr:66527:species_nt:66527:NR:AKT93892.1:NT:KT175739.1:VH00444:1:AAA
MMTVHV:1:2410:25408:27826/1
TGCTACGAGAAGCAAAACTGTATAATACTAAAATACGATACAACAGAAGAGTATTCGAAACAAACTGGTT
TCGA
>family_nr:555408:family_nt:555408:genus_nr:66526:genus_nt:66526:speci
es_nr:66527:species_nt:66527:NR:AKT93892.1:NT:KT175741.1:VH00444:1:AAA
MMTVHV:1:1308:71289:6982/1
TACTGTCCCAAAACAGGAGATAAAAGCGAAGATATATTCTCAGCGCCCAAAGGAACAAGAACCAAAATTT
TTTTAACAAACGTTCTT
>family_nr:555408:family_nt:555408:genus_nr:66526:genus_nt:66526:speci
es_nr:66527:species_nt:66527:NR:AKT93892.1:NT:KT175741.1:VH00444:1:AAA
MMTVHV:1:2410:25408:27826/2
TCGAAACCAGTTTGTTTCGAATACTCTTCTGTTGTATCGTATTTTAGTATTATACAGTTTTGCTTCTCGT
AGCA
>family_nr:555408:family_nt:555408:genus_nr:66526:genus_nt:66526:speci

es_nr:66527:species_nt:66527:NR:AKT93894.1:NT:KT030671.1:VH00444:1:AAA
MMTVHV:1:2605:38303:2666/1
ATAGCTTCTGTTAGCGCAAACCCTGAAATAGCATAACCAAACAATTCCTTTTGTAGTTTCGAATTGCGTA
CAAACGACAACAAAAGGCTACCAAAAAC
>family_nr:555408:family_nt:555408:genus_nr:66526:genus_nt:66526:speci
es_nr:66527:species_nt:66527:NR:AKT93894.1:NT:KT175741.1:VH00444:1:AAA
MMTVHV:1:1310:58109:53591/1
CTTTTGTTGTCGTTTGTACGCAATTCGAAACTACAAAAGGAATTGTTTGGTTATGCTATTTCAGGGTTTG
CGCTAACAGAA
>family_nr:555408:family_nt:555408:genus_nr:66526:genus_nt:66526:speci
es_nr:66527:species_nt:66527:NR:AKT93894.1:NT:KT175741.1:VH00444:1:AAA
MMTVHV:1:1310:58109:53591/2
TTCTGTTAGCGCAAACCCTGAAATAGCATAACCAAACAATTCCTTTTGTAGTTTCGAATTGCGTACAAAC
GACAACAAAAG
>family_nr:555408:family_nt:555408:genus_nr:66526:genus_nt:66526:speci
es_nr:66527:species_nt:66527:NR:AKT93894.1:NT:KT175741.1:VH00444:1:AAA
MMTVHV:1:2605:38303:2666/2
GTTTTTGGTAGCCTTTTGTTGTCGTTTGTACGCAATTCGAAACTACAAAAGGAATTGTTTGGTTATGCTA
TTTCAGGGTTTGCGCTAACAGAAGCTAT
>family_nr:555408:family_nt:555408:genus_nr:66526:genus_nt:66526:speci
es_nr:66527:species_nt:66527:NR:AKT93896.1:NT:KT175741.1:VH00444:1:AAA
MMTVHV:1:1104:49285:17736/1
CTGCTACGACTCTTTTCGCTGGTATTTCTGCGATAATGCAAACAGATGTGAAAAAAATTATTGCATATTC
TACTTGTAGTCATTTGGGTATTATGCTGCTAATCTGCGGTTTTTCTCAGTACAATGCTGCGCTTTTTCAT
TTGCTTA
>family_nr:555408:family_nt:555408:genus_nr:66526:genus_nt:66526:speci
es_nr:66527:species_nt:66527:NR:AKT93896.1:NT:KT175741.1:VH00444:1:AAA
MMTVHV:1:1104:49285:17736/2
TCCCAAAAACAAAAGCGCTTTAAAAAAAGCATGGTTAAGCAAATGAAAAAGCGCAGCATTGTACTGAGAA
AAACCGCAGATTAGCAGCATAATACCCAAATGACTACAAGTAGAATATGCAATAATTTTTTTCACATCTG
TTTGCAT
>family_nr:555408:family_nt:555408:genus_nr:66526:genus_nt:66526:speci
es_nr:66527:species_nt:66527:NR:AKT93896.1:NT:KT175741.1:VH00444:1:AAA
MMTVHV:1:1304:10373:27561/1
GTGAATCTTGTTCGTCAGCCACCGCATGAATAATGGAACCAGCTCCCAAAAACAAAAGCGCTTTAAAAAA
AGCATGGTTAAGCAAATGAAAAAGCGCAGCATTGTACTGAGAAAAACCGCAGATTAGCAGCATAATACCC
AAATGAC
>family_nr:555408:family_nt:555408:genus_nr:66526:genus_nt:66526:speci
es_nr:66527:species_nt:66527:NR:AKT93896.1:NT:KT175741.1:VH00444:1:AAA
MMTVHV:1:1304:10373:27561/2
AAAAAATTATTGCATATTCTACTTTAGTCATTTGGGTATTATGCTGCTAATCTGCGGTTTTTCTCAGTAC
AATGCTGCGCTTTTTCATTTGCTTAACCATGCTTTTTTTAAAGCGCTTTTGTTTTTGGGAGCTGGTTCCA
TTATTCA
>family_nr:555408:family_nt:555408:genus_nr:66526:genus_nt:66526:speci
es_nr:66527:species_nt:66527:NR:AKT93896.1:NT:KT175741.1:VH00444:1:AAA
MMTVHV:1:1503:64282:26823/1
CGCCAGCCTTGGTATCAACCAAAAAACAAAACAGAAAAAAAACAAAAAGAAGCAGAATTATATCATCAAA
AGTGGCAAACACGACCAAGTATAAAACAACTAATGATGCTACAAATAAGGTAACAAAAACATAGGAAAAA
AAATGAG
>family_nr:555408:family_nt:555408:genus_nr:66526:genus_nt:66526:speci

es_nr:66527:species_nt:66527:NR:AKT93896.1:NT:KT175741.1:VH00444:1:AAA
MMTVHV:1:1503:64282:26823/2
GTCCTTTTGGTATTGTTAATACTCTCCGTGCGTGATCGAAAAAGGTAGGTTTGGGCTTTAGCACTCATTT
TTTTTCCTATGTTTTTGTTACCTTATTTGTAGCATCATTAGTTGTTTTATACTTGGTCGTGTTTGCCACT
TTTGATG
>family_nr:555408:family_nt:555408:genus_nr:66526:genus_nt:66526:speci
es_nr:66527:species_nt:66527:NR:AKT93896.1:NT:KT175741.1:VH00444:1:AAA
MMTVHV:1:1607:71289:38105/1
TGCTCTATGTTTGTAGTTTTTTGTATTTTTTTATCGAGTTTGGGTATAGCGTGTTTCTTTCCTTCAGTGT
TTATAGGTTTTTTAGTTTGTTTTGTT
>family_nr:555408:family_nt:555408:genus_nr:66526:genus_nt:66526:speci
es_nr:66527:species_nt:66527:NR:AKT93896.1:NT:KT175741.1:VH00444:1:AAA
MMTVHV:1:1607:71289:38105/2
AACAAAACAAACTAAAAAACCTATAAACACTGAAGGAAAGAAACACGCTATACCCAAACTCGATAAAAAA
ATACAAAAAACTACAAACATAGAGCA
>family_nr:555408:family_nt:555408:genus_nr:66526:genus_nt:66526:speci
es_nr:66527:species_nt:66527:NR:AKT93896.1:NT:KT175741.1:VH00444:1:AAA
MMTVHV:1:2313:51690:7683/1
GGTTTTTTAGTTTGTTTTGTTATATATACATTGCTTTTTGCGCTTCCTGGTGTGGTTGGTACGGCAGATT
TGCTTGCATTCTTTTTGGTTTTAGGTAATCAAAAACGTTTTTATAAATTCGTGTCTTCGGTTTTAGCAGA
AGATCAT
>family_nr:555408:family_nt:555408:genus_nr:66526:genus_nt:66526:speci
es_nr:66527:species_nt:66527:NR:AKT93896.1:NT:KT175741.1:VH00444:1:AAA
MMTVHV:1:2313:51690:7683/2
GTGAAACAAATAAAACAATTTTAGACAACCAACGATAGAGAGCATGATCTTCTGCTAAAACCGAAGACAC
GAATTTATAAAAACGTTTTTGATTACCTAAAACCAAAAAGAATGCAAGCAAATCTGCCGTACCAACCACA
CCAGGAA
>family_nr:555408:family_nt:555408:genus_nr:66526:genus_nt:66526:speci
es_nr:66527:species_nt:66527:NR:AKT93904.1:NT:KT175741.1:VH00444:1:AAA
MMTVHV:1:2202:59927:14177/1
AATATGAGGAAATTCCTACAACAAGTTGGTTTGCATAAGGTGCGGACAAAAATAGCGTTTGGGGAAATAA
CACAAGAAGATTTAAATAAACAGTTCCGTACACATAGGCGTAAAGGTATTTAAATTTGTATAACAACGCA
AGTATAC
>family_nr:555408:family_nt:555408:genus_nr:66526:genus_nt:66526:speci
es_nr:66527:species_nt:66527:NR:AKT93904.1:NT:KT175741.1:VH00444:1:AAA
MMTVHV:1:2202:59927:14177/2
GTGAGAGTCCGTATACTTGCGTTGTTATACAAATTTAAATACCTTTACGCCTATGTGTACGGAACTGTTT
ATTTAAATCTTCTTGTGTTATTTCCCCAAACGCTATTTTTGTCCGCACCTTATGCAAACCAACTTGTTGT
AGGAATT
>family_nr:555408:family_nt:555408:genus_nr:66526:genus_nt:66526:speci
es_nr:66527:species_nt:66527:NR:AKT93904.1:NT:KT175741.1:VH00444:1:AAA
MMTVHV:1:2411:26430:17755/1
CTTATGCGTTGGAGAAACAAAAGGCACCCCCACCTTTTTCGATGCTGGCCGCAAAGAAGTGGTTAAAAAG
AAAGCTCTTGATACAATGCAAACGTCCGCAGTTGCAACCAACAGCGAGAAGAGAACAACAGCACGATATA
ACAACGT
>family_nr:555408:family_nt:555408:genus_nr:66526:genus_nt:66526:speci
es_nr:66527:species_nt:66527:NR:AKT93904.1:NT:KT175741.1:VH00444:1:AAA
MMTVHV:1:2411:26430:17755/2
GTGTACGGAACTGTTTATTTAAATCTTCTTGTGTTATTTCCCCAAACGCTATTTTTGTCCGCACCTTATG
CAAACCAACTTGTTGTAGGAATTTCCTCATATTATACTTTGTTATATCGTGCTGTTGTTCTCTTCTCGCT

GTTGGTT
>family_nr:555408:family_nt:555408:genus_nr:66526:genus_nt:66526:species_nr:66527:species_nt:66527:NR:AKT94946.1:NT:KT175739.1:VH00444:1:AAAMMTVHV:1:1112:44324:35077/1
CTCTTTAGCTGTGGAGCAGTTGCTTAATATACAGGTACCGCTTCGGGCACAATATATACGTGTTATGTTC
AGCGAGCTTACTCGTCTTCTGAATCATTTACTTGCGTTAACGACACATGCTTTAGATGTTGGTGCACTTA
CAGATCG
>family_nr:555408:family_nt:555408:genus_nr:66526:genus_nt:66526:species_nr:66527:species_nt:66527:NR:AKT94946.1:NT:KT175739.1:VH00444:1:AAAMMTVHV:1:1112:44324:35077/2
GTAAGTGCACCAACATCTAAAGCATGTGTCGTTAACGCAAGTAAATGATTCAGAAGACGAGTAAGCTCGC
TGAACATAACACGTATATATTGTGCCCGAAGCGGTACCTGTATATTAAGCAACTGCTCCACAGCTAAAGA
GAGATCG
>family_nr:555408:family_nt:555408:genus_nr:66526:genus_nt:66526:species_nr:66527:species_nt:66527:NR:AKT94946.1:NT:KT175739.1:VH00444:1:AAAMMTVHV:1:1612:72216:9122/1
CCGCTTCGGGCACAATATATACGTGTTATGTTCAGCGAGCTTACTCGTCTTCTGAATCATTTACTTGCGT
TAACGACACATGCTTTAGATGTTGGTGCACTTACTCCATTTTTATGGGGG
>family_nr:555408:family_nt:555408:genus_nr:66526:genus_nt:66526:species_nr:66527:species_nt:66527:NR:AKT94946.1:NT:KT175739.1:VH00444:1:AAAMMTVHV:1:1612:72216:9122/2
CCCCCATAAAAATGGAGTAAGTGCACCAACATCTAAAGCATGTGTCGTTAACGCAAGTAAATGATTCAGA
AGACGAGTAAGCTCGCTGAACATAACACGTATATATTGTGCCCGAAGCGG
>family_nr:555408:family_nt:555408:genus_nr:66526:genus_nt:66526:species_nr:66527:species_nt:66527:NR:AKT94946.1:NT:KT175741.1:VH00444:1:AAAMMTVHV:1:1610:47846:32426/1
CTTAAATATAACATTTATCTGAAACTGTTCTAAGGGATTATTCACAAATTTAACGATCGACTTCACCAAA
AACAATATCCTGAGTACCTATAATTGTCACAACATCAGCTACCATGTGATTATGCGCCATAAAATCCAAC
CCTTGCA
>family_nr:555408:family_nt:555408:genus_nr:66526:genus_nt:66526:species_nr:66527:species_nt:66527:NR:AKT94946.1:NT:KT175741.1:VH00444:1:AAAMMTVHV:1:1610:47846:32426/2
TAAGGGCGAATTTGGTGTTTTTCTTTATTCTGACGGTACTAATCGTCCGTATAGGTGCAAGATCCGTGCC
CCAGGGTTTTTTCATTTGCAAGGGTTGGATTTTATGGCGCATAATCACATGGTAGCTGATGTTGTGACAA
TTATAGG
>family_nr:555408:family_nt:555408:genus_nr:66526:genus_nt:66526:species_nr:66527:species_nt:66527:NR:AKT94946.1:NT:KT175741.1:VH00444:1:AAAMMTVHV:1:2609:15050:6301/1
CATAGAAAATTTCATAAACGAACGAAAAGGTGGAACAAGTTTATTGTCAGCAAGTTTATATAGCCCCTGA
GGCATCGAATTCAAACATTGATGAATAACCGACAAACTTTCGCGCATTTCGAAAACACGCAACAAATAGC
GGGTATA
>family_nr:555408:family_nt:555408:genus_nr:66526:genus_nt:66526:species_nr:66527:species_nt:66527:NR:AKT94946.1:NT:KT175741.1:VH00444:1:AAAMMTVHV:1:2609:15050:6301/2
ATACCCTGCATTTTCAGGTACACTAAACGATTCACTATAATATTTAAAATGATGGATTAAAGATTCCATA
GAAAATTTAATGTGCCCGTGGGTGTGAAAGGGGATTGTTATACCCGCTATTTGTTGCGTGTTTTCGAAAT
GCGCGAA
>family_nr:555408:family_nt:555408:genus_nr:66526:genus_nt:66526:species_nr:66527:species_nt:66527:NR:AKT94976.1:NT:KT175741.1:VH00444:1:AAAMMTVHV:1:1605:47562:11848/1

ACGCGGAATTCTTAAAGACCAAAGATTTTTTAAACAAAGTTTTAAAACGAGCAAGTCGTAACTCAAATAA
GCGGCGCCGTTGTTGAATAACTTGCCGCCACATAGACCCAGCCGGCAAAGCTTTAACTGCTTTTGTAACA
AAACGAA
>family_nr:555408:family_nt:555408:genus_nr:66526:genus_nt:66526:speci
es_nr:66527:species_nt:66527:NR:AKT94976.1:NT:KT175741.1:VH00444:1:AAA
MMTVHV:1:1605:47562:11848/2
CATGATAAAAAAATTTCGTAAATTTCATTTTCATTTTATAGGTCAGTTCAGATTTTTAAGTGGTTTTAAA
CAAGTTCGAAATTTTCGTTTTGTTACAAAAGCAGTTAAAGCTTTGCCGGCTGGGTCTATGTGGCGGCAAG
TTATTCA
>family_nr:555408:family_nt:555408:genus_nr:66526:genus_nt:66526:speci
es_nr:66527:species_nt:66527:NR:AKT94976.1:NT:KT175741.1:VH00444:1:AAA
MMTVHV:1:2401:51955:46681/1
CCGGAAACGTTTCGCCGTCTCACCTTTTCCTTATTTAACTCCTTTATTACGTTCCGTAAAGTGTCTAGAA
CGTTTTGGACGGCCTAATAAAC
>family_nr:555408:family_nt:555408:genus_nr:66526:genus_nt:66526:speci
es_nr:66527:species_nt:66527:NR:AKT94976.1:NT:KT175741.1:VH00444:1:AAA
MMTVHV:1:2401:51955:46681/2
GTTTATTAGGCCGTCCAAAACGTTCTAGACACTTTACGGAACGTAATAAAGGAGTTAAATAAGGAAAAGG
TGAGACGGCGAAACGTTTCCGG
>family_nr:555408:family_nt:555408:genus_nr:66526:genus_nt:66526:speci
es_nr:66527:species_nt:66527:NR:AKT95010.1:NT:KT175738.1:VH00444:1:AAA
MMTVHV:1:2205:45858:46908/1
TTCCAAAATTTTTTTTTTTATATTCGTTGGCGAGTCATTTGCGGTTTGTTTATATTTCTATATTTCTTTT
GTCGACTATGTTACTGTTATG
>family_nr:555408:family_nt:555408:genus_nr:66526:genus_nt:66526:speci
es_nr:66527:species_nt:66527:NR:AKT95010.1:NT:KT175741.1:VH00444:1:AAA
MMTVHV:1:2205:45858:46908/2
CATAACAGTAACATAGTCGACAAAAGAAATATAGAAATATAAACAAACCGCAAATGACTCGCCAACGAAT
ATAAAAAAAAAAATTTTGGAA
>family_nr:555408:family_nt:555408:genus_nr:66526:genus_nt:66526:speci
es_nr:66527:species_nt:66527:NR:AKU37119.1:NT:KT175741.1:VH00444:1:AAA
MMTVHV:1:2604:76856:24097/2
CATTACGGCCAGGTTTAGCCTTTTTTTTACGCAACCCAATTTTAGGGTCTTGCATTTTCTAATAGGATCA
AATATATGCGCTTTCGTGTGGGTAAAAAACAACAAATCTAATGACGTTTTTTTCTTTGTTACAATCACGA
TACGTTA
>family_nr:555408:family_nt:555408:genus_nr:66526:genus_nt:66526:speci
es_nr:66527:species_nt:66527:NR:AKU37120.1:NT:KT030671.1:VH00444:1:AAA
MMTVHV:1:1603:65589:8630/1
AACACACCAAACGTATTACGTCAACTAAGTGGGACAAAAAAACCCGAAACTTTAACAAAAGGTGACGCAA
TTCGCTGACGATAAAGACCATCGTAAAAAAAACGAATTTTATCTGAAATCCAATCCAAATTGAGCACGCT
ATTTGTG
>family_nr:555408:family_nt:555408:genus_nr:66526:genus_nt:66526:speci
es_nr:66527:species_nt:66527:NR:AKU37120.1:NT:KT030671.1:VH00444:1:AAA
MMTVHV:1:1603:65589:8630/2
CAAAAATTGTTTGCTTCGGAAATTTCTGGTAATGTTATAGACCTCTGTCCTGTCGGGGCATTAACTTCAA
AACCCTATGCTTTTACTGCACGTCCTTGAGAATTACGCAGTGTTTTTACGGTAGATCCTTTAGATTCTTT
ATGTTCT
>family_nr:555408:family_nt:555408:genus_nr:66526:genus_nt:66526:speci
es_nr:66527:species_nt:66527:NR:YP_009163124.1:NT:KT175741.1:VH00444:1
:AAAMMTVHV:1:2606:74583:35796/1

```
TTTTCCTTAAAGGATAACCGTCAAAACCATAATCAGTTAAAATACGTCTCAAATCA
>family_nr:555408:family_nt:555408:genus_nr:66526:genus_nt:66526:speci
es_nr:66527:species_nt:66527:NR:YP_009163124.1:NT:KT175741.1:VH00444:1
:AAAMMTVHV:1:2606:74583:35796/2
TGATTTGAGACGTATTTTAACTGATTATGGTTTTGACGGTTATCCTTTAAGGAAAA
>family_nr:555408:family_nt:555408:genus_nr:66526:genus_nt:66526:speci
es_nr:66527:species_nt:66527:NR:YP_009163124.1:NT:KT175741.1:VH00444:1
:AAAMMTVHV:1:2611:30331:48574/1
CAATATGCATTCGCTCATCATCATAACGTAATTCAATATAACCAGTCAATGGAAAATTTTTCCTTAAAGG
ATAACCGTCAAAACCGTAATCAGTTAAAATACGTCTCAAATCAGGATGACCGTGAAAAAAAATACCAAAC
ATATCCC
>family_nr:555408:family_nt:555408:genus_nr:66526:genus_nt:66526:speci
es_nr:66527:species_nt:66527:NR:YP_009163130.1:NT:KP888565.1:VH00444:1
:AAAMMTVHV:1:2502:26771:17793/2
CTAAAAATAAAACGGATGATATGAGGGTCCTCCGACATATATTCCAAGGAATAAAGATGTACGAG
>family_nr:555408:family_nt:555408:genus_nr:66526:genus_nt:66526:speci
es_nr:66527:species_nt:66527:NR:YP_009163130.1:NT:KT030671.1:VH00444:1
:AAAMMTVHV:1:2502:26771:17793/1
CTCGTACATCTTTATTCCTTGGAATATATGTCGGAGGACCCTCATATCATCCGTTTTATTTTTAG
>family_nr:555408:family_nt:555408:genus_nr:66526:genus_nt:66526:speci
es_nr:66527:species_nt:66527:NR:YP_009163133.1:NT:KT175741.1:VH00444:1
:AAAMMTVHV:1:1610:27188:49861/1
CTTCACGGCCAGGAGGTCGGCGTAATAACAACGACATTTGGCGGTAAAGCATATCCAGGAGATGTGTTTT
ATTTGCATTCGCGTTTGTTAGAACGTTCAGCAAAATTG
>family_nr:555408:family_nt:555408:genus_nr:66526:genus_nt:66526:speci
es_nr:66527:species_nt:66527:NR:YP_009163133.1:NT:KT175741.1:VH00444:1
:AAAMMTVHV:1:1610:27188:49861/2
CAATTTTGCTGAACGTTCTAACAAACGCGAATGCAAATAAAACACATCTCCTGGATATGCTTTACCGCCA
AATGTCGTTGTTATTACGCCGACCTCCTGGCCGTGAAG
>family_nr:555408:family_nt:555408:genus_nr:66526:genus_nt:66526:speci
es_nr:66527:species_nt:66527:NR:YP_009163133.1:NT:KT175741.1:VH00444:1
:AAAMMTVHV:1:2608:66006:19383/1
CATCACCTTATCACCAGGTTTAACGCTACGATCAGAACCGAATAAAACAGCACCAATATTTTTTTGGTTT
AGATTCAAGGCCATACCCTTTAGCAT
>family_nr:555408:family_nt:555408:genus_nr:66526:genus_nt:66526:speci
es_nr:66527:species_nt:66527:NR:YP_009163133.1:NT:KT175741.1:VH00444:1
:AAAMMTVHV:1:2608:66006:19383/2
ATGCTAAAGGGTATGGCCTTGAATCTAAACCAAAAAAATATTGGTGCTGTTTTATTCGGTTCTGATCGTA
GCGTTAAACCTGGTGATAAGGTGATG
>family_nr:555408:family_nt:555408:genus_nr:66526:genus_nt:66526:speci
es_nr:66527:species_nt:66527:NR:YP_009163133.1:NT:KT175741.1:VH00444:1
:AAAMMTVHV:1:2609:61575:8213/1
GTTTTATTTGCATTCGCGTTTGTTAGAACGTTCAGCAAAATTGCTTTCTGGTGGTTCTTTAACCGCTTTA
CCTATTGTGGAAACGCAGGCTGGTGATGTTTCTGCTTATATTCCAACAAATGTAATTTCGATTACGGATG
GTCAAAT
>family_nr:555408:family_nt:555408:genus_nr:66526:genus_nt:66526:speci
es_nr:66527:species_nt:66527:NR:YP_009163133.1:NT:KT175741.1:VH00444:1
:AAAMMTVHV:1:2609:61575:8213/2
AAAACAAATTAGTTTCCAGGAAAATTTGACCATCCGTAATCGAAATTACATTTGTTGGAATATAAGCAGA
AACATCACCAGCCTGCGTTTCCACAATAGGTAAAGCGGTTAAAGAACCACCAGAAAGCAATTTTGCTGAA
```

CGTTCTA
>family_nr:555408:family_nt:555408:genus_nr:66526:genus_nt:66526:speci
es_nr:66527:species_nt:66527:NR:YP_009163136.1:NT:KT175741.1:VH00444:1
:AAAMMTVHV:1:1112:44798:38049/1
ATTGGCGCAGAAAAAAAGTTCCGACTAATAATACAGCAGCACTTCAAGGTAATCCTCAACTTAAGGGGAC
TTGTGTTAAGCTGCGTATTGTAAAACCAAAAAAACCTAATTCAGCGCAACGTAAAATCGCGAAAG
>family_nr:555408:family_nt:555408:genus_nr:66526:genus_nt:66526:speci
es_nr:66527:species_nt:66527:NR:YP_009163136.1:NT:KT175741.1:VH00444:1
:AAAMMTVHV:1:1112:44798:38049/2
CTTTCGCGATTTTACGTTGCGCTGAATTAGGTTTTTTTGGTTTTACAATACGCAGCTTAACACAAGTCCC
CTTAAGTTGAGGATTACCTTGAAGTGCTGCTGTATTATTAGTCGGAACTTTTTTTCTGCGCCAAT
>family_nr:555408:family_nt:555408:genus_nr:66526:genus_nt:66526:speci
es_nr:66527:species_nt:66527:NR:YP_009163136.1:NT:KT175741.1:VH00444:1
:AAAMMTVHV:1:1406:30956:16713/1
CCAAAACTTGACGCTTTGTTGATAAACGAACTTTCGCGATTTTACGTTTCACCAGTTATTAAGATATTGG
CGCAGAAAAAAAGTTCCGACTAATAATACAGCAGCACTTCAAGGTAATCCTCAACTTAAGGGGACTTGTG
TTAAGCT
>family_nr:555408:family_nt:555408:genus_nr:66526:genus_nt:66526:speci
es_nr:66527:species_nt:66527:NR:YP_009163136.1:NT:KT175741.1:VH00444:1
:AAAMMTVHV:1:1406:30956:16713/2
GCGATTTTACGTTGCGCTGAATTAGGTTTTTTTGGTTTTACAATACGCAGCTTAACACAAGTCCCCTTAA
GTTGAGGATTACCTTGAAGTGCTGCTGTATTATTAGTCGGAACTTTTTTTCTGCGCCAATATCTTAATAA
CTGGTGA
>family_nr:555408:family_nt:555408:genus_nr:66526:genus_nt:66526:speci
es_nr:66527:species_nt:66527:NR:YP_009163137.1:NT:KT175739.1:VH00444:1
:AAAMMTVHV:1:1214:16205:36761/1
CTACTCCGACGCCATAGATTTCGAATATACTTATTAGCTAATATGTATATTCGAAATCTATGGCGTCGGA
GTAGCCGTACGCGAACAAGGCGTTTACGTCTAAAAGATTTGGATTTTTCAACACTTAAACTAAGTAAACG
CATGAAG
>family_nr:555408:family_nt:555408:genus_nr:66526:genus_nt:66526:speci
es_nr:66527:species_nt:66527:NR:YP_009163151.1:NT:KT030671.1:VH00444:1
:AAAMMTVHV:1:1512:42166:47533/1
CACGTTTTTGTTATTATTCGAAATTATCAATTGCTGGGAATTGTCGGATGTGTTTAATAGAGGAAGAAGA
AAGCCCAAAGCTCGTGGCTTCG
>family_nr:555408:family_nt:555408:genus_nr:66526:genus_nt:66526:speci
es_nr:66527:species_nt:66527:NR:YP_009163151.1:NT:KT030673.1:VH00444:1
:AAAMMTVHV:1:1512:42166:47533/2
CGAAGCCACGAGCTTTGGGCTTTCTTCTTCCTCTATTAAACACATCCGACAATTCCCAGCAATTGATAAT
TTCGAATAATAACAAAAACGTG
>family_nr:555408:family_nt:555408:genus_nr:66526:genus_nt:66526:speci
es_nr:66527:species_nt:66527:NR:YP_009163151.1:NT:KT030673.1:VH00444:1
:AAAMMTVHV:1:2407:19330:49275/2
CGAAACTTTAACAAAAGGTGACGCAATTCGCTGACGATAAAGACCATCGTAAAAAAAACGAATTTTATCT
GAAATCCAATCCAAATTGAGCACGCTATTTGTGCGCGGTAGTATACGAGCAATAG
>family_nr:555408:family_nt:555408:genus_nr:66526:genus_nt:66526:speci
es_nr:66527:species_nt:66527:NR:YP_009163151.1:NT:KT175741.1:VH00444:1
:AAAMMTVHV:1:2407:19330:49275/1
CTATTGCTCGTATACTACCGCGCACAAATAGCGTGCTCAATTTGGATTGGATTTCAGATAAAATTCGTTT
TTTTTACGATGGTCTTTATCGTCAGCGAATTGCGTCACCTTTTGTTAAAGTTTCG
>family_nr:809:family_nt:555408:genus_nr:810:genus_nt:66526:species_nr

:813:species_nt:66527:NR:CRH45931.1:NT:KT175741.1:VH00444:1:AAAMMTVHV:
1:1607:54455:21049/1
CCTGGAGCTGCAGGAGGTTCCAAGGGTTGGGCTGTTCGCCCATTAAAGTGGTACGTGAGTTGGGTTTAGA
ACGTTGTGAAACAGTTCGGTTCCTATCTTTTACTTATTTTAGAATAATAAAATTATATCCCTCGTACGAG
AGGATTG
>family_nr:809:family_nt:555408:genus_nr:810:genus_nt:66526:species_nr
:813:species_nt:66527:NR:CRH45931.1:NT:KT175741.1:VH00444:1:AAAMMTVHV:
1:1607:54455:21049/2
ATTCTAAATTTATGTTTAGCTTTTCAGCTTACCATTTAGTAAAATAACTGAAGTACCAGTGACATACACA
CCCCAATCCTCTCGTACGAGGGATATAATTTTATTATTCTAAAATAAGTAAAAGATAGGAACCGAACTGT
TTCACAA
>family_nr:91896:family_nt:555408:genus_nr:4169:genus_nt:66526:species
_nr:4170:species_nt:66527:NR:GER39135.1:NT:KT175741.1:VH00444:1:AAAMMT
VHV:1:1209:8536:38484/1
TAAAATATCTTCAATTTGTTCATAACTAGATCACTCGGTTTCGAGTTTGAATAAAACAACTGCATTCCCC
GTTAGGGAATTACTTTCATTACTTTTATACAAATTGCTTTTTTATTCAATTTGTTGAC
>family_nr:91896:family_nt:555408:genus_nr:4169:genus_nt:66526:species
_nr:4170:species_nt:66527:NR:GER39135.1:NT:KT175741.1:VH00444:1:AAAMMT
VHV:1:1209:8536:38484/2
GTCAACAAATTGAATAAAAAAGCAATTTGTATAAAAGTAATGAAAGTAATTCCCTAACGGGGAATGCAGT
TGTTTTATTCAAACTCGAAACCGAGTGATCTAGTTATGAACAAATTGAAGATATTTTA
>family_nr:91896:family_nt:555408:genus_nr:4169:genus_nt:66526:species
_nr:4170:species_nt:66527:NR:GER39135.1:NT:KT175741.1:VH00444:1:AAAMMT
VHV:1:1504:59227:14536/1
AGTTTGATTAGCCTTTCACCCCTAGCTACAAATCTTCCCAGTATATTGCTACATACACGGGTGCAATCCT
CCGATACGTTTTTAAAATATCTTCAATTTGTTCATAACTAGATCACTCGGTTTCGAGTTTGAATAAAACA
ACTGCAT
>family_nr:91896:family_nt:555408:genus_nr:4169:genus_nt:66526:species
_nr:4170:species_nt:66527:NR:GER39135.1:NT:KT175741.1:VH00444:1:AAAMMT
VHV:1:1504:59227:14536/2
ATAATGGGTCAACAAATTGAATAAAAAAGCAATTTGTATAAAAGTAATGAAAGTAATTCCCTAACGGGGA
ATGCAGTTGTTTTATTCAAACTCGAAACCGAGTGATCTAGTTATGAACAAATTGAAGATATTTTAAAAAC
GTATCGG
